# Implementation of the Macao dementia policy: a scoping review for the way forward

**DOI:** 10.3389/fpubh.2024.1400172

**Published:** 2024-07-15

**Authors:** Pou Kuan Tang, Zhifeng Cen, Yu Zheng, Junnan Shi, Hao Hu, Carolina Oi Lam Ung

**Affiliations:** ^1^State Key Laboratory of Quality Research in Chinese Medicine, Institute of Chinese Medical Science, University of Macau, Macao, China; ^2^Faculty of Health Sciences, Department of Public Health and Medicinal Administration, University of Macau, Macao, China

**Keywords:** dementia, policy, person-centered care, scoping review, Macao

## Abstract

**Background:**

The implementation of dementia policy is a complex process of translating policy goals to actions to address the changing needs of people living with dementia. Leveraging on others’ experiences would help policy decision-makers and actors better prepare for the challenges.

**Purpose:**

This study explored the development, the implementation and the impact of the dementia policy in Macao, a “role model” recognized by the Alzheimer’s Disease International.

**Methods:**

A scoping review of policies, strategies, and news articles, as well as scholarly work from 6 scientific databases dated till March 2023 was conducted under the guidance of the Health Policy Triangle Framework.

**Results:**

According to 284 documents, the dementia policy in Macao, driven by government leadership and supported with public-private partnership, aimed to integrate health and social services to achieve the goals of “*Early prevention, Early detection, Early diagnosis, Early treatment and Early support*.” Promoting the preparedness according to the dementia burden trajectory, empowering the public and the service providers with training and education, and encouraging services-related research were among the key actions. With major changes in dementia care configuration, a dementia service network, a dementia-friendly community and a one-stop service model for disease screening, diagnosis, treatment and support have been developed.

**Discussion:**

Reconfiguring existing resources in the health and social services to form an integrated service network at the community level could be considered a priority of action. Continuous engagement, collaboration and empowerment at different levels across these sectors is crucial for the sustainability of a dementia policy.

## 1 Introduction

Dementia is the seventh leading cause of death affecting 55 million individuals and representing one of the greatest health challenges globally (1). It refers to a group of symptoms featured with a gradual loss of cognitive functioning such as thinking, remembering, and reasoning that interferes with a person’s daily life and activities (2). The nature of the disease progression compounded by the increasing prevalence associated with the aging population induces profound burden not only to the patients and their caregivers, but also to the society (3).

To tackle the dementia challenges, the World Health Assembly approved the *Global Action Plan on the Public Health Response to Dementia for 2017–2025* recommending actions at national levels (4). The Alzheimer’s Disease International (ADI) also published *The World Alzheimer Report* in an effort to promote systematic approach to dementia care (5). At present, at least 48 countries and territories (6) have since developed their own dementia plans to address the needs of people living with dementia at regional [e.g., the *Regional Plan of Action on Dementia* firstly launched by The Pan-American Health Organization in 2015 (7)], national [e.g., England being the first to launch *Living Well with Dementia: a National Dementia Strategy* in 2009 (8)] and sub-national levels [e.g., the *Dementia Plan for Flanders 2016–2019* (9)].

Successful policy implementation depends on the complex process that ensures the translation of policy goals to actions and changes in the practical setting (10). This is especially the case for implementing dementia policy whereby different levels of government, institution and individuals from different sectors (e.g., health and social welfare) are involved. To have different stakeholders acquire a common understanding of the policy intent, appreciate each other’s perspectives and find common grounds for communication and collaboration often present major challenges during the initial phase of implementation (11). During the implementation process, many countries and organizations are still trying to overcome some very common practical challenges such as negative perceptions of dementia care (12), caregivers’ burden (e.g., limited chances for social interaction with other caregivers) (6), insufficient dementia knowledge and skills among healthcare professionals (13), and insufficient coordination among different healthcare settings (14). Well-informed implementation strategies and actions of dementia policies play an important role in managing foreseeable challenges to achieve intended outcomes (15). Leveraging on others’ experiences of implementing dementia policies would help the policy decision-makers and actors to better understand the political, economic, infrastructure and conceptual constructivism contexts that might facilitate or hinder successful implementation and continuous optimization of dementia policies (16).

Macao has a population of 680,000 with the population aged 65 years or above recorded at 13.7% in 2023. Featuring an increasing trend of aging population and an average life expectancy of 85 years (17), the old-age population proportion is expected to reaching 22.4% in 2031 (18). According to reported figures, the estimated prevalence and incidence rate of dementia in population aged 60 years and above were 4.94% and 10.69‰ respectively (19–21). How to effectively manage dementia at a population level through the implementation of the dementia policy continues to be a political and social focus over time. In response, the government positioned dementia a priority in public health and announced the Macao Dementia Policy in 2016 (22). This was followed by a series of programs and actions to build a dementia-friendly community and to establish a comprehensive dementia service network through interdepartmental and interprofessional collaboration (23).

Being the 27th globally to implement a specific dementia policy (22) and 5 years after the launch of the dementia policy, Macao was rated stage 5A for the successful implementation of the policy and recognized as a “role model” by the ADI in 2021 (24). This recognition underscores its potential as a valuable case study for empirical investigations into policy implementation (24, 25). Furthermore, from the local perspective, there remains a pressing need for empirical investigations to evaluate the effectiveness and impact of the dementia policy in Macao for its sustainable development and continuous improvement such as in the cases of Ireland (26) and England (27). Considering the scarcity of comprehensive analysis of implementing dementia policies, this study aimed to explore the development of the dementia policy in Macao, how it was implemented, and the impact of its implementation on dementia care. It is anticipated that the study findings will be useful in generating ideas on processes and strategies for the optimization of dementia policy implementation in Macao and provide valuable references to policy-decision makers in other contexts alike.

## 2 Methods

In order to map existing literature relevant to the research question and to identify key areas for informing policy making and implementation strategies (28), this study employed a scoping review approach to identify as much literature as possible in this specific area. A scoping review allows for a broad examination of the literature, encompassing a wide range of study designs and sources. This approach is particularly beneficial for analyzing Macao’s dementia policy, where existing research and resources are diverse and multidisciplinary. This review was conducted and reported in compliance with the guidance of the Preferred Reporting Items for Systematic reviews and Meta-analysis extension for Scoping Reviews (PRISMA-ScR) checklist (29).

### 2.1 Types and sources of documents included for document analysis

The types of documents included in this study were a mix of formal documents, gray literature, informal documents, as well as scholarly work. By consulting a mix of document types and sources, a rich insight into how policy was formulated, launched and implemented, how policy actors interacted, and the changes brought about the policy could be yielded (28). The sources of each document source are listed in Table 1.

**Table 1:** Types and sources of documents included in this study.

Category	Examples	Sources
Formal documents	Official policies, laws or strategies	The government portal of Macao special administrative region (SAR) (https://www.gov.mo/en/); The health bureau, Macao SAR government (https://www.ssm.gov.mo/portal/); The social welfare bureau, Macao SAR government (https://www.ias.gov.mo/en/home); The Macao SAR older adult service information network (https://www.ias.gov.mo/en/swb-services/services/elderly); The Macao dementia friendly community (https://www.ssm.gov.mo/apps1/mdfc/ch.aspx#l29207).
Gray literature	Organizational materials or evaluation reports produced outside formal publication channels such as newspapers and forum discussion	The World Health Organization (Alzheimer’s Disease International) The Alzheimer’s Disease International (https://www.alzint.org/) The Macau Alzheimer’s Disease Association (MADA) (http://www.mada.org.mo/?lang=en) The Kiang Wu Nursing College of Macau (KWNC) (http://www2.kwnc.edu.mo/?page_id=5481) Websites of non-government organizations supporting the implementation of Macao Dementia Policy Local mainstream newspapers listed on the government website (https://gcs.gov.mo/)
Informal documents	PowerPoint presentations and conference notes	The google search engine
Scholarly work	Scientific or peer-reviewed publications of reviews and all types of primary studies	4 English databases (including PubMed, SCOPUS, MEDLINE, and Web of Science) 2 Chinese databases [including Chinese National Knowledge Infrastructure (CNKI), and WanFang]

### 2.2 Search strategy

Separate search strategies were established refined through team discussion for each data source. Keywords used to identify policy documents and other resources targeting on dementia patient in Chinese are *shi zhi* (dementia), *chidai zheng* (dementia), *a zi hai mo* (Alzheimer’s disease), and in English are dementia, neurodegenerative and Alzheimer’s disease. The search strategy of electronic databases is provided in the Supplementary material.

### 2.3 Eligibility criteria

Documents dated or published on or before 31 March 2023 were included for analysis in this study if they: (1) involved Macao, either alone or as part of a larger region; (2) mentioned dementia risk reduction, disease prevention or disease management; and (3) written in English or Chinese. Documents were excluded if: (1) they were study protocol that did not present empirical data; (2) they did not mention dementia; or (3) the full text was not available to the public.

### 2.4 Document selection

The title and, whenever applicable, the abstract of the documents identified in the search were first screened separately by two authors (PKT and CZ). Following removal of duplicate documents, the remaining articles were again screened for inclusion based on full text separately by PKT and CZ. The quality of the screening process was ensured by comparing the screening results between PKT and CZ. Any conflicts in the screening results were discussed and resolved among PKT and CZ, and were later confirmed by a third author (COLU). Moreover, studies considered for exclusion were finalized based on consensus among PKT, CZ and COLU.

### 2.5 Data extraction and analysis

The READ (Ready materials, extract data, analyze data, and distil findings) approach, one of the most commonly used methods that guide the process of document analysis for qualitative policy research step-by-step, was employed for systematic document analysis (30). First, each included document was read thoroughly for familiarization purposes by two authors (PKT and CZ). Then, data was extracted by PKT and CZ using a standardized format in an Excel spreadsheet. Each of the eligible documents were tabulated and summarized to extract data from included documents according to the 4 domains in the Health Policy Triangle Framework (HPTF) (31). In addition, the documents were sorted by their date of publication and a timeline of main events was created to trace processes across time.

The HPTF was created by Walt et al., and is one of the most extensively used frameworks for policy analysis. There are 4 main constructs of the HPTF: (1) the “*Content*” of policies which may include policy objectives, operational policies, legislation, regulations, guidelines, etc.; (2) the “*Actors*” involved in policy changes which may include influential individuals, groups and organizations; (3) the “*Context*” impacting on the policy making which may include social, economic, political, cultural, environmental conditions and other systematic factors (31, 32); and (4) the “*Process*” which refers to the way in which policies are initiated, developed, communicated, implemented and evaluated. Collectively, the HPTF provides a guiding framework for navigating the implementation of complex policies in either retrospective and prospective studies (33), and had been widely employed in health policy research including health reform, mental health, and reproductive health (32).

To ensure the quality and consistency of data extraction, data extraction for a random of 5 documents was performed separately by PKT, CZ and COLU, and the results were compared to assess the accuracy and consistency of data extracted by the team. Furthermore, data extracted from another 20% of the included documents was checked by COLU and HH to ensure the accuracy and completeness of the data extracted. No assessments of the document quality were made to evaluate the potential bias or systematic error as the current study was not designed to investigate the effect of an intervention or exposure.

## 3 Results

**Figure 1:**
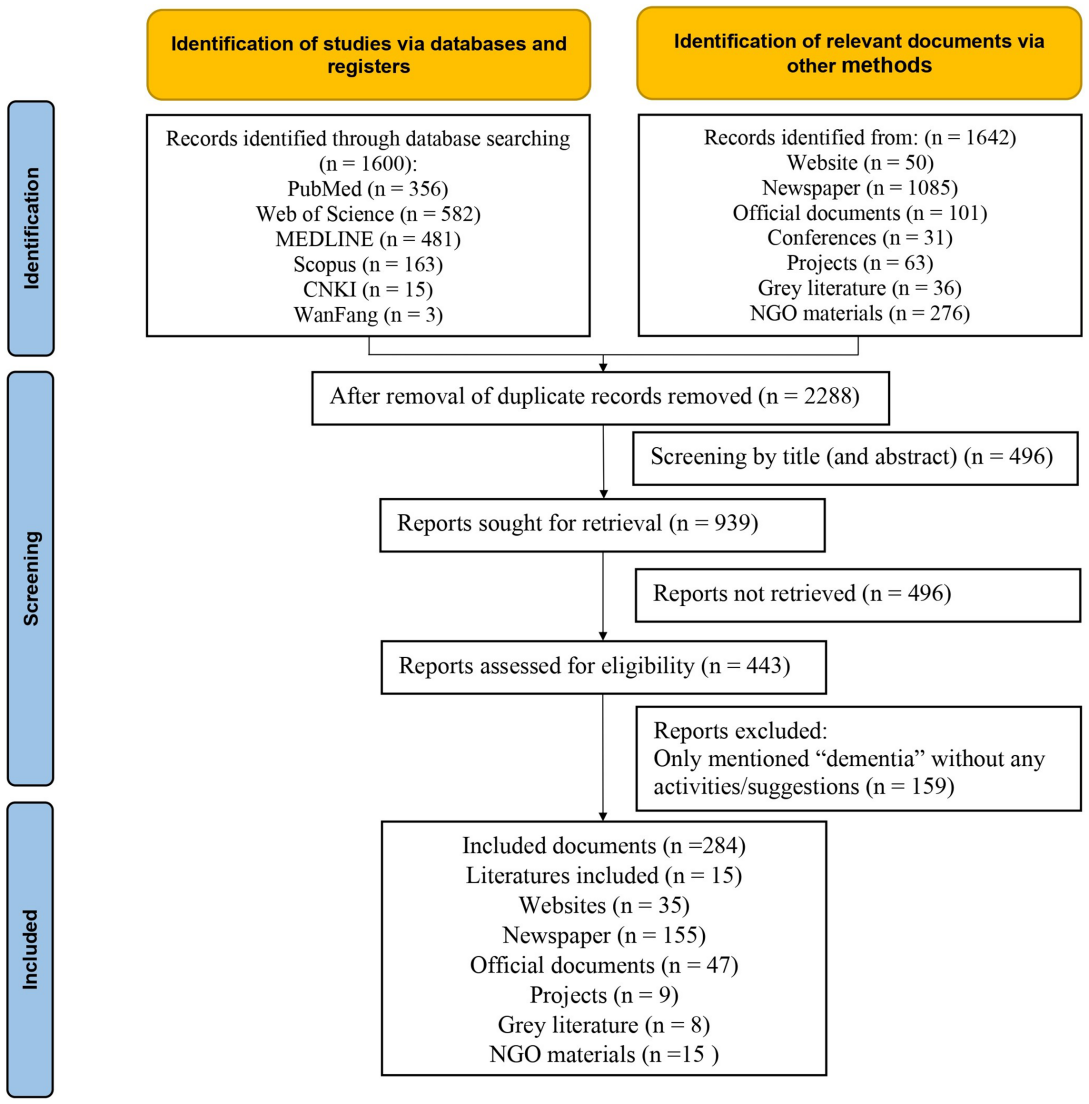
PRISMA-ScR flowchart showing selection strategy.

**Figure 2:**
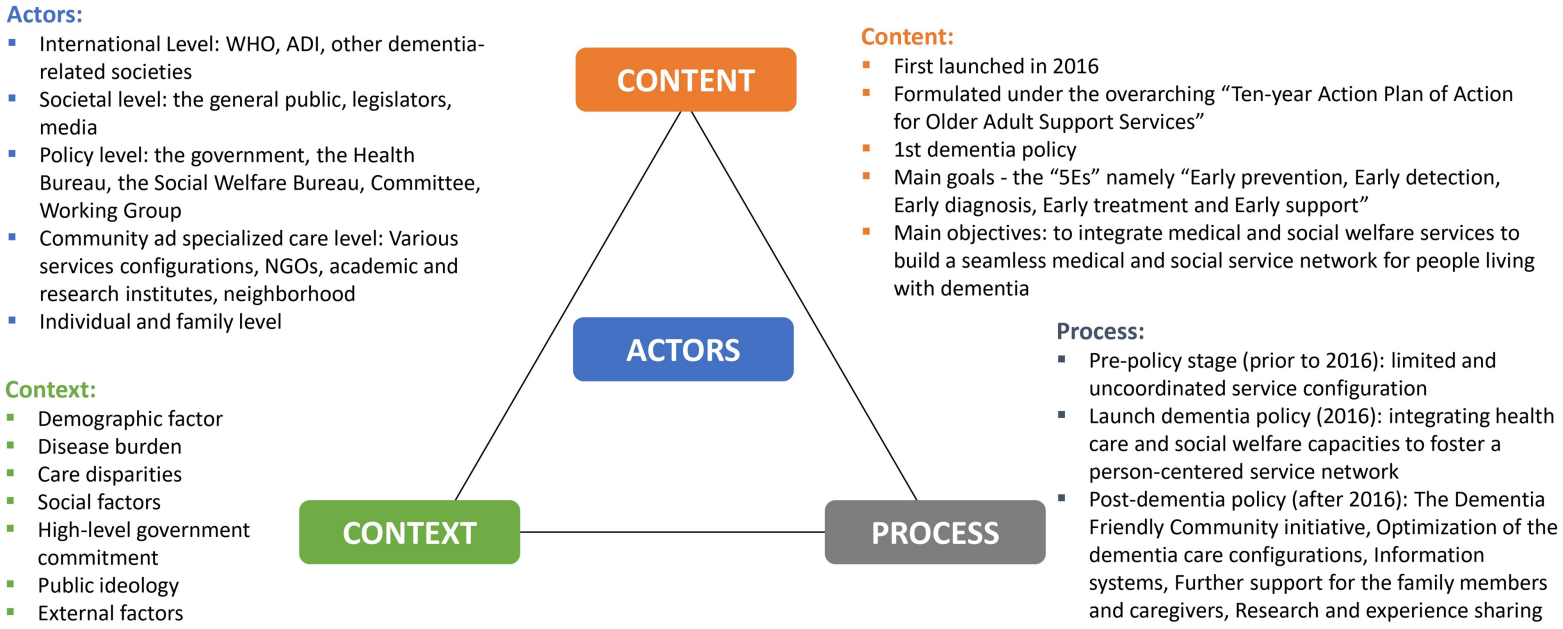
Health Policy Triangle Framework for the Macao Dementia Policy.

The search resulted in 1600 scholarly work and 1,642 pieces of other documents (including policy documents, newspapers, NGO materials, etc.), of which 2,288 remained after removing duplicate records. After the title and full-text screening based on eligibility criteria, an additional 2004 documents were eliminated. The final analysis included a total of 284 eligible documents (Figure 1). The data elements from all included documents are shown in the Supplementary material. Upon data analysis under the guidance of HPTF, the “*Content*,” the “*Actors*,” the “*Process*” and the “*Context*” involved in the developing and implementing dementia policy and the changes as a result in Macao had been identified as depicted in Figure 2.

### 3.1 Content

The Macao Dementia Policy was first launched on 21 September 2016 under the overarching “Ten-year Action Plan of Action for Older Adult Support Services” (known as the “Ten-year Action Plan” hereafter) released by the Macao government. The Ten-year Action Plan served as a roadmap for optimizing older adult care plans and service mechanisms and dementia-related goals had been integrated into the short-term (2016–2017), mid-term (2018–2020) and long-term plans (2021–2025). The key action areas included: optimization of services (e.g., cognitive assessment, specialist care, disease case management) and service configurations (e.g., memory clinic, day care, long-term care, palliative and hospice care); public education (e.g., disease awareness and prevention, life and death education); capacity building of care providers (dementia screening, multi-disciplinary care); and a tracking system and a registry system.

It was against the background of the Ten-year Action Plan that the Macao Dementia Policy was launched to integrate health and social services to build a seamless medical and social service network for people living with dementia. As shown in Table 2, the Macao Dementia Policy emphasized on five main goals (or known as the “*5Es*” namely “*Early prevention, Early detection, Early diagnosis, Early treatment and Early support*”) (34). The first 2E’s targeted the general public and “hidden” patients in the community setting to raise awareness about the disease and preventative measures, and to facilitate early detection. The latter 3E’s were designed to address the needs of people diagnosed with dementia and their caregivers subject to increasing challenges as the disease progressed.

**Table 2:** The Macao dementia service network upon the launch of the dementia policy.

Macao dementia policy framework					
Leading government agencies	The health bureau and the social welfare bureau of the Macao SAR Government				
Domains	Prevention	Detection	Diagnosis	Treatment	Support
Major goals	***E**arly prevention*	***E**arly detection*	***E**arly diagnosis*	***E**arly treatment*	***E**arly support*
Major strategies	Promotion and public education	Cognitive function evaluation	Diagnosis	Treatment, caregivers education and social services allocation	Specialized older adult care
Coordinating and executing bodies	Dementia working group Health Centres at the primary care setting in the public sector		Dementia medical centre dementia support centre		
Partnerships	Non-government organizations subsidized by the government				

### 3.2 Actors

**Figure 3:**
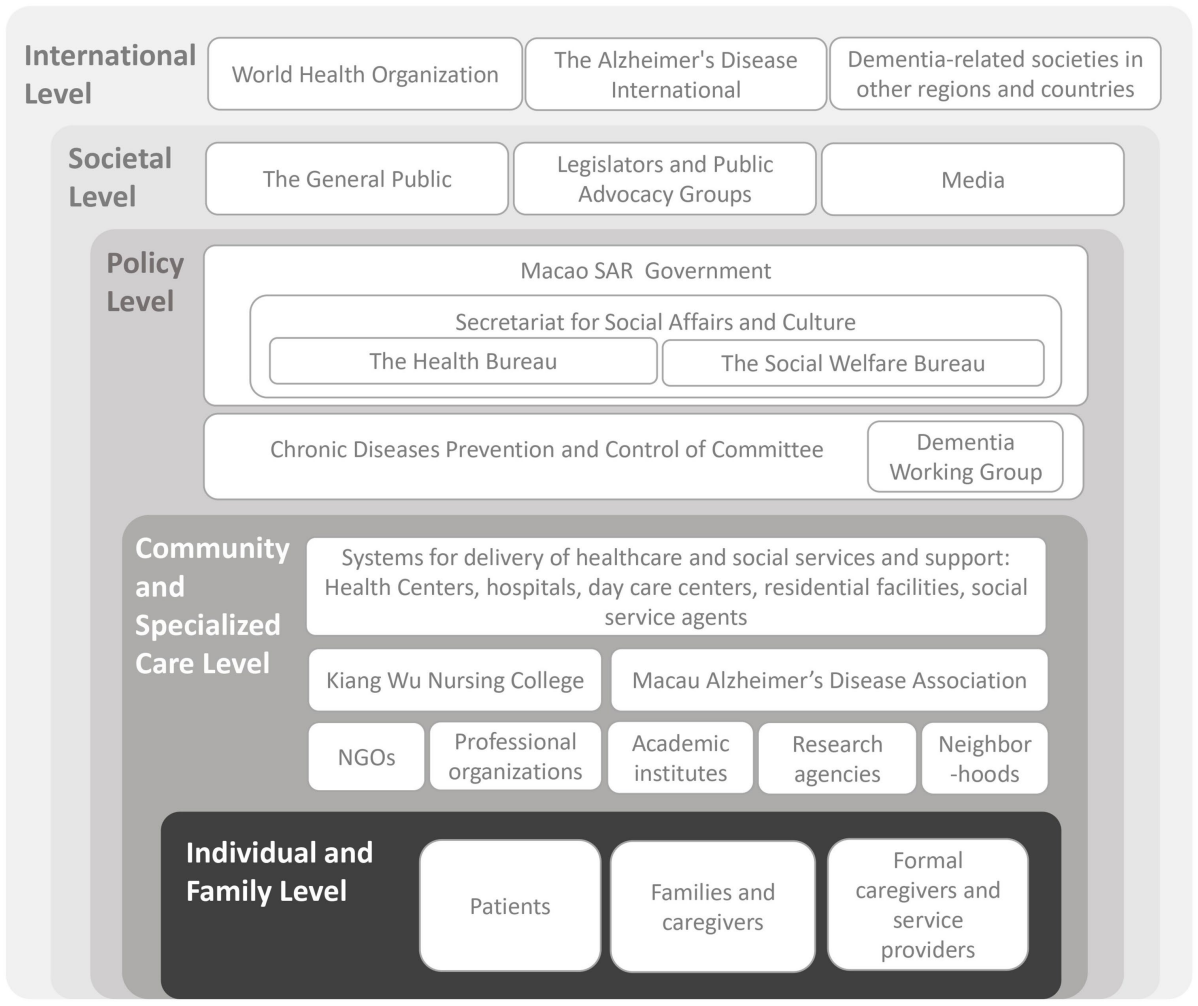
Actors at different stages of formulation, communication, implementation and evaluation of the Macao Dementia Policy.

An overview of the Macao Dementia Policy identified key actors at different stages of policy formulation, communication, implementation and evaluation at various levels: (1) Individual and family level; (2) Community and specialized care level; (3) Policy level; (4) Societal level; and (5) International level (Figure 3).

#### 3.2.1 International level

The WHO played an integral role in calling for actions to improve the lives of people with dementia, their caregivers and families, while reducing the burden of dementia on the individuals and the societies (4). Through its official relations with the WHO, the ADI empowered the local counterparts to formulate policies and strategies for better managing dementia as a health priority. Sharing of experiences with dementia-related societies in other regions and countries (e.g., the Hong Kong Alzheimer’s Disease Association and the UK Alzheimer’s Society) was also considered instrumental to the decision-making regarding the Macao Dementia Policy (35).

#### 3.2.2 Societal level

The Macao Dementia Policy adopted a multiple stakeholder approach, emphasizing the importance of leadership, coordination and shared responsibilities in implementing the patient-focused initiatives for dementia care. Concerns regarding the assurance of adequate older adult care and the necessity for action to manage dementia challenges were prominently raised by the public, legislators and public advocacy groups. Issues pertaining to disease prevention and education, social services facilities, professional education, disease diagnosis pathways, and support for families and caregivers were discussed repeatedly (36–39).

#### 3.2.3 Policy level

The Macao government established a clear hierarchy of responsibility, designating dementia as a priority and assigning the Secretariat for Social Affairs and Culture to design and formulate the policy. Operating under the Secretariat, the Health Bureau and the Social Welfare Bureau were the major implementing bodies in joint forces. To facilitate the implementation of the policy, the Dementia Working Group was formed within the Chronic Diseases Prevention and Control of Committee. This group was tasked with communicating about and coordinating actions among multisectoral actors.

#### 3.2.4 Community level

Various entities were involved in the provision of dementia care at the community level including health institutions (public and non-public), NGOs and academic institutes. Within the public sector, older individuals could assess medical care at the primary health centers and, whenever necessary, be referred to specialist at the public hospital. The public hospital also established a memory clinic and conducted dementia seminars to promote multidisciplinary approach for the diagnosis and treatment of dementia. Some NGOs, especially those subsidized by the government, played a great role in providing day care, home care, rehabilitation, long-term care, and residential care services for dementia patients deemed eligible by the Health Bureau (40–42). Specialized geriatrics clinic, memory clinic, geriatrics care unit and multidisciplinary medical team for dementia patients started to take form in the private sector.

A significant part of dementia care work also originated from dementia-related NGO such as the Macau Alzheimer’s Disease Association (MADA) (43). Established in 2010, MADA focuses on fostering public awareness and positive attitude toward dementia. They offer training programs for caregivers and professionals and promoting institutional collaboration and service networks. MADA became a member of ADI in 2013, serving as a bridge between local and international counterparts (44). Other NGOs were also actively involved in public education and campaigns to enhance public awareness about dementia (42).

Academic institutes such as the Kiang Wu Nursing College of Macau (KWNC), also played an important role in dementia care (42). KWNC initiated the ‘Benevolence Lights up my Later Life’ project in 2011 (45, 46). This project was designed to focus on public education (including dementia prevention and care, disease management, hospice care, and care in community and residential setting), scholar training for professionals (involving online training, dementia care courses, workshops and field trips), as well as community services and research (such as dementia service hotline, chronic disease management, cognitive ability assessment). Local research agencies were also active in conducting research about dementia.

#### 3.2.5 Individual and family level

The needs of the patients and their families and caregivers, and the challenges encountered by the service providers remained the key drivers in developing and optimizing the person-centered care model for dementia.

### 3.3 Context

The context of the dementia policy refers to a set of social, political and economic processes and structures which shape the policy system in different ways (47). Understanding the context is crucial for comprehending how policies are formulated and implemented. Based on the findings, several contextual factors have been identified that could impact the implementation of the Macao Dementia Policy.

#### 3.3.1 Demographic factor

Macao is experiencing a demographic shift toward becoming a “super aged society.” The average life expectancy of Macao citizens was 83.4 years in 2017, and had reached 84.2 years in 2021 (34, 48, 49). Senior citizens aged 65 or above accounted for 10.5% of the total population and are projected to reach 20.7% by 2036. According to a local study, the adjusted dementia prevalence among the population aged 60 and above in 2019 was 4.94% and the incidence was 10.6%. This translates to more than 6,000 people with dementia and more than 1,000 new cases each year in Macao (19).

#### 3.3.2 Disease burden

The increasing prevalence of dementia is inevitably becoming a major cause of disability and dependency among older people, posing significant health and social burden on the society. According to the Dementia Registry in Macao, among the 2,623 registrants, 294 (11.2%) lived alone, 338 (12.9%) lived in nursing home, and 1991 (75.9%) lived with family (50). The need to strengthen home-based care, support for family and caregiver and long-term care facilities is mounting.

#### 3.3.3 Care disparities

The underdiagnosis of dementia in the city remained a huge challenge for ensuring access to adequate care and support for patients, their families, and caregivers. Reportedly, the diagnosis rate of dementia in Macao was 37.5% in 2015 (49, 51), which was lower than the expected diagnosis rate of 50% set by the World Health Organization (4), Due to delay in diagnosis and management, affected individuals were bound to suffer from a worse prognosis and poorer quality of life. Nevertheless, the diagnosis rate of dementia had increased since the implementation of the Macao Dementia Policy to 51.5% in 2018 (34).

#### 3.3.4 Social factors

The level of social awareness, understanding and acceptance of dementia significantly contributed to its underdiagnosis. It was found that dementia literacy was generally inadequate in Macao (52). Part, if not most, of the society considered dementia as a natural part of aging (53). Due to the sensitivity and stigma associated with dementia, patients and families tended to ignore the symptoms and avoid disclosing them to others (34). Furthermore, younger generations are increasingly less likely to attend to the needs of older persons especially people with dementia, demonstrating a weakening trend of family support (34).

#### 3.3.5 High-level government commitment

To cope with the opportunities and challenges brought by an aging population and to enhance policies and services for the older adult to boost their well-being, the government formulated the Ten Year Action Plan, which includes dementia as a key area of action (54). The main objectives of the Ten Year Action Plan are categorized into four major areas: ‘medical services’, ‘rights protection’, ‘social participation’ and ‘living environment’, comprising of 445 short-, medium-and long-term measures. The overall goal is to build an inclusive society where the older adult are provided for, have a sense of belonging, and have the opportunity to contribute to demonstrate their value. This overarching policy serves as a cornerstone for optimizing various services for people living with dementia.

#### 3.3.6 Public ideology

Public opinion continued to call for the strengthening of dementia management (55). The day care facilities for older adult with dementia were too few to address the actual needs (56). Training opportunities to the patients and to their families on cognitive functions and daily living ability were also not enough (57). The long waiting time for residential care services added on the distress for the family. The caregivers’ physical and mental pressure also warranted close attention (58). Furthermore, it was found that the medical services and social care were fragmented and a lack of awareness of dementia among health professionals remained (59). Overall, there was a consensus across the society that the demand for institutions providing specialized dementia services in Macao exceeded the supply, and a comprehensive strategic approach covering public education, early identification and treatment, medical and social services, and support for caregivers was very much in need (58, 60, 61).

#### 3.3.7 External factors

International organizations such as the WHO had great impact on driving the development of the health sector in Macao all along. For instance, the primary healthcare system established in 1985 to offer Macao residents easy access to primary healthcare services in their own neighborhoods was to realize the objective of Health for all 2000 advocated by WHO (62). Similarly, the formation of the Macao Dementia Policy was also in alignment with the WHO’s recognition of dementia as s a public health priority in 2012 (63), and the *World Health Organization Global Action Plan on the Public Health Response to Dementia 2017–2025*.

In addition, the Alzheimer’s Disease International also played a major role in guiding the development of dementia care and practices in the city. The Macao Dementia Policy was recognized by the ADI as the 27th globally and one of the highest stage 5 to develop dementia friendly community and primary health professionals (34). One of the major local dementia organization, MADA, was founded in 2010 and had been a member of ADI since 2013 which played the bridging role between the local and the international counterparts (44).KWNC was among the first to be accredited by the ADI to have the standards of their training and learning activities recognized by the ADI (64).

### 3.4 Process

**Figure 4:**
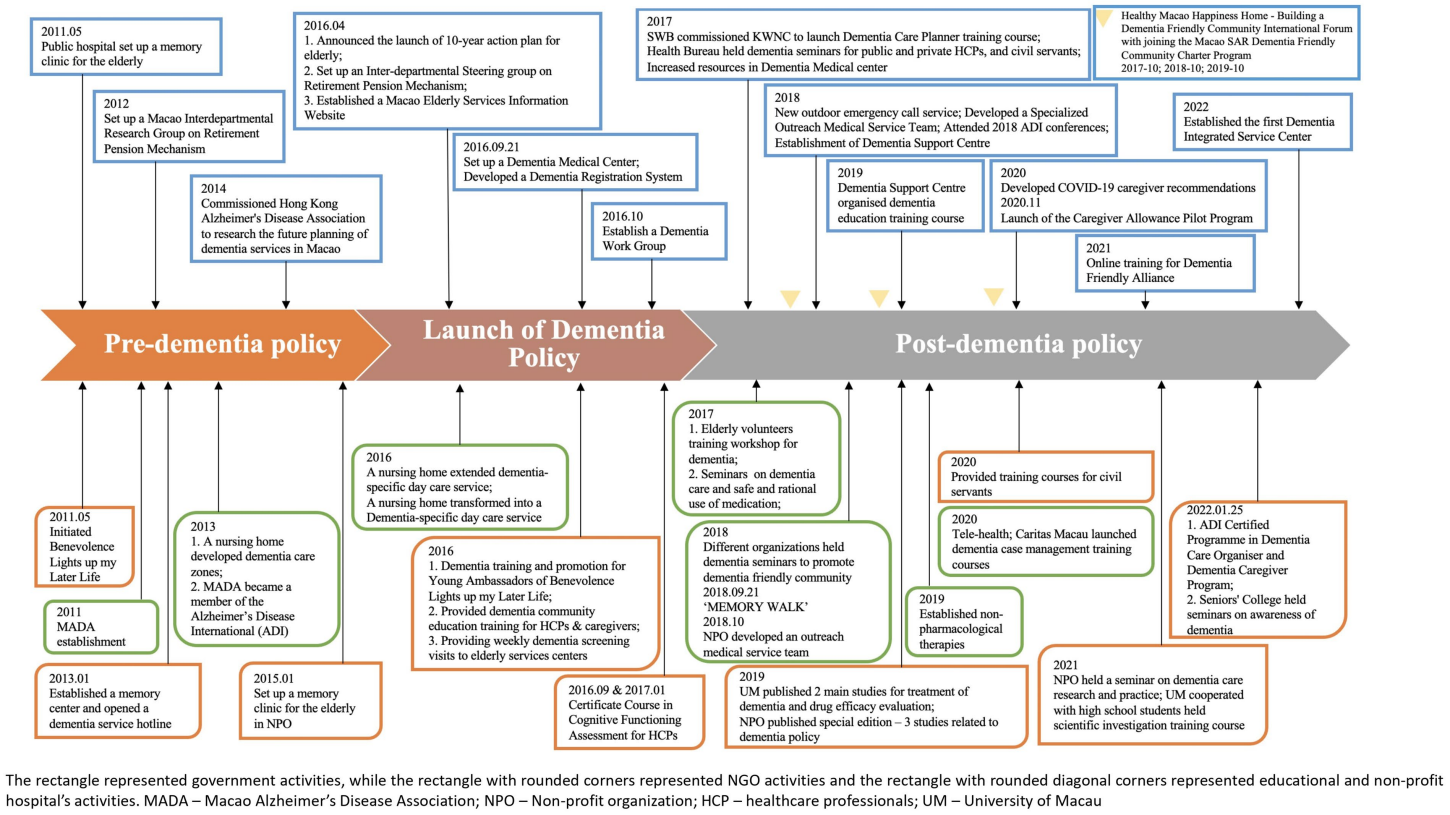
An overview of the implementation procedure for the Macao Dementia Policy.

As shown in Figure 4, the implementation process could be roughly classified into the three stages (as shown in Figure 2): pre-dementia policy initiatives (prior to 2016), launching dementia policy (2016), and post-dementia policy (after 2016).

#### 3.4.1 Pre-policy stage (prior to 2016)

Prior to 2016, the medical and social support services were already in place to set a foundation for developing integrated services mandated in the dementia policy (Figure 4). According to the Act of 24/86/M, Macao residents aged 65 and over are eligible for free primary and secondary medical care in the public sector (65). The majority of older individuals often sought medical care at the primary health clinics and, whenever specialist care was deemed necessary, were referred to secondary care at the public hospital. Additionally, the government introduced the Medical Subsidy Program in 2009, which annually provided health care vouchers to Macao residents, supporting them to receive medical care in the private sector (66). This scheme was set to lessen the burden on the public sector and increase coordination between the public and private healthcare sectors (67). Besides, the Macau government was also committed to promote community-based and home-based care through operating older adult day centers and subsidizing home care and other related supporting services (18).

Although the services for the older adult were expanding, there was no initiative specifically designed for dementia management initially. This was until the society expressed concerns about a lack of public awareness regarding dementia prevention and insufficient healthcare and social support targeted the disease resulting in delayed identification and diagnosis (68). One of the first responses from the society was the establishment of MADA in 2010 to promote dementia-related development in Macao which later became a member of ADI in 2013 (43). In May 2011, the government took the initiative to establish a memory clinic and develop a series of dementia seminars at the public hospital to promote interdepartmental diagnosis and treatment for dementia patients and increase public awareness (69–74). Apart from the public hospital, the public primary health centers also developed mental health department for enhancing early diagnosis of dementia in the community (75). Such actions marked the beginning of systematically publicizing the significance of dementia prevention.

Non-profit organizations such as KWNC also launched a memory center and a dementia telephone service hotline to raise awareness among the community, the older adult, caregivers, and frontline service personnel, thereby improving the early identification of dementia and the provision of assistance (76). Noteworthy, the government also cooperated with the college to implement the *Benevolence Lights up my Later Life Project* to increase public awareness of dementia, which featured ongoing community promotion of dementia through public seminars, and caregiver and healthcare professional training (77). Other non-governmental organizations continued to act proactively in the community by building dementia-specific service areas, and providing dementia-specific training and care services (78), and the establishment of a dementia care room for the older adult with dementia (79).

Despite the active participation of multiple sectors to respond dementia management, specific programs attended to dementia patients and their caregivers were limited. Concerns were raised about the fast-growing older adult population and increasing life expectancy, prompting the government to research about the international policies and theories on caring for the older adult in order to formulate a well-informed policy for dementia (80). Public consultations were conducted and public opinions were collected and analyzed in 2014 and 2015 to obtain initial policy information (81). The findings highlighted the scarcity of dementia support services, resulting in pressure on caregivers. Day care centers provided mixed care for dementia patients and no specific accommodations were in place that addressed their pathological needs, presenting great challenges in providing adequate dementia services (81).

The government proposed a partnership with non-governmental organizations in order to facilitate health promotion and education initiatives in the community and to expand professional health services (68). However, the lack of coordination between the healthcare services and community support, as well as the decentralization of community services, led to confusion for the older adult not knowing what support was available and how and where to seek such support. The gaps at the interface between the health sector and the social service sector care with the society presented a much-needed macro approach of coordination. Preliminary research about dementia in community-dwelling older adult and the need to develop a memory clinic and the services needed by older adult was conducted (45).

#### 3.4.2 Launch of dementia policy (2016)

The launch of the Macao Dementia Policy by the government on 21 September 2016 was accompanied by a series of actions and programs with the intention of establishing a dementia service network through interdepartmental and interprofessional collaboration (23). In particular, the priorities of action included: (1) to increase public awareness and understanding of dementia through public education in collaboration with NGOs; (2) streamlining cognitive assessment and referral process; and (3) strengthening integrated medical services and social care.

To coordinate efforts from different sectors, the government established the Dementia Working Group involving stakeholders from community organizations to work together for planning and executing programs that promote preventative education in the community, raise public awareness of dementia, and encourage cognitive evaluation for early prevention and early treatment. In order to achieve the goals of the Macao Dementia Policy, specialized working groups had been established such as the Dementia Medical Center and the Dementia Support Center (82). The Dementia Support Center was established in Dec 2018 to provide patients and caregivers with more comprehensive support services, such as education and training classes, cognitive stimulation therapy, and remembrance therapy.

The Dementia Diagnosis and Treatment Center (DDTC), which was jointly established by the Health Bureau and the Social Welfare Bureau, was set to integrate the resources of primary health centers, the public hospital and the social service to become an one-stop focal point for diagnosis, treatment and social service support (83). To reduce the waiting time for consultation and ensure the continuity of patient care, a green-channel dual-referral mechanism was devised (84). People who showed signs of cognitive dysfunction in the Health Centres would be referred to the DDTC for further evaluation, diagnosis and treatment. Upon confirmed diagnosis, patients in need would be provided with integrated medical service and social care services including home health care services, day care centers and nursing home would be provided by subsidized NGOs or care facilities based on a standardized assessment mechanism and a centralized referral system. At the same time, patients who were stabilized upon treatment would be referred back to the primary health center for continuous monitoring.

To strengthen the public-private partnership to promote a dementia-friendly community, more than 160 groups and institutions from social services, youth, schools and related professional fields were invited by the government to jointly form the Dementia-Friendly Community Alliance (85). The Alliance advocated for building dementia-friendly communities and raising awareness through volunteer and caregiver trainings, seminars, workshops, movies, and brochures (85).

To kick start the action on improving early diagnosis, the cognitive assessment network was extended to all eight Health Centers and to some private health settings. With Nearly 100 medical staff members from the Health Bureau and community were trained to competently perform cognitive assessment. Individuals suspected of dementia could make an appointment for cognitive function evaluation at any of the service points in the community. Academic institutes had been dedicated to the training of professionals, patients and caregivers, and the general public (64).

#### 3.4.3 Post-dementia policy (after 2016)

A number of strategies had been carried out to implement The Macao Dementia Policy to the fullness its full extent such as The Dementia Friendly Community initiative, Optimization of the dementia care configurations, Better Support for the Family Members and Caregivers, Fostering the Information System, Research and Scholar Sharing of Experiences, among others.

##### 3.4.3.1 The dementia friendly community initiative

The Dementia Friendly Community initiative emphasized on 5 main elements: “*Friendly Government*,” “*Friendly Organization*,” “*Friendly Environment*,” “*Friendly Caregiver*,” and “*Friendly Citizen*,” to which actions were taken to achieve the goals.

###### 3.4.3.1.1 Friendly government

The working mechanism run by the Dementia Working Group for coordinating dementia-related in Macao continued to leverage on government leadership, multi-departmental cooperation, and whole-society participation, giving priority to dementia management, health promotion and public education, early screening for high-risk groups, and empowering group under the impact of dementia. In 2018, “The Legal System for Protecting the Rights and Interests of the Older adult” was established to promote the construction of an inclusive society in which the older adult would be provided with support, belonging and fulfilling (86). The bill emphasized that the older adult should be protected in terms of support and care, health, basic living security, housing needs, occupation and work, accessibility, preferential treatment and preferential rights, adding emergency judicial assistance to the older adult whenever necessary. Such legislation called for respect for older adult’s rights and interests at a higher level (86).

###### 3.4.3.1.2 Friendly organization

One of the first initiatives to consolidate the public-private partnerships was the “Macao Dementia Friendly Community Charter” programme co-organized by the Health Bureau, the Social Welfare Bureau and MADA. As of 2023, 164 groups including social service agencies, higher education institutions, related professional groups, schools, as well as patient organizations serving the older adult, families, youth and communities had joined forces to build a dementia friendly community.

###### 3.4.3.1.3 Friendly environment

Every year, the Dementia Working Group took the opportunity of the World Dementia Month designated by the ADI to hold community-wide campaigns to raise public awareness and attention to dementia. For instance, in response to the publicity work of World Dementia Month on the theme “*Know Dementia, Know Alzheimer’s Disease*” in 2022, the Dementia Working Group organized the Macao Dementia Friendly Community online quiz game with prizes to help the public gain a better understanding of dementia and the dementia service network in place. Different media such as newspapers and television were frequently used to introduce dementia information and the work of the Dementia Working Group. Youth ambassadors and senior ambassadors had been recruited to promote the concept of dementia friendly community.

###### 3.4.3.1.4 Friendly caregiver

The government provided ongoing trainings for long-term care personnel and caregivers to optimize dementia care, and allocated additional resources for NGOs to provide better care for older adult with dementia. In-person education at the community senior centers or workshops for dementia patients and their family member and caregivers. Special training for healthcare professionals, social welfare professionals, other care providers, public servants (e.g., policy officers) and other community workers (bus drivers, security guards in residential buildings) (87–89). To enhance dementia professionalism and knowledge, special courses for healthcare professionals and caregivers had been designed, including the Dementia Care Manager and Dementia Caregiver Training programs certified by the ADI (90, 91). There was a cooperation among various NGOs to provide public seminars and workshops on senile dementia prevention and care, thereby enhancing their service networks and integrating of community resources (92).

###### 3.4.3.1.5 Friendly citizen

Disseminating community-based promotion, training, support programs, and seminars remained a priority for fostering dementia friendliness, early diagnosis, help-seeking behavior and disease prevention among the general public. Education materials of dementia and dementia care were widely available and there was even a Dementia Information Page set up on the Health Bureau mobile app and website to allow the public access to dementia information online at any time. Micro-movie and video production about dementia prevention were also used as tools for disease promotion. Dementia training programmes were designed specifically to the to cater to minorities such as the fishermen during the fishing moratorium training program.

##### 3.4.3.2 Optimization of the dementia care configurations

To achieve early diagnosis, the cognitive function assessment network continued to expand and took place in different settings simultaneously. On one hand, the government took the lead to extend the cognitive function assessment across the primary Health Centres throughout Macao. Trained medical staff from the Health Bureau and the community were positioned to perform cognitive function assessment to see the number of diagnosis increased from 1,603 in 2016 to 2,290 in 2018, and the dementia diagnosis rate increased from 37.5% in 2015 to 51.5% in 2018 (48). Based on the streamlined referral process mentioned above, the waiting time for consultation at the Dementia Medical Center was greatly reduced from more than 6 months to less than 1 month (34).

The DDTC provides services such as cognitive function assessment, diagnosis, treatment, medication guidance, emotional counseling and social services referral to patients with dementia around 2000 person-times a year. It also plays a crucial role in offering initial diagnosis and ongoing treatment to manage dementia effectively. To provide more targeted, comprehensive post-diagnosis services, the Dementia Support Center (DSC) was established in 2019. The DSC provides case consultation, education and training to professionals, non-pharmacological treatment, other support services to the patients and their caregiver, and group health education for the general public. The DSC continued to provide post-diagnosis support services to patients with dementia and their families, including education, cognitive training activities and non-drug treatments serving around 400 person-times a year (93). The DSC in particularly focused on providing post-diagnosis support services such as cognitive function assessment, examination, diagnosis and treatment as well as nursing consultation to patients in need.

After receiving a diagnosis from the DDTC, dementia patients and their caregivers can be referred to the DSC for follow-up social services. With the DSC working seamlessly with the DDTC, a Dementia Health and Social Services Network had been built to help patients maintain physical functions, slow disease progressions, enhance the caregivers’ ability and confidence, and reduce caregivers’ burden, maintaining the quality of life of patients and caregivers (94).

In addition to those efforts, NGOs have also initiated cognitive function screening programmes in the community senior centres. People with “normal cognitive function” would be followed up yearly and provided with education materials. People with “mild cognitive impairment” would be provided with cognitive training or employee training. People with “obvious impairment” would be referred to further assessment or home visit. Dementia Service hotline was also set up to help people in need to navigate the service systems for dementia.

Moreover, dementia patients and their caregivers might be entitled to further medical and social services through the Home Care Support program, Patient Support Center, and Palliative Care and Hospice Care Team (95). To meet social needs, more older adult integrated service centers, day care centers, and nursing homes have been established (96, 97), and non-pharmacotherapy such as reminiscence therapy and cognitive stimulation therapy (98) has been gradually introduced in pre-existing dementia facilities to enhance patients’ quality of life and delay dementia progression. Additionally, with the support from the government, a NGO built a mobile emergency call service for outdoor location and emergency support for dementia patients (99).

To provide 24-h accommodation, care, and training for patients and caregivers, the Dementia Integrated Service Center (DISC) was established in 2022 (100). The DISC provided standardized assessment, referral, and waiting mechanism matching the older adult with appropriate support services based on their conditions and the availability of public resources.

Special geriatric care programme with special focus on dementia was also in place in the hospital setting. This included geriatrics department, a memory clinic, and geriatrics doctors to meet Macao’s aging population. The hospital developed a multidisciplinary team of rehabilitation therapists, pharmacists, and Chinese medical doctors to provide integrated care. A community outreach team was also created to improve patients’ quality of life and reduce the burden on caregivers. Moreover, Macao’s only palliative care center established in 2000 advocated for the Do Not CPR (DNCPR) for patients who refuse to perform CPR in emergencies (101). The hospital upholds ethical principles of non-harm, avoiding ineffective treatment, maintaining the best interests of patients, and respecting their autonomy.

##### 3.4.3.3 Information systems

In order to gain a better understanding about the situation of patients with dementia in a timely manner, the Dementia Registry System was set to acquiring pertinent scientific data about how the disease progressed and how the patients were managed over time. The data collected would be very important to inform policy formation, patient management, and follow-up services.

##### 3.4.3.4 Further support for the family members and caregivers

The government launched the Caregivers’ Allowance Scheme to support low-income dementia caregivers (102). Accordingly, family members and caregivers of dementia patients who were in need could apply for financial assistance or related subsidies from the Social Welfare Bureau if deemed eligible. On the other hand, The Macao SAR Universal Design Architectural Guidelines for Accessibility and Senior Home Safety Assessment and Equipment Grant Program have been developed to promote home safety awareness for inclusivity (103, 104).

##### 3.4.3.5 Research and experience sharing

Research efforts in Macao pertaining to dementia encompassed areas like epidemiology and economics, dementia disease mechanisms and models, dementia diagnosis, drug development, and dementia care and support (A list of scientific publications conducted about dementia by research agencies in Macao is provided in Supplementary File). While the majority of dementia research related to R&D (including the discovery of new pharmacological targets and novel treatments, as well as the potential application of TCM in the treatment of dementia), some studies focused on medical and societal demands (e.g., investigating age-related differences in dementia literacy and the need to improve early detection and diagnosis). A study found that the public promotion in place were helpful in increasing the public’s knowledge about dementia and suggested more efforts could be diverted to increasing public awareness of dementia risk factors (105). In addition, participating stakeholders were also keen on reporting the experiences in Macao at international conferences such as the Dementia International Forum and sharing experiences with experts from other nations (106).

##### 3.4.3.6 Special attention to dementia during COVID-19

The Health Bureau formulated the “*Recommendations for Caregivers/Friendly Groups of Home-Based Patients with Mild to Moderate Dementia During the COVID-19 pandemic*” to call for attention and support*. The older adult service facilities of the Dementia Friendly Community Alliance showed great support and cooperation, and actively integrated the recommendations into their own anti-epidemic measures and contingency plans when arranging staff deployment and accommodation during the epidemic, and adopting teleservices such as telephone calls, social platforms, online videos, etc. to maintain daily operation and communications.

## 4 Discussion

Using a scoping review guided by the HPTF, the study systematically analyzed in great depth the policy background (*Context*), the influences on the policy (*Actors*), the key aspects of the policy (*Content*), and the strategic approach (*Process*) of achieving significant advancement in the dementia care model in Macao. A number of key factors that might lead to successful implementation of the policy has been identified: adopting a strategic focus on “*Early Detection*” and “*Early Diagnosis*” as the priorities among the 5-Early goals by shaping a dementia-friendly community and educating about dementia across all walks of life; integrating health services and social welfare services to achieve a person-centered service model; facilitating effective coordination and partnership between the government and advocacy groups and organizations in the community for the policy filtration; and simultaneously upgrading the service configuration to manage the foreseeable challenges of escalating prevalence and severity of the disease.

The launch of the Macao Dementia Policy was a milestone for dementia care in Macao. Drastic actions have been taken to articulate a public health approach toward the challenges of dementia. The policy was driven by the overarching policy principle of providing better care for the older adult, and followed by a comprehensive approach to harness the synergy of direct public resources and partnerships with advocacy groups organizations, public health agencies, and local communities. It is evident from this study that Macao had taken a comprehensive and multi-faceted approach to underscore its commitment to the 7 key action areas recommended in the *WHO Global Action Plan on the Public Health Response to Dementia 2017–2025* (Table 3) (4).

**Table 3:** Macao Dementia Policy in the context of the WHO global action plan.

WHO global action plan		Macao dementia policy
Key action areas	Global targets	Actions taken
Dementia as a public health priority	75% of countries will have developed or updated **national policies, strategies, plans or frameworks for dementia**, either stand-alone or integrated into other policies/plans, by 2025	Macao dementia policy was implemented on 21 Sep 2016 to achieve the 5-Early goals: “***E**arly prevention, **E**arly detection, **E**arly diagnosis, **E**arly treatment and **E**arly support*” Strategies and action plans have been initiated since then
Dementia awareness and friendliness	100% of countries will have at least one **functioning public awareness campaign** on dementia to foster a dementia inclusive society by 2025 50% of countries will have at least one **dementia friendly initiative** to foster a dementia-inclusive society by 2025	The **dementia working group** was formed within the Chronic Diseases Prevention and Control of Committee and charged with the responsibilities of coordinating the execution of the Macao Dementia Policy among multisectoral actors The **dementia friendly community initiative** provided a blueprint towards “*Friendly Government*,” “*Friendly Organization*,” “*Friendly Environment*,” “*Friendly Caregiver*,” and “*Friendly Citizen*” The **dementia-friendly community alliance** was formed with the participation of 164 non-governmental organizations and groups to execute the “*Macao Dementia Friendly Community Chart*er” Every year, the Dementia Working Group took the opportunity of the World Dementia Month designated by the ADI to hold **community-wide campaigns** to raise public awareness and attention to dementia
Dementia risk reduction	The relevant global targets defined in the Global action plan for **prevention and control of noncommunicable diseases** 2013–2020	The Healthy Cities Committee and the Chronic Diseases Prevention and Control of Committee jointly **promote healthy lifestyles to reduce the risk of chronic non-infectious diseases**. The policy has encouraged healthy aging through regular exercise and a balanced diet, and has promoted the creation of dementia-friendly environments.
Dementia diagnosis, treatment, care and support	In at least 50% of countries, as a minimum, **50% of the estimated number of people with dementia are diagnosed** by 2025.	The **cognitive assessment network** has been extended to all Health Centers and to some private health settings **The dementia diagnosis and treatment center (DDTC),** which was jointly established by the Health Bureau and the Social Welfare Bureau, was set to integrate the resources of primary health centers, the public hospital and the social service to become an one-stop focal point for diagnosis, treatment and social service support The **dementia support center (DSC)** was established in 2019 to provide case consultation, education and training to professionals, non-pharmacological treatment, and other support services to the patients and their caregiver, and group health education for the general public A Dual Referral Mechanism was devised between the **cognitive assessment network and the DSC to** reduce the waiting time for consultation and ensure the continuity of patient care The **dementia integrated service center (DISC)** provided standardized assessment, referral, and waiting mechanism matching the older adult with appropriate support services based on their conditions and the availability of public resources The **home care support program**, **patient support center**, and **palliative care and hospice care team** to provide further medical and social services The **specialist outreach service programme** to provide medical outreach services at the nursing homes based on the collaboration with the community health care sectors A **mobile emergency call service** was in operation for outdoor location and emergency support for dementia patients
Support for dementia carers	75% of countries provide **support and training programmes for carers and families** of people with dementia by 2025	**Ongoing trainings** had bene provided for long-term care personnel and caregivers to optimize dementia care The government launched the **caregivers’ allowance scheme** to support low-income dementia caregivers The **universal design architectural guidelines for accessibility and senior home safety assessment and equipment grant program** had been developed to promote home safety awareness for the older adult and inclusivity **Additional resources** were allocated for NGOs to provide better care for older adult with dementia which in turn help alleviate the burden on the carers The Social Welfare Bureau and **private aged services organizations** collaborate closely to provide home care and support service teams, home cultivation services, day care facilities, and nursing homes. **Day care centers** provided case counseling, emotional support, care training, the loan of assistance devices, and other services for caregivers. NGOs provided caregiver support services specifically for dementia caregivers.
Information systems for dementia	50% of countries routinely **collect a core set of dementia indicators through their national health and social information systems** every 2 years by 2025	The **dementia information website** was set up under the official website of the Health Bureau in order to inform the public about dementia The **dementia registry system** was set to acquiring pertinent scientific data about how the disease progressed and how the patients were managed over time.
Dementia research and innovation	The **output of global research on dementia doubles** between 2017 and 2025	Dementia **research related to R&D** (including the discovery of new pharmacological targets and novel treatments, as well as the role of Traditional Chinese Medicine), and **research about medical and societal demands** (including age-related differences in dementia literacy and the need to improve early detection and diagnosis) had been ongoing

Our findings align with prior research emphasizing that highlight the significance of reevaluating national dementia policies. Chow et al. conducted a thorough review of National Dementia Strategies implemented in various countries, examining successful strategies encompassing policy frameworks, resource allocation, community support, and public education (107). These findings offer valuable insights and recommendations to bolster Canada’s dementia management and support infrastructure. Similarly, Collins et al. conducted an in-depth analysis of England’s national and local dementia strategies to offer insights into effective primary prevention measures for dementia and to inform future policy development and implementation endeavors (27).

### 4.1 Fostering the dementia-friendly community for social inclusion

The concept of dementia-friendly community originated from the Age-Friendly Cities initiative of the World Health Organization (108). An inclusive dementia-friendly community refers to a place where dementia patients can be understood, respected, supported, and empowered to remain confident about their own values to the community (109). It has been shown that dementia-friendly communities could contribute to good health for people under the impact of dementia (110, 111). Our findings showed that such endeavor in Macao were in alignment with the principles promoted by the ADI (112) in which people with dementia, communities with supportive physical and social environments, dementia-friendly organizations and groups, and partnerships among governments, service agencies and NGOs were all engaged.

However, people living under the impact of dementia, either the patients or their informal caregivers, may face continuous challenges of stigma, social exclusion, and difficulty in navigating and assessing local support resources. One important gap could be the insufficient involvement of younger caregivers, individuals from different backgrounds and the perspective of individuals with more various severity of dementia symptoms to take into account their needs and experiences (113). For this, better understanding of young informal caregivers’ perspectives, involving them in intergenerational activities, raising awareness and education across all societal sectors, are crucial for creating future inclusive dementia-friendly generations (114). Innovative strategies and technologies to engage and include people with dementia in their community activities as we understand from our experiences of the COVID-19 pandemic are also highly significant to promote dementia friendly communities for social inclusion (113).

### 4.2 Expanding the “risk reduction” in “early prevention”

There is abundant evidence that noncommunicable diseases (such as diabetes mellitus and midlife hypertension) and lifestyle-related risk factors (such as physical inactivity, obesity, unbalanced diets, tobacco use, harmful use of alcohol) play a role in dementia (115, 116). Other potentially modifiable risk factors reportedly related to dementia include social isolation, cognitive inactivity and mid-life depression (117, 118). The Lancet Commission on dementia, prevention, intervention and care indicated in 2020 that as many as 40% of dementia cases could be prevented or delayed by addressing modifiable risk factors. “Dementia risk reduction” was one of the key action areas in the WHO Global Action Plan and efforts to addressing modifiable risk factors for dementia should start from childhood and extend throughout life (4). This way, individuals and populations can be empowered to make healthier choices and follow healthier lifestyle patterns that will also benefit their overall health.

Subsequent strategies developed under the Macao Dementia Policy should shift more focus on “Early Prevention” and position the risk reduction and prevention goal as the new “best practice” for dementia management (119). For instance, in China (120) and the UK (121, 122), public awareness of dementia and risk reduction prevention has become one of the overarching goals in the national plan for dementia. As suggested by the WHO, actions to promote risk reduction for dementia can be two-fold: (1) The “Dementia Risk Reduction” goal can be linked to other public health or health promotion programmes, policies and campaigns on risk reduction of other noncommunicable disease or health protective measures; (2) Support the health and social care professionals to build their capacity in providing evidence-based, gender and culturally appropriate interventions to the general population about and proactively manage modifiable risk factors for dementia.

### 4.3 Preparedness for autonomy support and end of life care

Dementia is a life-changing disease that progresses over time. The range of health and social care in place, whether it be community support or long-term-care services, should adequately speak for their changing needs (109, 123). As recommended by the WHO, people with dementia should be empowered to live in the community and looked after according to their needs and preferences to maintain a level of functional ability consistent with their basic rights, fundamental freedoms and human dignity (4). The capacity to provide integrated, person-centered, accessible, affordable health and social care in a timely manner at the community level counts of the dementia-friendly community. When patients progress to high levels of dependency and morbidity in the later stages of dementia, a more complicated mix of care will be needed. Preparedness for long-term care capacity particularly in the area of palliative/end-of-life care is not only a core component of the continuum of care for the patient but also important physical, psychosocial and spiritual support for their care-givers (124).

Providing sustainable care across the continuum from diagnosis to the end of life requires multidisciplinary collaboration and active cooperation of different care providers and coordinated continuity of health and social care across different system levels. The skills and capacity of the workforce and services are often challenged by the complex needs of people at different stages of dementia. Training and education should focus more on the changing patterns of service needs and the skillset to accommodate such changes or make adequate referral whenever needed (125). Careful planning and optimization of the service configurations to accommodate increasing needs warrant continuous high-level commitment from the government and advocating NGOs (126). Mechanisms for data-sharing and communication across care settings are some of the most important actions for ensuring the continuum of care across different settings (127).

Moreover, our findings contribute to the expanding body of literature emphasizing the importance of multidisciplinary collaboration in dementia care. Saint-Pierre et al. illustrated that integrated care models involving healthcare professionals, social workers, and community organizations correlate with improved outcomes for individuals with dementia and their caregivers, thereby advocating for a holistic approach to care. Overall, our study contributes to the existing body of literature by offering empirical evidence of the efficacy of community-based care models and multidisciplinary collaboration within the framework of the Macao Dementia Policy.

### 4.4 Shaping dementia research for Macao

A holistic approach to strategic research effort is needed for addressing the dementia challenge from different aspects (128, 129). To this end, the WHO has developed a blueprint for dementia research (128) in which dementia research has been summarized across 6 major themes: (1) dementia epidemiology and economics; (2) dementia disease mechanisms and models; (3) dementia diagnosis; (4) drug development and clinical trials for dementia; (5) dementia care and support; and (6) dementia risk reduction. However, as shown in this study, the dementia research in Macao appeared to uncoordinated and imbalanced, with the majority of dementia research funded and conducted locally often related to basic and pre-clinical research and a disproportionately small number of studies focusing on medical and societal demand.

Disparities in funding interests and opportunities, inadequate access to patient data, insufficient research capacities across the entire dementia research spectrum could at least partly explain the situation. A good understanding of the disease burden, the gaps in the local needs for dementia care and support, the most relevant risk factors, and the cost-effectiveness of different interventions adopted under the dementia policy is crucial to determine what needs to be done and how to achieve predefined outcome more efficiently (130). Evidence should be available to inform investments in preventive efforts, treatments, services and carer support wherever possible (131). In addition to basic and clinical research, increased efforts in the studies about knowledge translation and exchange as well as the implementation of such research into practice and policy should also be encouraged (132, 133). To facilitate scientific data sharing and collaboration for advancing dementia care in Macao, the government can review existing laws and regulations concerning data governance, sharing and protection or develop new ones. Similarly, Milne et al. emphasized that effective data governance is essential for maximizing the potential of digital data in advancing dementia research and improving patient outcomes (134).

### 4.5 Lessons learnt from the Macao experiences

Macao’s policy on dementia has significant implications for other countries facing similar challenges. First, robust resource integration can provide better support for dementia patients and their caregivers, enhancing their quality of life. Second, Macao’s community-based comprehensive dementia care model focuses on their communities and promote social inclusivity. Third, a collaborative approach involving key stakeholders like government agencies, NGOs, and academic institutions ensures comprehensive policies that address the needs of dementia patients and caregivers while improving public-private relationships. NGOs can play a critical role in reaching individuals with dementia in the community, raising awareness and eradicating stigma. Additionally, coordination between different professions can increase patient-centered care by enhancing understanding and communication.

As shown in Supplementary File, the Macao government and NGOs have collaborated to promote dementia-friendly societies, with a focus on integrated dementia services, multidisciplinary diagnosis and treatment, and improved medical services and support. Dementia-specific facilities have been established to decrease the disease burden on social and individual level. A distinctive network system has been gradually established for dementia patients through a public-private partnership. According to Lo et al., healthcare professionals in Macao have sufficient knowledge and a positive attitude toward dementia care (135). However, while organizations have started promoting dementia in high schools, students lack adequate dementia care understanding and practice inappropriate preventive care (136). Consequently, it is vital to continue promoting dementia prevention starting from younger generations.

### 4.6 Study strengths and limitations

This review is comprehensive and methodical in gathering information on relevant policies, strategies, programs, and activities related to dementia in Macao. We utilized the health policy triangle framework for data collection and analysis. However, there are limitations to this study. The data was obtained from officially public documents and media sources. Thus, unpublished policies were not included. Additionally, there was no original full text available for the Macao Dementia Policy. Furthermore, the data was collected from various sources, including social media, websites, and newspapers, which may have led to some dementia-related activities being excluded. The overall findings are reported within the context of what is currently known. It is recognized that other external and internal evaluation reports are emerging and the evaluation team drew on available reports to inform the final synthesis of the evaluation data.

## 5 Conclusion

The present policy analysis outlined the gradual policymaking process, contextual factors, and influential persons. As the study revealed several limitations and improvement areas after the implementation of the Macao Dementia Policy, health researchers and the public can participate in dementia prevention policymaking to increase public awareness and improve the quality of life of people with dementia and their caregivers. To effectively execute policies in a local region, a transparent, sustainable healthcare system is needed. Seamless collaboration between the public and private sectors is also a key to best dementia practice.

## Author contributions

PT: Conceptualization, Data curation, Methodology, Writing – original draft, Writing – review & editing. ZC: Conceptualization, Data curation, Writing – original draft, Writing – review & editing. YZ: Data curation, Formal analysis, Writing – review & editing. JS: Data curation, Methodology, Writing – review & editing. HH: Conceptualization, Data curation, Formal analysis, Methodology, Supervision, Writing – review & editing. CU: Conceptualization, Data curation, Formal analysis, Methodology, Supervision, Writing – review & editing.
