# Supplementary material for: Implementation of the Macao dementia policy: a scoping review for the way forward
Source: Front Public Health. 2024 Jul 15;12:1400172. doi: 10.3389/fpubh.2024.1400172 (PMC11284116; doi:10.3389/fpubh.2024.1400172)
Supplement: Supplementary file 1 [file Data_Sheet_1.docx]

**Supplementary file**

**1. Search Strategy**

Table 1. Web of Science search strategy

| **Web of Science** | | |
| --- | --- | --- |
| #1 | ALL=(Macao or Macau) | 55669 |
| #2 | ((((((((TI=(dementia)) OR TI=(dementia care)) OR TI=(Dementia, Multi-Infarct)) OR TI=(Frontotemporal Dementia)) OR TI=(Alzheimer Disease)) OR TI=(Dementia, Vascular)) OR TI=(AIDS Dementia Complex)) OR TI=(Lewy Body Disease)) OR TI=(Nootropic Agents) OR TI=(cognitive dysfunction) OR TI=(neurodegenerative) OR TI=(cognitive impairment) | 198243 |
| #3 | ((((((((AB=(dementia)) OR AB=(dementia care)) OR AB=(Dementia, Multi-Infarct)) OR AB=(Frontotemporal Dementia)) OR AB=(Alzheimer Disease)) OR AB=(Dementia, Vascular)) OR AB=(AIDS Dementia Complex)) OR AB=(Lewy Body Disease)) OR AB=(Nootropic Agents) OR AB=(cognitive dysfunction) OR AB=(neurodegenerative) OR AB=(cognitive impairment) | 369010 |
| #4 | ((((((((TS=(dementia)) OR TS=(dementia care)) OR TS=(Dementia, Multi-Infarct)) OR TS=(Frontotemporal Dementia)) OR TS=(Alzheimer Disease)) OR TS=(Dementia, Vascular)) OR TS=(AIDS Dementia Complex)) OR TS=(Lewy Body Disease)) OR TS=(Nootropic Agents) OR TS=(cognitive dysfunction) OR TS=(neurodegenerative) OR TS=(cognitive impairment) | 519677 |
| #5 | #2 or #3 or #4 | 519677 |
| #6 | #1 AND #5 | 582 |

Table 2. PubMed search strategy

| **PubMed** | | |
| --- | --- | --- |
| #1 | (Macao*) OR (Macau*) | 14993 |
| #2 | ((((dementia care[Title/Abstract]) OR (((Dementia, Multi-Infarct[MeSH Terms]) OR (Frontotemporal Dementia[MeSH Terms]) OR (Alzheimer Disease[MeSH Terms]) OR (Dementia, Vascular[MeSH Terms]) OR (AIDS Dementia Complex[MeSH Terms]) OR (Lewy Body Disease[MeSH Terms]) OR (Nootropic Agents[MeSH Terms])) OR (dementia[MeSH Terms]))) ) OR (cognitive dysfunction[MeSH Terms])) OR (Neurodegenerative Diseases[MeSH Terms]) | 436768 |
| #3 | #1 AND #2 | 356 |

Table 3. Scopus search strategy

| **Scopus(27.10.2022)** | | |
| --- | --- | --- |
| #1 | ALL  ( macao   OR   macau ) | 66177 |
| #2 | ( TITLE-ABS-KEY ( dementia ) ) OR ( TITLE-ABS-KEY ( dementia AND care ) ) OR ( TITLE-ABS-KEY ( dementia, AND multi-infarct ) ) OR ( TITLE-ABS-KEY ( alzheimer AND disease ) ) OR ( TITLE-ABS-KEY ( frontotemporal AND dementia ) ) OR ( TITLE-ABS-KEY ( dementia, AND vascular ) ) OR ( TITLE-ABS-KEY ( aids AND dementia AND complex ) ) OR ( TITLE-ABS-KEY ( lewy AND body AND disease ) ) OR ( TITLE-ABS-KEY ( nootropic AND agents ) ) OR ( TITLE-ABS-KEY ( cognitive AND dysfunction ) ) OR ( TITLE-ABS-KEY ( cognitive AND impairment ) ) OR ( TITLE-ABS-KEY ( neurodegenerative ) ) | 614812 |
| #3 | #1 AND #2 | 873 |

Table 4. MEDLINE search strategy

| **MEDLINE** | | |
| --- | --- | --- |
| #1 | TX Macao or Macau | 446 |
| #2 | AB dementia or Dementia, Multi-Infarct OR Frontotemporal Dementia OR Alzheimer Disease OR Dementia, Vascular OR AIDS Dementia Complex OR Lewy Body Disease OR Nootropic Agents OR cognitive dysfunction OR cognitive impairment OR neurodegenerative | 120457 |
| #3 | TI dementia or Multi-Infarct Dementia OR Frontotemporal Dementia OR Alzheimer Disease OR Vascular Dementia OR AIDS Dementia Complex OR Lewy Body Disease OR Nootropic Agents OR cognitive dysfunction OR cognitive impairment OR neurodegenerative | 51169 |
| #4 | S2 OR S3 | 128330 |
| #5 | S1 AND S4 | 163 |

Table 5. CNKI & WanFang search strategy

| **CNKI (03.02.2023)** | | |
| --- | --- | --- |
| #1 | (摘要%失智症 + 老年失智症 + 血管性失智症 + 老人痴呆症 + 亞茲海默 + dementia) | 51725 |
| #2 | Macao | 44938 |
| #3 | #1 AND #2 | 15 |
| **WanFang (03.02.2023)** | | |
| #1 | 主題:(失智癥 OR 老人癡呆癥 OR 亞茲海默 OR 血管性失智癥 OR dementia OR Alzheimer's disease) | 49093 |
| #2 | 主題:(澳門 or Macao or Macau) | 30896 |
| #3 | #1 and #2 | 3 |

**2. Findings of scoping review**

| Year | Action areas | | | | Key players | Collaborating | Events |
| --- | --- | --- | --- | --- | --- | --- | --- |
|  | Health services | Training | Community | Research |  |  |  |
| **Prior to 2016 (Pre-dementia policy)** | | | | | | | |
| 2008 | Work Group |  |  |  | Comissão para os Assuntos do Cidadão Sénior |  | The Elderly Commission commenced operation in 2008[1] |
| 2009 |  |  | Interrogatory |  | Legislator Leong Heng Teng |  | Question on how to strengthen the prevention of dementia[2] |
|  |  |  | Increased services and support for dementia patients |  | Social Welfare Bureau |  | Expanded professional support for elderly service providers and caregiver; strengthened care services for dementia elderlies; organized training courses on the care of people[3] |
|  | Preparation |  |  |  |  |  | Discuss the improvement and optimization of dementia services[3] |
| 2010 | Exchange the care of dementia patients |  |  |  | Comissão para os Assuntos do Cidadão Sénior |  | Visit Beijing and Shanghai to investigate services for the elderly[4] |
|  |  |  |  | Policy analysis about dementia care services | The Hong Kong Polytechnic University |  | An analysis of the development of dementia care services in Macao : policies and resources allocation[5] |
|  |  |  |  | To assess the care needs of dementia elderly and their caregivers | Kiang Wu Nursing College |  | Community screening of elderly dementia patients in the early stages of dementia in Macau 2010.12-2012.03[6] |
|  |  |  | Establishment of an association related to dementia |  | Macau Alzheimer's Disease Association |  | Organizing Committee was established[7] |
| 2011 | Future development of dementia care |  |  |  | Chief Executive | Secretary for Social Affairs and Culture; Director of the Chief Executive's Office; Secretary of the Executive Council and Adviser to the Chief Executive's | Visit the facilities of Centro I Chon da União Geral das Associações dos Moradores de Macau (day care center) for understanding the care services for the frail and dementia elderly and its work in lifelong education for the elderly.[8] |
|  | Future development of dementia care |  |  |  | Social Welfare Bureau |  | Services for elders with dementia and their future development[9] |
|  |  | HCP training | Increased specific services for dementia patients |  |  |  | Additional services for dementia patients in some elderly service facilities; set up a special are in a day care center and a residential service area in a nursing home for dementia elderlies; provide professional training on dementia to staff of elderly services[9] |
|  | Future development of dementia care |  |  |  |  |  | Carry out community education and professional training programmes on dementia to enhance the awareness of the elderly and the general public on dementia[10] |
|  |  |  | Lobbying/promotion |  | Health Bureau |  | lectures on dementia[11]-[14] |
|  |  | HCP training | Public education  Service provision to patients | Dementia-related research | Kiang Wu Nursing College of Macau | União Geral das Associações dos Moradores de Macau  Macao Federation of Trade Unions  Caritas Macau  The Women' s General Association of Macau  Macau Alzheimer's Disease Association | Initiate Benevolence Lights up my Later Life education system project[15] |
|  |  |  | Lobbying/promotion |  |  |  | 6 Public lectures [16] |
|  |  | Caregiver training | Lobbying/promotion |  |  |  | 4 Caregiver Training Workshop[16] |
|  |  |  | Lobbying/promotion |  |  |  | 9.21 International Dementia Day Seminar for Hong Kong and Macao[16] |
|  |  |  |  | Comparison burden on caregivers in different regions |  |  | Cross-Site Comparison of Dementia Family Caregiver Burdens 2011.04-2012.06 [17] |
|  | Early diagnosis |  |  |  | Conde S. Januário Hospital |  | Established memory clinic[15] |
|  |  |  | Patient group support |  | Macau Alzheimer's Disease Association |  | Association was formed[18] |
| 2012 | Work group/point of responsibility |  |  |  | Administration |  | Set up an inter-departmental study group on Macao's pension security mechanism[19] |
|  |  |  | Lobbying/promotion |  | Health Bureau |  | Seminar on "Home Care for Dementia Patients"[20] |
|  |  | Caregivers' training | Lobbying/promotion |  |  |  | Conde S. Januário Hospital provided health seminars on how to care for patients with dementia.[21] |
|  | Work meeting |  |  |  | Comissão para os Assuntos do Cidadão Sénior |  | Two plenary meetings (2012.03.28 & 2012.09.27)[22]-[23] ^,^ |
|  |  |  |  | Reduce the burden on caregivers | Kiang Wu Nursing College of Macau |  | A health promotion program to reduce the care burden of five dementia caregivers[24] |
| 2013 |  |  | Dementia-specific facility |  | The Government |  | An economic housing established a support center for the elderly and their families with dementia[25][24] |
|  | Subsidy |  |  |  | Social Welfare Bureau |  | Inclusion of dementia in disability benefits and care benefits under special assistance for Vulnerable persons[26] |
|  |  |  | lobbying/promotion |  | Healthy City Council and Health Bureau | Government, professional groups and civil society organizations | A series of activities have been organized for the mental health of the whole population[27] |
|  | Work meeting |  |  |  | Comissão para os Assuntos do Cidadão Sénior |  | Plenary meeting (2013.04.16) [28] |
|  | Early diagnosis |  |  |  | Kiang Wu Nursing College of Macau |  | Set up a memory center and open a hotline for dementia services[15] |
|  |  |  |  | Evaluate the user's recognition of the memory center |  |  | Dementia Community Service Action Research Project - Creating a Memory Center 2013.01-2014.06 [29] |
|  |  |  |  | Assessing users' opinions on the content, convenience, and attitude of the dementia telephone service hotline |  |  | Dementia in the Elderly Community Action Research Project - Creating a Dementia Telephone Service Hotline 2013.01-2014.06[30] |
|  |  |  |  | Investigated the differences in sleep-wake patterns |  |  | Differences in sleep of dementia residents between Macao (China) and Sydney (Australia)[31] |
|  |  |  |  | Evaluate the efficacy of community support services for dementia patients, as well as the content and forms of training for informal and family caregivers |  |  | Dementia Community Service Action Research Project on Enhancing Community Support Services and Training Facilities for the Elderly with Dementia 2013.01-2016.06[32] |
|  |  |  |  | Dementia conference | Kiang Wu Nursing College of Macau, Hong Kong Alzheimer's Disease Association | Alzheimer's Disease International, Macau Alzheimer's Disease Association | The 16th International Dementia Association Asia Pacific Symposium[33] |
|  |  |  |  | Became an international member | Macau Alzheimer's Disease Association |  | Become a member of Alzheimer's Disease International[18] |
|  |  | Caregivers' training |  |  |  | Education and Youth Affairs Bureau | Basic Training Workshop for Dementia Caregivers[34] |
|  |  |  | Dementia-specific day care center |  | Caritas Macau |  | A special area for dementia care has been set up in Asilo S. Francisco (aged care facility)[35] |
| 2014 |  |  |  | Public feedback for current situation | Administration |  | Opinion gathering groups were organized to sort out study reports on pension status and policies in Macao, such as: dementia assessment instruments are not united, and public and private systems are not coordinated. Dementia support services and professionals are lacking. Residents are unaware of dementia and related services and lack training.[36] |
|  |  |  |  | Delegate other association for planning Macao dementia policy | Social Welfare Bureau |  | Commissioned the Hong Kong Alzheimer's Disease Association to conduct a study on the future planning of dementia services in Macao and put forward recommendations on short -, medium - and long-term dementia services policies and measures[37] |
|  | Early diagnosis |  |  |  | Health Bureau |  | Taipa Elderly Health Station has set up elderly care consultation clinics, while other Health Center have set up mental health clinics[38] |
|  |  |  | Lobbying/promotion |  |  |  | Lecture "Know It, Face It, Dementia"[39] |
|  |  | Caregivers' training | Interdepartmental & community associations' cooperation; lobbying/promotion |  |  |  | Organizing dementia promotion programmes and caregiver training programmes in collaboration with various government departments and community organizations[38] |
|  |  |  | Interdepartmental & community associations' cooperation; lobbying/promotion |  |  |  | Establish community support and interdepartmental referral mechanisms, subsidize non-profit-making organizations to set up rehabilitation centers and provide home care services, and establish a number of psychological counselling sites in the community[38] |
|  |  |  | Interrogatory |  | Legislator Zheng Anting |  | To promote a written inquiry on measures to care for patients with dementia & received feedback from the government[15][40] |
|  |  |  | Interrogatory |  | Legislator Wong Kit Cheng |  | Written inquiry about focus on dementia prevention in Macao & received feedback from the government[38][41] |
|  |  |  | Lobbying/promotion |  | Kiang Wu Nursing College of Macau | Caritas Macau | Public lecture on "Use Your Brain Not to Grow Old: Activities to Refresh Your Brain and Strengthen your Wisdom" at Escola São João De Brito(High school) [42] |
|  |  |  | Lobbying/promotion, dementia-specific care services |  | Women’s Commission |  | Visit Complexo de Servicos de Apoio ao Cidadao Senior "Pou Tai" (nursing home), which also has services for caregivers and dementia patients[43] |
|  |  | Caregiver & HCP training |  |  | Caritas Macau |  | Training Course on Routine Activities of the Elderly with Dementia in the Elderly Care Series & Training Course on Frontline Care for Dementia Patients[44] |
|  |  |  | Lobbying/promotion |  |  |  | Nursing Home "Sol Nascente" of Areia Preta launched the "Wisdom is With You" Dementia Promotion Programme[44] |
| 2015 | Enhance dementia-related service; provide additional resource to community service |  |  |  | The government |  | Early prevention measures and care services for patients with dementia would be strengthened. Additional resources would be allocated for community outreach activities.[36] |
|  |  |  |  | Comprehensive analysis of the needs of the elderly |  |  | International Policy and Theory Research Report on Aging[45] |
|  |  |  |  | Construction of dementia friendly community, implementation of elderly policy guidelines |  |  | Macau Dementia Strategic Framework (2016-2025)[47]– internal review[46] |
|  | Public consultation |  |  |  | Social Welfare Bureau |  | Ten-year action plan for the elderly in Macau (2016-2025): public consultation, press conference[46] |
|  |  |  |  | What are the needs of the elderly in Macao now and in the future |  |  | Research Report on the Situation and Policies of the Elderly in Macao[36] |
|  | Organized an elderly specific website |  |  |  |  |  | Set up an information network on Services for the Elderly in Macao[48] |
|  |  |  | Lobbying/promotion |  |  |  | The Secretary for Social and Cultural Affairs and his delegation visited the Psychiatric building in Taipa and the geriatric psychiatric unit for the elderly with dementia[49] |
|  |  |  | Lobbying/promotion |  |  |  | Secretary for Social and Cultural Affairs visits the Dementia Service area of Complexo de Servicos de Apoio ao Cidadao Senior "Pou Tai" (nursing home)[50] |
|  |  |  | Lobbying/promotion |  |  |  | Co-hosted the "Community Care Dementia Promotion – Youth Ambassador for Benevolence Lights up my Later Life” [48] |
|  |  |  | Lobbying/promotion |  |  | Kiang Wu Nursing College of Macau | The "Community Care Dementia Promotion - Youth Ambassador for Benevolence Lights up my Later Life" promotion achievements show[51] |
|  |  |  | Lobbying/promotion |  | Health Bureau |  | Lecture "Know It, Face It, Dementia"[52] |
|  | Work meeting |  |  |  | Comissão para os Assuntos do Cidadão Sénior |  | Plenary meeting (2015.03.04), about dementia prevention and treatment services[53] |
|  |  |  | Interrogatory |  | Legislator Chan Mei I |  | Written inquiry on dementia prevention and treatment & received feedback from the government^,^[54][55] |
|  | Early diagnosis & treatment |  |  |  | Kiang Wu Hospital |  | Memory clinic establishment[56] |
| **2016 (Launch of Dementia Policy)** | | | | | | | |
|  |  |  | Lobbying/promotion |  | Health Bureau & Social Welfare Bureau | UGAMM  Macao Federation of Trade Unions  Caritas Macau, Complexo Pou Tai elderly service center, Obra das Mães, Holy House Of Mercy Of Macau, Canossian Daughters of Charity, MADA | Meeting: Briefing on the current situation and development of dementia services in Macao, and listening to the views of service providers on service policy[57] |
|  | Screening in elderly service organizations | HCP training |  |  |  |  | To support civil society organizations in providing training in screening for the staff of the three elderly service providers, as well as screening for dementia for the elderly in the agencies concerned, and providing referrals for suspected patients to medical units. [58] |
|  | Early diagnosis and treatment; | Patient education and training | Social service support |  |  |  | Setting up a Dementia Medical center; Implement Macao dementia policy; Announced the launch of the Macao Dementia Service Network[59] |
|  | Obtain real data for strengthening policy formulation, patient management and service follow-up |  |  |  |  |  | Establish a dementia registration system[59] |
|  |  |  | Lobbying/promotion |  |  |  | The radio programme "Macau Seminar" explored the prevention and support of dementia[60] |
|  |  |  | Lobbying/promotion |  | Social Welfare Bureau |  | To produce a "Dementia Awareness" pamphlet and a "Walk with You" dementia video[61] |
|  | Enact action plan and dementia policy |  |  |  |  |  | The Pension Protection Mechanism and the ten-year Action Plan for Elderly Services from 2016 to 2025 have been launched[62] |
|  |  |  | Lobbying/promotion |  |  |  | Macau Elderly Protection Mechanism and Ten-Year Action Plan for Elderly Services 2016-2025 Promotional Leaflet[63] |
|  |  |  | Lobbying/promotion |  |  |  | List of Macau ten-Year Action Plan for Elderly Services from 2016 to 2025[64] |
|  | Annual report |  |  |  |  |  | 2016 Annual Review Report of Macau Elderly Protection Mechanism and Ten-Year Action Plan for Elderly Services 2016-2025[58] |
|  | Annual report |  |  |  |  |  | Executive List of Macau Elderly Protection Mechanism and Ten-Year Action Plan for Elderly Services 2016-2025[65] |
|  |  |  | Adding dementia service center |  |  |  | Additional dementia day care service at one nursing home and assisted in transforming one Day Care Centre for the Elderly into a dementia day Care Centre (specialized in serving elders with mild and moderate dementia) [66] |
|  |  |  | Lobbying/promotion |  |  |  | Commissioned Kiang Wu Nursing College to produce a pamphlet "Understanding Dementia"[67] |
|  |  | HCP training |  |  |  |  | Training courses on dementia, personal care planning and quality management were organized for 14 long-term care facilities for the elderly[58] |
|  |  | Caregivers’ training |  |  |  |  | Support Kiang Wu Nursing College organized 1 training and 4 workshops for 18 home-based caregivers[58] |
|  | Discussion for dementia prevention |  |  |  |  |  | Meeting with the Chairman of the Macao Alzheimer’s Disease Association, listening to the association's opinions and suggestions on elderly services and dementia prevention and treatment in Macao[68] |
|  | Work group/point of responsibility |  |  |  | Health Bureau |  | Establish a dementia work group in the Committee on Chronic Disease Control[69] |
|  |  | HCP training |  |  |  | Hong Kong Alzheimer’s Disease Association | To organize cognitive function assessment courses in Macao[70] |
|  | Work group/point of responsibility |  |  |  |  |  | Thirteen public departments formed an inter-departmental Steering group for the Pension Security Mechanism[71] |
|  | Work meeting |  |  |  | Comissão para os Assuntos do Cidadão Sénior |  | Plenary meetings (2016.04.08; 2016.10.18)[72][73] |
|  |  |  | Lobbying/promotion |  |  | Pension security mechanism inter-departmental implementation group | Visit Dementia Medical Center[74] |
|  | Work meeting |  |  |  | Committee for the Control of Chronic Diseases |  | Plenary meeting (2016.10.25) [75] |
|  |  |  | Interrogatory |  | Legislator Ho Ion-sang |  | Written inquiry about the improvement of dementia care service model and service resource overlapping & received feedback from the government^,^[76][77] |
|  |  | HCP training |  |  | Kiang Wu Nursing College |  | Two community education training courses on dementia for 47 healthcare professionals[58] |
|  |  |  | Sharing the outcomes of dementia activities |  |  |  | Monthly meeting to spread dementia knowledge[78] |
|  |  |  | Lobbying/promotion |  |  |  | Held the "Benevolence Lights up my Later Life Social Education System for the Aging Project 5th Anniversary Carnival".[79] |
|  | Screening |  |  |  |  | Dr. Stanley Ho Medical Development Foundation, MADA | Further expansion of services in the "Memory Center" was discussed; the "Macao Community Dementia Screening Program for the Elderly" was launched[78] |
|  |  |  |  | Examining the early stage dementia of the elderly in the community, as well as the care needs of those with dementia and their families |  |  | Analysis of Early-Stage Dementia Screening among Elderly Population in Macao's Community. [81] |
|  |  |  |  | Exploring the most affect life satisfaction of elderly people living in community |  |  | Common chronic health problems and life satisfaction among Macau elderly people. [82] |
|  |  |  | Lobbying/promotion |  |  | Social Welfare Bureau | Publication of "Recognizing Dementia" booklet[75] |
|  |  |  | Continue to advance the prevention and treatment of dementia |  | Macau Alzheimer’s Disease Association |  | Held the 2nd Annual General Meeting 2016[83] |
|  |  |  | Lobbying/promotion | International conference |  | Kiang Wu Nursing College | Attended the 31st Member States Conference and International Alzheimer’s Disease Symposium[84] |
|  |  |  | Non-pharmacological intervention |  | Caritas Macau |  | St. Francis aged care facility organized vegetable planting for residents with dementia[85] |
|  |  |  | Lobbying/promotion |  | Macao Carers Association |  | "Dementia Assessment and Treatment Services in Macau" visit and exchange program[86] |
|  |  |  | Lobbying/promotion |  | Chinese Federation for Analytical Psychology |  | Dementia-themed movie: "Tomorrow, Don't Be Late[87] |
|  |  |  | New service for dementia patients |  | União Geral das Associações dos Moradores de Macau |  | Launched the new service "Brainiac World" Toy Library[88] |
| After 2016 (Post-Dementia Policy) | | | | | | | |
| 2017 |  |  | HCP training |  | Social Welfare Bureau | Kiang Wu Nursing College | Macao Kiang Wu College of Nursing was commissioned to organize a training course for dementia care planners[89] |
|  | Annual report |  |  |  |  |  | 2017 Annual Review Report of Macau Elderly Protection Mechanism and Ten-Year Action Plan for Elderly Services 2016-2025[90] |
|  |  |  | Lobbying/promotion | International conference |  |  | The Secretary for Social Affairs and Culture attended the opening ceremony of the Global Summit on Alzheimer's Research and Care at the Champalimaud Foundation in Lisbon. [91] |
|  |  |  | Increased day care facilities for dementia patients |  |  |  | Two additional day care centers for dementia patients[92] |
|  |  |  | Suggestions from NGO |  |  | União Geral das Associações dos Moradores de Macau, Macao Holy House of Mercy, Macau Deaf Association | The Secretary for Social Affairs and Culture held a seminar with representatives from the social service sector to listen to their views and suggestions on the administration of social and cultural issues. [93] |
|  |  |  | Lobbying/promotion |  |  | Health Bureau, MADA, Chronic Disease Prevention and Control Committee and multiple societies across Macao | A forum entitled "Healthy Macau, Happiest Home - Building a Dementia Friendly Community in Macau" was organized, as well as a reading ceremony for the "Macau SAR Dementia Friendly Community Charter." (2017.10.22) [94] |
|  | New service - dementia patient call services |  |  |  |  |  | Dementia Call Service for the Elderly will be launched in 2018[95] |
|  |  |  | New elderly integrated service center |  |  |  | The first four-in-one elderly service complex will be opened in November this year[96] |
|  |  |  |  |  | Health Bureau | Hong Kong Alzheimer’s Disease Association | Two sessions of "Certificate in Cognitive Functioning Assessment" for healthcare staff from the Health Bureau, Social Welfare Bureau, Kiang Wu Nursing College, health centers and nursing homes, day care centers for the elderly, and other non-profit organizations[97] |
|  |  |  | HCP training |  |  |  | Dementia Seminar for Private Practitioners[98] |
|  |  |  | Civil servant training |  |  |  | Dementia Prevention Seminar for Public Officers and their families [99] |
|  |  |  | HCP training |  |  |  | A 3-day training workshop for professionals in elderly care facilities on the topic of "Dementia - Care Service Design and Intervention".[90] |
|  |  |  | HCP training |  |  |  | A 4-day training workshop on "Developing a Personal Care Plan" for professionals in elderly care facilities[90] |
|  |  |  | HCP training |  |  |  | Training Dementia Service Planner[100] |
|  |  | Increase health service |  |  |  |  | Increased number of outpatient sessions and services at the Dementia Medical Center[101] |
|  |  | Achieved early diagnosis and detection |  |  |  |  | From January to September 2017, the dementia clinic's new staff increased nearly twofold compared to the same period last year, and the dementia diagnosis rate increased from 37.5% to 45%.[94] |
|  |  |  | Lobbying/promotion |  |  |  | Add dementia information to the Health Bureau mobile app and website[102] |
|  |  |  | Lobbying/promotion |  |  |  | Health centers held a total of 562 health education activities on dementia[102] |
|  |  |  | Lobbying/promotion | International conference |  |  | Representatives of Health Bureau attended the 2nd Beijing International Neurodegenerative Diseases Conference of the Chinese Society of Microcircuitry and Neurodegenerative Diseases[103] |
|  | Work meeting |  |  |  | Comissão para os Assuntos do Cidadão Sénior |  | Plenary meetings (2017.02.28 & 2017.10.13) reviewed progress on the implementation of the 10-year Action Plan for Seniors 2016-2025; especially dementia services[104][105] |
|  | Work meeting |  |  |  | Chronic Disease Prevention and Control Committee |  | Plenary meetings (2017.03.28 & 2017.09.26) reviewed and suggested dementia advocacy model[106][107] |
|  |  |  | Promotion |  |  |  | Chronic Non-Communicable Disease Newsletter No. 6 Promoting the MIND Diet may help reduce the risk of dementia[108] |
|  | Work meeting |  |  |  | Interdepartmental Steering Group on Pension Security Mechanism |  | Plenary meetings (2017.03.24 & 2017.11.27) discussed on the current status and development of dementia services^,^[109][110] |
|  | Work meeting |  |  |  | Dementia Work Group |  | Four work meetings (2017.01.04, 2017.02.15, 2017.03.29 & 2017.05.17)[102] |
|  |  |  | Lobbying/promotion |  |  |  | Introduction to Dementia Seminar to Pui Ching Middle School Students' Association[102] |
|  |  |  |  | Conference |  |  | New Advances in Geriatrics Forum[102] |
|  |  |  | Lobbying/promotion |  |  |  | Promote prevention messages to the public through newspapers on World Alzheimer’s Day[102] |
|  |  |  | International organization visit |  | Kiang Wu Nursing College |  | The Chief Executive of Alzheimer’s Disease UK visited the College to learn more about the project of Benevolence Lights up my Later Life and the dementia community screening programme[111] |
|  |  |  |  | Home-based dementia caregivers felt an increased burden in the caregiving process, which affects physical, emotional and social aspects |  |  | Localization of the Dementia Caregiver Support Program in Homes 2017.11-2018.11[112] |
|  |  |  | Lobbying/promotion |  |  |  | Benevolence Lights up my Later Life organized carnival[113] |
|  |  |  |  | International conference |  |  | Visit to Plymouth, a dementia-friendly community in the South of England, to attend the Alzheimer’s Disease International Conference 2017[114] |
|  |  | HCP training |  |  |  | Hong Kong Alzheimer’s Disease Association, MADA | Co-organized a three-month 80-hour Dementia Care Planner Course[115] |
|  |  |  | Organization cooperation; promotion |  |  | The Women’s General Association of Macau | Signed partnership agreement to conduct public seminars and caregiver workshops on dementia prevention and care for the elderly[116] |
|  |  | Workshop for caregivers | Lobbying/promotion |  | Kiang Wu Hospital | The Women’s General Association of Macau | Seven public seminars on "Prevention and Care of Dementia in the Elderly" and workshops on caring for the elderly[117] |
|  |  | Volunteer training |  |  | União Geral das Associações dos Moradores de Macau | Conde S. Januário Hospital | Centro de Lazer e Recreacao dos Anciaos (aged care facilities) conducted Senior Volunteer Training Workshop[118] |
|  |  |  | Lobbying/promotion |  |  |  | A medical center held 7 dementia seminars for the public[102] |
|  |  |  | Promote dementia awareness and early detection, and improve HCP training |  | MADA |  | Annual General Meeting to consider and approve the reports[119] |
|  | Screening |  |  |  |  |  | 257 seniors completed cognitive screening[102] |
|  |  |  | Non-pharmacological therapy |  | Caritas Macau |  | Horticultural therapy and art therapy at one nursing home[120] |
|  |  | Caregivers’ training | Lobbying/promotion |  | The Women' s General Association of Macau |  | Held 5 dementia seminars for the public; one food and activities guideline for dementia prevention; 6 caregivers’ training workshops[102] |
|  |  |  | Lobbying/promotion |  | The Pharmaceutical Society of Macau |  | Seminar on dementia care and safe and rational use of medication[121] |
|  |  |  | Lobbying/promotion |  | Macau Social Services Coordination Office |  | Held Dementia Treatment Events: New Directions[122] |
|  |  | Caregivers’ training |  |  | Macao Carers Association |  | Caregivers’ participation in Dementia and Coronary Heart Disease Care Skills Course[123] |
|  |  |  | Lobbying/promotion |  | Macao Daily News |  | Interviews with dementia patients and their families, from the patient's and caregiver's perspective, as well as explanations from medical professionals, emphasizing the importance of early detection and treatment[102] |
|  |  |  | Lobbying/promotion |  | Macau Magazine |  | Issue 116: "Dementia Awareness, Prevention, and Proper Care[124] |
| 2018 | Enlarging services for dementia patients |  |  |  | Administration |  | Increased the number of dementia day service places[125] |
|  | Specialized Outreach Medical Service Team |  |  |  | Social Welfare Bureau & Health Bureau | 11 subsidized Residential Respite Service for Elders | A meeting was held to explain the Specialty Outreach Program, which will consist of the Conde S. Januário Hospital Geriatric, Internal Medicine, Psychiatry, Emergency Department, Physiotherapy and other specialties to form the "Specialty Outreach Team".[126] |
|  |  |  | Lobbying/promotion |  |  | MADA & other societies | International forum on "Healthy Macau, Happiest Home - Building a Dementia Friendly Community"; representatives of organizations read the "Macau SAR Dementia Friendly Community Charter" and more than 100 organizations joined the event. [127] |
|  | New support service |  |  |  | Social Welfare Bureau |  | Organized outdoor emergency call-out service and officially launched the service in November[128] |
|  | Annual report |  |  |  |  |  | 2018 Annual Review Report of Macau Elderly Protection Mechanism and Ten-Year Action Plan for Elderly Services 2016-2025[129] |
|  |  |  | Lobbying/promotion |  |  | Healthy City Committee, Chronic Disease Prevention and Control Committee | Jointly organized the fourth "Walk for Health" walking event[130] |
|  |  |  | Lobbying/promotion |  | Health Bureau | Hong Kong Executive Institute | Co-organized seminar on elderly services[131] |
|  |  |  | Lobbying/promotion |  |  |  | Geriatrics Lecture Series[132] |
|  | Provision basic health service |  |  |  |  |  | Officially opened Ilha Verde Health Centre and would establish a dementia support center at Ilha Verde Health Centre[133] |
|  |  |  | Lobbying/promotion |  |  | Social Welfare Bureau, MADA | Seminar on "Dementia Friendly Community" and "Macao SAR Dementia Friendly Community Charter" Project[134] |
|  |  |  | Service center activity/promotion |  |  |  | Dementia Medical Center Hosts Dementia Outreach Event[135] |
|  |  |  | Lobbying/promotion |  |  |  | The Elderly Care Team of the General Health Care Sub-system of the Health Bureau visited three elderly centers to hold a promotion activity of "Care for Dementia, Love for Inclusion - Building a Dementia Friendly Community for Dementia"[135] |
|  | Investigating other country's facilities for dementia |  |  | International conference |  | Macao Health Bureau Delegation | Visit to UK Accessible Facilities and Dementia Friendly Communities, attend Plymouth 2018 Alzheimer’s Disease International Conference[136] |
|  | Diagnosis of dementia patients |  |  |  |  |  | More than 2,100 patients have been diagnosed with dementia at Dementia Medical Center in Macao[137] |
|  |  |  | Lobbying/promotion |  |  |  | Asia Pacific Regional Director of the Alzheimer’s Disease International visited the Dementia Medical Center[138] |
|  |  |  |  | Conference |  |  | The 17th China, Hong Kong and Macau Senior Health Administration Joint Conference[139] |
|  | Promote interprofessional cooperation | Patient/caregiver training and education | Social support service |  |  |  | Establishment of Dementia Support Center[140] |
|  |  |  | Lobbying/promotion |  |  |  | Health centers held a total of 579 health education activities on dementia[141] |
|  | The use of dementia medical center |  |  |  |  |  | Dementia Medical Center provided 369 case consultations to dementia patients and their families in 2018[141] |
|  | Policy development |  | Promotion |  | Comissão para os Assuntos do Cidadão Sénior |  | Exchange trip to Tokyo, Japan to learn from Japan's successful experience in elderly policies and services and served as an important reference for the continuous development of the pension security policy of the Macao SAR[142] |
|  | Work meeting |  |  |  | Chronic Disease Prevention and Control Committee |  | Plenary meetings (2018.03.20 & 2018.09.24) reported on the progress of dementia work in 2018 and planned work for 2019[143][144]. |
|  |  |  | Lobbying/promotion |  | Education and Youth Development Bureau |  | The 38th issue of Lifelong Learning magazine features dementia as a topic of discussion[145] |
|  | Work meeting |  |  |  | Dementia Work Group |  | 2 work meetings (2018.03.14 & 2018.07.18)[141] |
|  |  |  | Lobbying/promotion | Conference |  |  | The First Guangdong, Hong Kong, Macau and Greater Bay Area Health and Wellness Cooperation Forum[141] |
|  |  |  | Lobbying/promotion |  |  |  | Dementia Seminar[141] |
|  |  | Civil servant training |  |  |  |  | Continuous Public Health Training Courses[141] |
|  |  |  |  | International conference |  |  | 2018 South Lake Taiko International Conference on Cognitive Therapy and Care[141] |
|  |  | HCP training |  |  |  |  | Guangdong, Hong Kong, Macau and the Bay Area General Practitioner Cultivation Course[141] |
|  |  |  | Interrogatory |  | Legislator Wong Kit Cheng |  | Written question on concern about dementia prevention and family support measures in Macau & received feedback from the government[146][147] |
|  |  |  | Lobbying/promotion |  | Kiang Wu Nursing College | Alumni Association and MADA | MEMORY WALK was held and dementia publicity materials were distributed to visitors along the way. [148] |
|  |  |  | Lobbying/promotion |  |  |  | Publication of Dementia Friendly Community Brochure[149] |
|  |  |  |  | Understanding the awareness and attitudes of Macao citizens towards dementia |  |  | Survey on Public Awareness and Attitude towards Dementia in Macau 2018.01-2019.06[150] |
|  |  | HCP training |  |  | Kiang Wu Hospital |  | Dementia seminar[141] |
|  |  |  |  | Further appropriate strategies and need to improve access to treatment | University of Macau |  | Clinical characteristics and quality of life of older adults with cognitive impairment in Macao. Psychogeriatrics. [151] |
|  |  |  | Lobbying/promotion |  | Caritas Macau |  | Seminar on Dementia Service Practice Experience[152] |
|  |  |  | Service center activity |  |  |  | A seniors' center offers a magic seniors' developmental group[153] |
|  |  | Patient training |  |  |  |  | Day Care Center Home Care and Support Service Launches Home-Based Cognitive Training for Dementia Patients[153] |
|  |  |  | Community services recommendation |  |  |  | Written proposals submitted include strengthening the community support system for people with dementia[154] |
|  |  |  | Lobbying/promotion |  |  |  | A day care center hosts a carnival[155] |
|  |  |  | Visit and learn Taiwan dementia service |  |  |  | Visiting Taiwan Dementia Services[156] |
|  |  |  | Lobbying/promotion |  |  |  | Caritas Macau Elderly Services organized the 2018 Senior Citizens Day to promote awareness of dementia prevention among the elderly[157] |
|  |  |  | Lobbying/promotion |  | Macao Carers Association |  | Seminar on Macao Dementia Friendly Community[158] |
|  |  |  | Lobbying/promotion |  | Associação dos Familiares Encarregados dos Deficientes Mentais de Macau |  | Dementia Seminar[159] |
|  |  |  | Lobbying/promotion |  | Associação de Amizade de Insuficientes Renais de Macau |  | Dementia Seminar[160] |
|  |  |  | Lobbying/promotion |  | União Geral das Associações dos Moradores de Macau |  | A medical center held 6 dementia seminars for the public[141] |
|  |  |  | Lobbying/promotion |  | The Women's General Association of Macau | The Fu Lun Elders’ Association of Macau | Dementia Prevention Activities (carnival, group activities) [141][161]^,^ |
|  |  | Volunteers’ training | Lobbying/promotion |  |  |  | 6 Dementia Prevention Training Courses, Street Consultation Stations, Dementia Initial Examination Consultation Station[141] |
|  |  |  | Lobbying/promotion |  | Pui Ching Middle School |  | Dementia Seminar [162] |
|  |  |  | Lobbying/promotion | Conference | Cross-Straits Medicine Exchange Association |  | The 1st Cross-Straits, Hong Kong and Macau Regional Nursing Development Forum; Introduction of Dementia Medical Center[163] |
| 2019 |  |  | Lobbying/promotion |  | Social Welfare Bureau & Health Bureau & MADA |  | Seminar on Dementia Friendly Community Charter Project in Macau [164] |
|  |  |  | Lobbying/promotion |  | Social Welfare Bureau & Health Bureau | Representatives from social service organizations, medical professional associations, youth and student organizations | Three Dementia Friendly Alliance Networking Sessions and Dinners [165] |
|  | The use of dementia support center, enhanced conveniences, and improved services for dementia elderlies | Patient/caregiver training and education |  |  |  |  | The Dementia Support Center has completed the 1st session of 10 sessions of education and training courses for dementia patients and their caregivers, with a total attendance of 110 people; the Dementia Medical Centers cooperates with all Health Centers to provide convenient assessment and referral services to the public. Since the establishment of the center, the waiting time has been shortened from 6 months to less than 1 month, and the number of dementia patients diagnosed by the Health Bureau has increased by more than 600. [166] |
|  |  | Public training |  |  |  |  | Co-organized 6 Dementia Friendly Alliance training workshops[167] |
|  |  |  | New dementia care services |  | Social Welfare Bureau |  | The Social Service Division of the Methodist Church Cheerful Home officially launched its services, providing dementia care services[168] |
|  | Annual report |  |  |  |  |  | 2019 Annual Review Report of Macau Elderly Protection Mechanism and Ten-Year Action Plan for Elderly Services 2016-2025[169] |
|  | Report |  |  |  |  |  | Presentation on the Mid-term Evaluation of the Ten-Year Action Plan for Elderly Services 2016-2025[170] |
|  |  |  | Lobbying/promotion |  |  |  | The Secretary for Social and Cultural Affairs visited two elderly service facilities such as dementia and care and attention wards, which are subsidized by the Social Welfare Bureau[171] |
|  |  |  | Lobbying/promotion |  | Health Bureau |  | Representatives attended the Geriatrics Summit to introduce the dementia policy in Macau[172] |
|  |  |  | Service center activity/promotion |  |  |  | The Dementia Medical Center held a "Let's talk about dementia" event on World Alzheimer’s Day[173] |
|  | The use of dementia support center |  |  |  |  |  | The Dementia Support Center currently has over 360 participants in educational training programs[173] |
|  |  |  | Service center activity/promotion |  |  |  | The Geriatric Care Team of the General Health Care Subsystem sent health care workers to three community centers to hold a dementia awareness campaign[173] |
|  |  |  | Lobbying/promotion |  |  | Social Welfare Bureau, MADA and various organizations in Macau | The "Healthy Macau, Happiest Home - Building Dementia Friendly Communities" International Forum has expanded to 163 units to join the Dementia Friendly Communities Alliance in Macau[174] |
|  | The use of dementia medical center & dementia support center |  |  |  |  |  | Dementia Medical Center provided a total of 350 case consultations to dementia patients and their families and the number of participants in dementia education and training courses in the Dementia Support Center were 431 in 2019[170] |
|  | The use of dementia medical center |  |  |  |  |  | At the end of 2019, the cumulative number of new cases in the Dementia Medical Center were 1,560 and the cumulative number of cognitive function assessments were about 6,200, with a waiting time of one month[170] |
|  |  |  |  |  |  |  | Health centers held a total of 585 health education activities on dementia[175] |
|  | Work meeting |  |  |  | Comissão para os Assuntos do Cidadão Sénior |  | Plenary meetings (2019.03.06 & 2019.10.24)^,^ [176][177] |
|  | Work meeting |  |  |  | Chronic Disease Prevention and Control Committee |  | Work meeting (2019.03.08) [178] |
|  |  |  |  | International conference |  |  | A representative of the Dementia Working Group presented a poster at the 14th International Conference on Alzheimer's and Parkinson's Disease[179] |
|  | Work meeting |  |  |  | Dementia Work Group |  | 2 work meetings (2019.02.20 & 2019.05.08)[175] |
|  |  |  | Lobbying/promotion |  |  |  | 2019 Senior Living Products & Aids Exhibition & New Trends in Elderly Care Services Sharing Session[175] |
|  |  | Civil servant training |  |  |  |  | Continuous Public Health Training Courses[175] |
|  |  |  | Lobbying/promotion |  |  |  | Dementia workshop for members of Macau Cultural and Creative Arts Association[175] |
|  |  | Public training |  |  | MADA |  | Dementia Friendly Community Training Course 2019[180] |
|  |  |  |  | International conference |  |  | ADI Video Conference[180] |
|  |  |  | Lobbying/promotion |  |  | Macau Children Multi-intelligence Development Association | Rummikub contest[181] |
|  |  |  | Lobbying/promotion |  |  |  | Success in Life: A Life Sharing Session[175] |
|  |  |  |  | International conference |  |  | ADI Asia Pacific Members Meeting (Penang, Malaysia) [180] |
|  |  | Civil servant training |  |  | Kiang Wu Nursing College | Public Security Police Force, MADA | Co-organized Dementia Awareness Training Course[182] |
|  |  |  | Lobbying/promotion |  |  | MADA | Co-organized "Dementia Friendly Community Photo Contest[183] |
|  |  |  |  | Reviewed the development of healthcare services for elder adults |  |  | The development of health care services for older adults in Macao after Macao's return to China[184] |
|  |  |  |  | Summarized the impact of education and interdisciplinary collaboration on the dementia policy development |  |  | The impact of education and interdisciplinary collaboration on the Dementia policy development in Macao[184] |
|  |  |  |  | Shared the strategy, measures, experience in the prevention and treatment of dementia |  |  | Building Dementia friendly Macao by trans-institutes and multidisciplinary collaborations[184] |
|  |  |  |  | To deal with aging society |  |  | The establishment of nursing and health care curriculum in an ageing society[184] |
|  |  |  | Lobbying/promotion |  | Macau University of Science and Technology |  | Joined the Macao SAR Dementia Friendly Community Charter Program[185] |
|  |  |  |  | Drug screening, imaging, treatment evaluation | University of Macau | Shenzhen Institute of Advanced Technology, Chinese Academy of Sciences, Guangdong Medical University Hospital | Development of a "small molecule photoacoustic probe" for more effective assessment of dementia[186] |
|  |  |  |  | Investigating Macao citizens in dementia knowledge, attitudes towards people with dementia, and help-seeking behaviours and intention toward dementia | The Hong Kong Polytechnic University |  | Investigating dementia literacy among community-dwelling adults and older adults in Macau: A mixed methods study. 2019[187] |
|  |  | Patient training | Non-pharmacological therapy |  | Caritas Macau |  | Nursing Home "Sol Nascente" of Areia Preta Day Care adds Cognitive Stimulation Therapy (CST) training group[188] |
|  |  |  | Service center activity |  |  |  | Centro de Cuidados Especiais Longevidade Home Care and Support Services organized a trip to Zhuhai, Hong Kong for the "Joyful Tour" event[188] |
|  |  | Patient training | Non-pharmacological therapy |  |  |  | St. Francis Home for the Aged started non-pharmacological treatment groups for residents with dementia, such as art therapy groups[189] |
|  | Discussions and exchange of ideas in long-term care services for the elderly |  | Lobbying/promotion |  |  |  | The Liaison Office of the Central People's Government in the Macao SAR visited the nursing home to explore and exchange views[190] |
|  |  |  | Service center activity |  |  |  | Centro de Cuidados Especiais Longevidade organizes "Caring for the Elderly, Caring for the Brain - Walking with You" activities [191] |
|  |  |  | Lobbying/promotion |  |  |  | A special "Health Cheering Station" booth was set up at the Caritas fair to appeal to the public to pay more attention to dementia[192] |
|  |  |  | Lobbying/promotion |  |  |  | Launch of the "Macau: A Walk to Play in Chess" cognitive training kit[193] |
|  |  | Patient training | Non-pharmacological therapy |  |  |  | Centro de Dia" Brilho da Vida invited music therapists to the center to hold "Walk with You" music therapy group activities[192] |
|  |  |  | Lobbying/promotion |  | Macao Carers Association |  | Seminar on Dementia Policy in Macau[179] |
|  |  |  | Lobbying/promotion |  | APOMC |  | Hosted a seminar on Understanding Dementia[179] |
|  |  |  | Lobbying/promotion |  |  |  | "Dementia Prevention" Seminar[179] |
|  |  |  | Lobbying/promotion |  | Centro de Convívio Vivacidade da Associação Geral dos Operários de Macau | Conde S. Januário Hospital, Centro de Convivio "Hong Nin Chi Ka" da Associacao de Agricultores de Macau, Labour School Volunteers Association | Dementia Carnival - "Knowing Community Resources for Caring for the Elderly" was held[179] |
|  |  |  | Lobbying/promotion |  | Association of Friends of Charity of Macau |  | Dementia seminar with the theme of "Healthy Brain, Preventing Dementia"[194] |
|  |  |  | Non-pharmacological therapy |  | Macao Federation of Trade Unions |  | A community center used horticultural therapy to improve dementia for seniors[195] |
|  |  |  | Lobbying/promotion |  | Macau Physical Therapists Association |  | Signed a partnership with MADA to promote physiotherapy for dementia prevention to the [196] |
|  |  |  | Lobbying/promotion |  |  |  | Seminar on "Prevention of Dementia and Multi-disciplinary Collaboration[197] |
|  |  |  | Lobbying/promotion |  |  |  | Formed a dementia prevention group and prepared to launch the "Dementia Prevention Campaign Promotion Project"[197] |
|  |  |  | Lobbying/promotion |  | Macau Alumni Association of Chung Shan Medical University (Taiwan) |  | Seminar on the Role of Dementia Friendly Community Health Workers[179] |
|  |  |  | Lobbying/promotion |  | Macao Association of Speech Therapists |  | Public Lecture on Speech Therapy for Dementia and Stroke Patients[198] |
|  |  |  | Community outreach services |  | The Women' s General Association of Macau | Social Welfare Bureau | Two elderly centers participated in the Singleton Elderly Link Support Program. When volunteers found that the elderly living alone had mood changes and forgetfulness, they were suspected to have dementia and were referred to medical institutions for treatment after initial examination by social workers[199] |
|  |  | Caregivers’ training |  |  |  |  | Caregivers’ workshops & Elderly Care Group[175] |
|  |  | Staff training |  |  |  |  | Dementia Survey Staff Training[175] |
|  |  |  | Lobbying/promotion |  |  |  | Dementia prevention promotion activities[175] |
|  |  |  | Suspected dementia patients |  |  |  | Dementia at Kiang Wu Hospital Questionnaire[175] |
|  |  |  | Service center activity |  | Complexo de Serviços de Apoio ao Cidadão Senior “Pou Tai" |  | Sixth Anniversary Event - Dementia Service Sharing Session[200] |
|  |  |  | Service center activity/promotion |  | União Geral das Associações dos Moradores de Macau |  | Centro I Chon held "TANO Smart Rehabilitation Game Training Contest".[201] |
|  |  | HCP training | Non-pharmacological therapy |  |  |  | An Integrated Elderly Services Centre will extend its new Cognitive Stimulation Therapy (CST) training course to the community, training staff to provide CST training to the elderly with mild dementia[202] |
|  |  |  | Lobbying/promotion |  |  |  | A medical center held 4 dementia seminars[175] |
|  |  | HCP training | Non-pharmacological therapy |  |  | Macao Carers Association | The president of the Japan Recreational Therapy Association was invited to the center to give direct instruction to frontline colleagues on the use of doll therapy techniques[203] |
| 2020 |  |  | Lobbying/promotion |  | Social Welfare Bureau and Health Bureau |  | Macau Dementia Friendly Community Online Quiz Game[204] |
|  | Financial support |  |  |  | Social Welfare Bureau |  | Launch of the Caregiver Allowance Pilot Program[205] |
|  |  |  |  |  |  |  | 2020 annual Review Report of Macau Elderly Protection Mechanism and Ten-Year Action Plan for Elderly Services 2016-2025[206] |
|  | Organized an elderly integrated service center |  | Increase service facilities for elderly |  |  |  | Construction of an integrated elderly service center and an additional day center for the elderly[207] |
|  |  |  | Lobbying/promotion |  |  |  | Guidelines on prevention and management of lost elderly people with dementia - Family and Community[208] |
|  |  |  |  |  |  |  | Guidelines on Prevention and Management of Lost Elderly with Dementia - Social Service Units[209] |
|  | Organized a dementia integrated service center |  |  |  |  |  | Setting up the first Dementia Integrated Service Center[210] |
|  |  |  |  | Estimation of current dementia patient in Macao | Health Bureau |  | According to the data, there are about 4,000 people with dementia in 2018, and about 2,114 people were diagnosed and treated as of September 2018. The Health Bureau projects that there will be about 5,972 people with dementia in 2021; 7,771 people in 2026; 9,295 people in 2031; and 10,092 people in 2036.[211] |
|  | Recommendation on caring dementia patients during epidemic |  |  |  |  |  | Development of "Care for people with dementia: advice for caregivers and support groups during COVID-19” [211] |
|  | The use of dementia medical center and dementia support group |  |  |  |  |  | Dementia Medical Center provided a total of 386 consultations to dementia patients and their families & Dementia Support Center organized dementia education training courses for 72 participants and stimulation therapy groups for 108 participants [212] |
|  |  |  |  | Investigating primary health professionals in knowledge and attitudes and preventive practice of dementia care |  |  | Knowledge, attitude and preventive practice on dementia care among primary health professionals in Macao[213] |
|  |  |  |  | Investigating high school students in knowledge and attitudes and preventive practive of dementia care |  |  | High school students’ knowledge, attitude and preventive practice of dementia care in Macao.[214] |
|  |  |  | Lobbying/promotion |  |  |  | The dementia information page provided information on dementia education and training programs[212] |
|  | Work meeting |  |  |  | Dementia Work Group |  | Work meeting (2020.12.09) [212] |
|  |  |  |  | Understanding the effectiveness of promotional education efforts |  |  | A survey of senior service agency staff and seniors completed in 2020 and the results of the survey of local high school students published in the American Journal of Alzheimer's Disease & Other Dementias[212] |
|  |  |  | Lobbying/promotion | International conference |  |  | Delegates participated in the 34th International Conference of ADI Webinar. Presented as an oral presentation about Macao Dementia Policy as the 27th Globally: Challenges and Prospects and Knowledge, attitude, and preventive practice on dementia care among high school students in Macao showed with poster[212] |
|  |  | Civil servant training |  |  | Elderly Health Committee | Kiang Wu Nursing College | The annual work direction of "Community Friendly and Harmonious"; Kiang Wu Nursing College instructors were invited to conduct workshops for all members[215] |
|  |  | Civil servant training |  |  | Kiang Wu Nursing College |  | Ten training seminars on dementia for police officers[216] |
|  |  |  | Lobbying/promotion |  | Macau University of Science and Technology |  | Visit to the Dementia Support Center of the Macau Health Bureau with over 20 universities in China. [217] |
|  |  |  |  | Evaluating the effectiveness of the scale of attitudes toward people with dementia and their care |  |  | Reliability and Validity of the Chinese Version of the Dementia Attitude Scale[218] |
|  |  |  |  | New treatment strategy | University of Macau | Hong Kong Baptist University | Research team makes new discoveries about the pathogenesis of dementia[219] |
|  |  |  |  | Evaluating area-related differences in dementia literacy | The Hong Kong Polytechnic University |  | Dementia Literacy in the Greater Bay Area, China: Identifying the At-Risk Population and the Preferred Types of Mass Media for Receiving Dementia Information. [220] |
|  |  |  | Lobbying/promotion |  | MADA |  | 4th General Meeting and Special Lecture on dementia[180] |
|  |  |  | Lobbying/promotion |  |  |  | Coloring contest for elementary school students[180] |
|  |  |  | Lobbying/promotion |  |  |  | National Dementia Policy Learning Event[180] |
|  |  |  | Lobbying/promotion |  |  |  | Memory Walk activity[212] |
|  |  |  | Lobbying/promotion |  |  |  | Explore Alzheimer's Dementia Prevention and Treatment Services Work Plan Explanation[212] |
|  |  |  | Services - Support during COVID-19 |  | Caritas Macau |  | A day care worker provides information and remote care to the elderly in the epidemic through video, video clips and phone calls[221] |
|  |  | HCP training |  |  |  |  | Start Dementia Case Management Training Series[215] |
|  |  | Patient training | Non-pharmacological therapy |  |  |  | A day care service started the "I can do it" self-care group, using different life skills as the training theme[215] |
|  |  | Caregivers’ training |  |  |  | MGM Resorts International | Co-organized training course on dementia care[222] |
|  |  |  | Service center activity |  | Macao Federation of Trade Unions |  | Dementia prevention activities for dementia patients[212] |
|  |  |  | Lobbying/promotion |  |  |  | Online Quiz Competition[212] |
|  |  |  | Non-pharmacological therapy |  |  |  | Cognitive Training for suspected and confirmed dementia patients[212] |
|  |  |  | Lobbying/promotion |  |  |  | Rummikub contest[212] |
|  |  |  | New day care center – included dementia care services |  | União Geral das Associações dos Moradores de Macau |  | Opening of a Senior Center[223] |
|  |  |  | Lobbying/promotion |  |  |  | A medical center held 2 dementia seminars[212] |
|  |  |  | Lobbying/promotion |  | Associação dos Cidadãos Unidos de Macau |  | The Medical Center of People holds monthly health talks for the public on a variety of topics, including dementia prevention and health care. [224] |
|  |  |  | Lobbying/promotion |  | The Women' s General Association of Macau |  | Dementia Prevention Promotion Activities[212] |
|  |  |  | Lobbying/promotion |  |  |  | Dementia prevention seminar[212] |
|  |  | Caregivers’ training | Lobbying/promotion |  | Macau Association of the Hearing Impaired |  | Dementia-friendly community seminars and workshops for seniors, caregivers, and staff with hearing impairment to provide basic understanding and prevention of dementia, caregiver stress and support[225] |
| 2021 |  | Public training |  |  | Social Welfare Bureau & Health Bureau |  | Co-organized 4 Dementia Friendly Alliance online training sessions[226] |
|  |  |  | Lobbying/promotion |  | Health Bureau | Social Welfare Bureau & Chronic Disease Prevention and Control Committee | Brain Exercise Promotion Activities[227] |
|  |  |  |  |  |  |  | Dementia Medical Center: A total of 500 participants in dementia education training courses, 226 participants in group health education, and 533 participants in cognitive groups[228] |
|  |  |  |  | Introducing the need and implementation of dementia policy |  |  | Macao Dementia Policy: Challenges and prospects (innovative practice) [229] |
|  | Work meeting |  |  |  | Comissão para os Assuntos do Cidadão Sénior |  | Plenary Meeting (2021.04.28) Presentation of the results and recommendations of the "Survey on the Awareness and Attitudes of Macau People towards Dementia[230] |
|  | Work meeting |  |  |  | Chronic Disease Prevention and Control Committee |  | Work meeting (2021.07.27) [231] |
|  | Work meeting |  |  |  | Dementia Work Group |  | Work meeting (2021.02.25) [228] |
|  |  |  | Lobbying/promotion |  |  |  | Introduced dementia information and the work of the Health Bureau through various media such as newspapers and television[228] |
|  | Optimization of dementia registry |  |  |  | Preparation of research (local data) |  | To optimize the information in the internal dementia database of the Health Bureau for collation and preparation for the future publishment of local dementia data[228] |
|  |  |  | Interrogatory |  | Legislator Si Ka Lon |  | Written question on support for dementia services and publicity and education in Macao[232][233] |
|  |  |  | Lobbying/promotion |  | MADA |  | Dementia education at school (The International School of Macao) written inquiry & received feedback from the government[234] |
|  |  |  | Lobbying/promotion |  | Elderly Health Committee |  | Planned and Filmed for Dementia - "Early Diagnosis and Early Treatment"[235] |
|  |  | HCP training |  |  | Mother Mary Social Studies Centre |  | Therapist Assistant Training Certificate Program[236] |
|  |  |  |  | Result of dementia research | Kiang Wu Nursing College |  | The results of the survey on dementia awareness and attitudes in Macau show that respondents have a basic understanding of dementia and a weak ability to identify symptoms, often misunderstand dementia as part of normal aging, and still have a "paternalistic" view of dementia care, such as dementia patients need to be taken care of like children. [237] |
|  |  | Caregivers’ training | Lobbying/promotion |  |  |  | General Medical Seminar: Golden Age Eating and Dementia Feeding Tips [238] |
|  |  |  | Lobbying/promotion | Caregivers’ experience sharing; online training course for caregiver |  | MADA | Dementia Family Caregiver Experience Sharing Session for Research - Learning iSupport Platform for Dementia Caregivers in Macau: A Collaborative Study in Australia and the Greater China Region with International Collaborative Research Project[239] |
|  |  |  | Lobbying/promotion |  |  |  | Seminar on Dementia Care Research and Practice[240] |
|  |  |  |  | Estimating the prevalence and incidence of dementia in Macao and analyzing its pattern of development for policy development |  |  | Analysis of the dementia population and their development trend in Macao[241] |
|  |  |  |  | The need of strengthening early detection and diagnosis of dementia and selecting the most suitable screening tools |  |  | Reflections on a dementia screening program for the elderly in Macao community.[242] |
|  |  | Civil servant training |  |  |  |  | Conducted 8 dementia training sessions for police officers[243] |
|  |  |  |  | Association between cognitive dysfunction patients and TCM physique | University of Macau |  | Cognitive dysfunction of elderly in Macau found to be associated with TCM constitution[244] |
|  |  | Caregivers’ training |  |  | Caritas Macau |  | Caritas Home Care and Caregiver Support Service offered training courses for caregivers [245] |
|  |  | Patient training | Non-pharmacological therapy |  |  |  | A cognitive training group for dementia residents in a residential care home[246] |
|  |  |  | Service center activity |  |  |  | A senior center held a group gathering for caregivers and friends on the eve of Mid-Autumn Festival[246] |
|  |  |  | Caregiver support |  |  |  | An elderly integrated service center held an elderly care group[247] |
|  |  | HCP training |  |  |  |  | Social Worker Training for Elderly Services - Elderly Services Redevelopment Training Series (Online Course) including Dementia Intervention to Reduce Restrain[247] |
|  |  |  | Non-pharmacological therapy |  |  |  | A day care center for the elderly designed a chess game for cognitive training[247] |
|  |  |  | Activity; promotion |  |  | MGM Resorts International | Set up a tour[248] |
|  |  |  | Lobbying/promotion |  |  |  | Seminar on Innovation and Challenges[249] |
|  |  |  | Lobbying/promotion |  | MGM Resorts International | Caritas Macau | Develop dementia care program [250] |
|  |  |  | Lobbying/promotion |  |  |  | Sponsored Kiang Wu Nursing College to produce 3,000 copies of "Dementia Risk Reduction" promotional and educational booklet[250] |
|  |  |  | Lobbying/promotion |  | União Geral das Associações dos Moradores de Macau |  | 2021 Seminar on Mental Health for the Elderly in the Community in Mainland China, Hong Kong and Macau[251] |
|  |  |  | Lobbying/promotion |  |  |  | Dementia prevention game contest for the elderly in an elderly integrated center[252] |
|  |  |  | Lobbying/promotion |  |  |  | A medical center held 5 seminars for the public[228] |
|  |  |  | Lobbying/promotion |  | Sands China Ltd |  | Volunteers Celebrate Mid-Autumn with the Elderly with Dementia[253] |
|  |  |  |  | Effectiveness of non-pharmacotherapy(horticultural therapy) | Macau Horticultural Therapy Association | Complexo de Serviços de Apoio ao Cidadão Senior “Pou Tai" | Published research results on the effectiveness of horticultural therapy and cognitive impairment in the treatment of elderly with mild to moderate cognitive impairment in institutions[254] |
|  |  |  | Lobbying/promotion |  | Macao Community Voluntary Services Association |  | Promotional video on dementia care[255] |
|  |  | Caregivers’ training | Lobbying/promotion |  | Macao Federation of Trade Unions |  | 7 dementia prevention activities; 1 Cognitive Accessibility Seminar and 1 caregivers’ training activity[228] |
|  |  |  | Service center activity; promotion |  | The Women' s General Association of Macau |  | A community center held a three-month dementia prevention promotion series[256] |
|  |  |  | Lobbying/promotion |  |  |  | 2 dementia prevention & awareness activities[228] |
|  |  |  | Lobbying/promotion |  |  |  | Preventing Dementia - Psychological Changes in the Elderly for fishermen[228] |
| 2022 |  |  | Lobbying/promotion |  | Social Welfare Bureau & Health Bureau |  | The Secretary for Social Affairs and Culture visited the services and facilities of the Integrated Services Centre for the Elderly to understand the living conditions of the elderly with dementia and the elderly in general [257] |
|  | Dementia-specific one-stop service |  |  |  |  |  | Set up the first integrated dementia service center with 100 day care and residential service places in the first phase [258] |
|  |  |  | Lobbying/promotion |  |  |  | Organized online quiz games[259] |
|  | Financial support |  |  |  | Social Welfare Bureau |  | Social Inclusion Program - The program benefits families who are economically disadvantaged or near-poor. Eligible diseases or disabilities include cerebrovascular disease, Parkinson's, AIDS, epilepsy, dementia and autism. [260] |
|  | Increase health services for elderlies (service approach) |  |  |  |  |  | Will launch a pilot remote vision clinic in elderly homes and assistive services for caregivers of seniors with dementia in 2023[261] |
|  |  |  | Lobbying/promotion |  | Health Bureau |  | Conde S. Januário Hospital Specialist Health Education Seminar on Dementia Home Care for the Elderly[262] |
|  |  |  |  | Dementia registry aims to better understanding of health service use, medicines, hospitalizations, mortality |  |  | Cohort Profile: The Dementia Registry in Macao[263] |
|  | Work meeting |  |  |  | Comissão para os Assuntos do Cidadão Sénior |  | Plenary meeting (2022.09.20) [264] |
|  | Work meeting |  |  |  | Chronic Disease Prevention and Control Committee |  | Work Session (2022.11.08) [265] |
|  |  |  | Lobbying/promotion |  |  | Associação dos Familiares Encarregados dos Deficientes Mentais de Macau | Dementia Work Group Holds Dementia Prevention Seminar[266] |
|  |  | HCP training |  |  | Kiang Wu Nursing College |  | ADI Certified Institute's Dementia Care Manager Program and Dementia Caregiver Program[267] |
|  |  |  |  | Questionnaires about knowledge, attitudes among occupation practitioners |  |  | The Willingness to Help People With Dementia Symptoms Among Four Occupation Practitioners in Macao [268] |
|  |  |  |  | Investigating age-related differences in dementia knowledge and attitudes |  |  | Comparisons of Dementia Knowledge and Attitudes among the Youth and Older Adults: Insights from the Construal Level Theory Perspective[269] |
|  |  |  |  | Related to yin deficiency and qi depression |  |  | To formulate Traditional Chinese Medicine constitution differentiation nursing for older people with cognitive impairment in Macau based on constitutional identification theory[270] |
|  |  |  | Lobbying/promotion |  | Seniors College |  | Health Talk on Understanding Dementia for the Elderly [271] |
|  |  |  |  | Experience in post-diagnosis dementia | MADA |  | ADI 2022 Dementia Topical Large-Scale Survey Study[272] |
|  |  |  | Lobbying/promotion |  | Centro de Serviços de Tele-Assistência “Peng On Tung” de Macau |  | Brain Fitness Finger Exercise Video[273] |
|  |  |  | New service center for elderly |  | Macau Association of Jiangmen Youths | Social Welfare Bureau | New activity center opens [274] |
|  |  |  | Hotline service |  | Macao Carers Association |  | Set up a voice service hotline to provide residents with a variety of voice tests, such as the Very Early stage of Dementia Test[275] |
|  |  |  | Lobbying/promotion |  | Macau Electricity Company, Ltd | Macao Youth Federation, Macao Federation of Trade Unions | Rummikub Contest[276] |
|  |  |  |  |  | Providing the practice standard for developing a dementia-friendly society in China via expert consensus; public promotion |  | Chinese expert consensus on the development of dementia-friendly communities in Guangdong-Hong Kong-Macao Greater Bay Area[277] |
|  |  |  |  |  | Macao Federation of Trade Unions | University of Saint Joseph | Signing of Memorandum of Cooperation to prevent dementia[278] |
|  |  |  | Service center activities; promotion |  |  | Social Welfare Bureau | Senior Services Center Hosts Prevention Education Series[279] |
| 2023 |  |  |  | Chinese instruments examined psychometrically to integrate knowledge, attitude, and preventive behavior in dementia care | Health Bureau |  | Development and Psychometric Evaluation of a Chinese Instrument of Knowledge, Attitude and Preventive Practice on Dementia Care in Macao[280] |
|  |  |  | Lobbying/promotion |  | MGM Resorts International |  | Led the Golden Lion Volunteer Team to visit five elderly centers and provided the "Reducing the Risk of Dementia" educational brochure to elderlies[281] |
|  | Policy suggestion in early detection |  |  |  | General Association of Chinese Students of Macau |  | Secretary suggested the government can take the initiative to provide appropriate dementia screening for the elderly in adult health clinics. Also, the secretary reflected that awareness of dementia and its patients is still lacking[282] |
|  |  |  | Lobbying/promotion |  | Exmoo news |  | Studies have found that people with blood sodium levels above 142 mmol/L are at increased risk for chronic diseases such as heart failure, stroke, chronic lung disease, diabetes and dementia. [283] |
| Training material |  | Training material |  |  | Health Bureau |  | Dementia Support Center  Elderly and Caregiver Education Training Program[284] |
|  |  | Training material |  |  |  |  | Dementia Medication[284] |
|  |  | Training material |  |  |  |  | Nutritional Diet Guidelines for Dementia Patients[284] |
|  |  | Training material |  |  |  |  | Dementia Home Care and Communication Skills[284] |
|  |  | Training material |  |  |  |  | Maintenance of home safety and daily self-care activities[284] |
|  |  | Training material |  |  |  |  | Mental behavior symptoms and cognitive activities at home[284] |
|  |  | Training material |  |  |  |  | Common Psychiatric Symptoms of Dementia[284] |
|  |  | Training material |  |  |  |  | Emotional Crisis and Adaptation for Dementia Caregivers[284] |
|  |  | Training material |  |  |  |  | Exercise Training[284] |
|  |  | Training material |  |  |  |  | Cognitive stimulation therapy[284] |
|  |  | Training material |  |  |  |  | Reminiscence Therapy[284] |
|  |  | Training material |  |  |  |  | Introduction of Dementia Community Support Service in Macau[284] |
|  |  | Training material |  |  |  |  | Personal Care Planning and Hospice Care[284] |
|  |  | Training material |  |  |  |  | Dementia Friendly Alliance Macau Training Workshop[284] |

**Reference (2)**

1. Macao Yearbook 2009. Macao Yearbook. https://yearbook.gcs.gov.mo/uploads/yearbook_pdf/2009/myb2009c.pdf. Accessed January 12, 2023.
2. Leong HT. Interrogatory. Macao Legislative Council Written Question Website. https://www.al.gov.mo/uploads/attachment/written-consultation/2009/29535585ff03c4f528.pdf. Published 2009. Accessed January 13, 2023.

Tam CW. Replies to interrogatory raised by LEONG Heng Teng. Macao Legislative Council Written Question Website. https://www.al.gov.mo/uploads/attachment/written-consultation/2009/59723585ff04180f90.pdf. Accessed January 13, 2023.

1. Elderly Affairs Committee visits Beijing and Shanghai to study elderly services. NEWS GOV-MO. October 14, 2010. https://www.gcs.gov.mo/detail/zh-hant/N10JNkhg88?. Accessed January 13, 2023.
2. Leung KLN, Sam WL, Leong ML, Choi IH, Lei WI, Lai KYC. An analysis of the development of dementia care services in Macao : policies and resources allocation. Chinese journal of nursing. 2010;45(1):50-52. doi:10.3761/j.issn.0254-1769.2010.01.020
3. Completed research project 11: Community screening of elderly dementia patients in the early stages of dementia in Macau. Kiang Wu Nursing College of Macau website. http://www2.kwnc.edu.mo/?page_id=2038. Accessed January 13, 2023.
4. Macao Alzheimer's Disease Association Organizing Committee was established. Jornal Cheng Pou. September 21, 2010. http://www.chengpou.com.mo/dailynews/147438.html. Accessed January 13, 2023.

The government is actively improving the care services for the elderly. NEWS GOV-MO. January 26, 2011. https://www.gcs.gov.mo/detail/zh-hant/N11AZPLW2u?. Accessed January 13, 2023.

1. Social Welfare Bureau’s Services and Future Development for the Elderly with Dementia. GOV.MO. January 19, 2011. https://www.gov.mo/zh-hant/news/93647/. Accessed January 13, 2023.

Social Welfare Bureau's service initiatives for the elderly. NEWS GOV-MO. January 22, 2011. <https://www.gcs.gov.mo/detail/zh-hant/N11AUy92pU>?. Accessed January 13, 2023.

1. Specialized Health Education Seminar. NEWS GOV-MO. June 23, 2011. https://www.gcs.gov.mo/detail/zh-hant/N11FWn0Wux?. Accessed January 13, 2023.

CHCSJ September 2011 Specialist Health Education Seminar. NEWS GOV-MO. August 17, 2011. https://www.gcs.gov.mo/detail/zh-hant/N11HQdXcuZ?. Accessed January 14, 2023.

CHCSJ October 2011 Specialist Health Education Seminar. NEWS GOV-MO. September 22, 2011. https://www.gcs.gov.mo/detail/zh-hant/N11IVU0pbJ?. Accessed January 14, 2023.

CHCSJ December 2011 Specialist Health Education Seminar. NEWS GOV-MO. November 22, 2011. https://www.gcs.gov.mo/detail/zh-hant/N11KV17nTq?. Accessed January 14, 2023.

Lei CI. Replies to interrogatory raised by ZHENG Anting. https://www.al.gov.mo/uploads/attachment/written-consultation/2014/803065861e71d4a42f.pdf. Accessed January 14, 2023.

Benevolence Lights up my Later Life. Benevolence Lights up my Later Life Facebook website. https://www.facebook.com/kwnc.baba. Updated January 2012. Accessed January 15, 2023.

Completed research project 09: Cross-Site Comparison of Dementia Family Caregiver Burdens. Kiang Wu Nursing College of Macau website. http://www2.kwnc.edu.mo/?page_id=2038&doing_wp_cron=1677903097.5310330390930175781250. Accessed January 14, 2023.

Macau Alzheimer’s Association to join international organization. Jornal San Wa Ou. January 31, 2013. https://www.waou.com.mo/2013/01/31/%E6%BE%B3%E9%96%80%E5%A4%B1%E6%99%BA%E7%97%87%E5%8D%94%E6%9C%83%E5%B0%87%E5%8A%A0%E5%85%A5%E5%9C%8B%E9%9A%9B%E7%B5%84%E7%B9%94/. Accessed January 14, 2023.

Background. Macao SAR Senior Citizen Service Information Website. http://www.ageing.ias.gov.mo/consult/intro. Accessed January 13, 2023.

CHCSJ February 2012 Specialist Health Education Seminar. NEWS GOV-MO. January 18, 2012. https://www.gcs.gov.mo/detail/zh-hant/N12ARQDmrp?. Accessed January 14, 2023.

Health Bureau says establishing a healthy lifestyle can help prevent Alzheimer's disease (also known as "dementia in the elderly"). NEWS GOV-MO. March 26, 2012. https://www.gcs.gov.mo/detail/zh-hant/N12CZB4x8a?. Accessed January 14, 2023.

The first plenary session of 2012. Social Welfare Bureau: Macao SAR website. https://www.ias.gov.mo/wp-content/themes/ias/tw/sservcomms/2_eldcomm/eldcomm_b_con_01/eldcomm_b_con_meet1203.htm. Accessed January 14, 2023.

Elderly Affairs Committee discusses the issue of ageing in Macao. NEWS GOV-MO. September 27, 2012. https://www.gcs.gov.mo/detail/zh-hant/N12IayUXtO?. Accessed January 14, 2023.

Chan IT, Wu HM, Luk L. A health promotion program to reduce the care burden of five dementia caregivers. Macau Journal of Nursing. 2012;11(2): 56-58.

Pre-allocation of 336 units in two-bedroom blocks in Edifício Fai Ieng. NEWS GOV-MO. October 15, 2013. https://www.gcs.gov.mo/detail/zh-hant/N13JOGJOpy?. Accessed January 14, 2023.

Maintain the minimum subsistence index level. GOV.MO. June 22, 2013. https://www.gov.mo/zh-hant/news/106599/. Accessed January 14, 2023.

"World Mental Health Day" series calls for attention to elderly and mental health. NEWS GOV-MO. October 9, 2013. https://www.gcs.gov.mo/detail/zh-hant/N13JIfdqmh?. Accessed January 14, 2023.

The first plenary session of 2013. Social Welfare Bureau: Macao SAR website. http://www.ias.gov.mo/archives/gonggaotongzhi/%E9%95%B7%E8%80%85%E4%BA%8B%E5%8B%99%E5%A7%94%E5%93%A1%E6%9C%832013%E5%B9%B4%E7%AC%AC%E4%B8%80%E6%AC%A1%E6%9C%83%E8%AD%B0. Accessed January 14, 2023.

Dementia Community Service Action Research Project - Creating a Memory Center. Kiang Wu Nursing College of Macau website. http://www2.kwnc.edu.mo/?page_id=2006. Accessed January 14, 2023.

Completed research project 02: Dementia in the Elderly Community Action Research Project - Creating a Dementia Telephone Service Hotline. Kiang Wu Nursing College of Macau website. http://www2.kwnc.edu.mo/?page_id=2002. Accessed January 14, 2023.

Ho J, Mathews RM, Heard R, Chow CM. Differences in sleep of dementia residents between Macao (China) and Sydney (Australia). Macau Journal of Nursing. 2013;12(2): 52-57.

Dementia Community Service Action Research Project on Enhancing Community Support Services and Training Facilities for the Elderly with Dementia. Kiang Wu Nursing College of Macau website. http://www2.kwnc.edu.mo/?page_id=1974. Accessed January 14, 2023.

The 16th International Dementia Association Asia Pacific Symposium. Kiang Wu Nursing College of Macau website. http://www2.kwnc.edu.mo/?page_id=6783. Accessed January 14, 2023.

Association organizes International Dementia Month awareness campaign. Jornal Cheng Pou. September 11, 2013. http://www.chengpou.com.mo/dailynews/173135.html. Accessed January 14, 2023.

1. Caritas Newsletter 125. Caritas Macau website. https://www.caritas.org.mo/uploads/ueditor/file/20190729/1564374082398059.pdf. Accessed January 13, 2023.
2. Research Report on the Situation and Policies of the Elderly in Macao. Macau Senior Citizen Service Information Website. http://www.ageing.ias.gov.mo/uploads/file/a8387e568125adacb3c4d280d5354985.pdf. Accessed January 13, 2023.

Mr. Tam Chon Weng, the Chief Secretary for Social Affairs and Culture, chaired the first meeting of the Elderly Commission in 2016. NEWS GOV-MO. April 8, 2016. https://www.gcs.gov.mo/archive/showNews.php?PageLang=C&DataUcn=98587. Accessed January 16, 2023.

Lei CI. Replies to interrogatory raised by WONG Kit Cheng. Macao Legislative Council Written Question Website. https://www.al.gov.mo/uploads/attachment/written-consultation/2014/676785861e4ca07395.pdf. Accessed January 14, 2023.

CHCSJ September 2014 Specialist Health Education Seminar. NEWS GOV-MO. August 18, 2014. https://www.gcs.gov.mo/detail/zh-hant/N14HRBLhpf?. Accessed January 14, 2023.

Zheng A. Interrogatory on measures to promote the development of care for patients with Alzheimer's disease. Macao Legislative Council Written Question Website. https://www.al.gov.mo/uploads/attachment/written-consultation/2014/677855861e71cc447c.pdf. Accessed January 14, 2023.

Wong KC. Interrogatory: focusing on dementia prevention in Macao. Macao Legislative Council Written Question Website. https://www.al.gov.mo/uploads/attachment/written-consultation/2014/626955861e4c99223c.pdf. Accessed January 14, 2023.

Kiang Wu Nursing College teaches brain-awakening activities. Jornal Cheng Pou. June 11, 2014. http://www.chengpou.com.mo/dailynews/96432.html. Accessed January 16, 2023.

Women's Commission Visits Complexo de Serviços de Apoio ao Cidadão Senior “Pou Tai”. NEWS GOV-MO. July 23, 2014. https://www.gcs.gov.mo/detail/zh-hant/N14GWwIumD?. Accessed January 16, 2023.

1. Caritas Macau Work Report 2014. Caritas Macau website. https://www.caritas.org.mo/uploads/ueditor/file/20190729/1564368217287761.pdf. Accessed January 13, 2023.

International Policy and Theory Research Report on Aging. Macau Senior Citizen Service Information Website. https://www.ageing.ias.gov.mo/uploads/file/484ba397e54612c2cfe7cc567f776353.pdf. Accessed January 13, 2023.

Seminar on dementia care and safe and rational use of medication. Macao dementia friendly community website. https://www.ssm.gov.mo/apps1/mdfc/ch.aspx#clg12684. Accessed January 13, 2023.

Ten-year action plan for the elderly in Macau (2016-2025): public consultation. Macau Senior Citizen Service Information Website. https://www.ageing.ias.gov.mo/uploads/file/c7f6c91bae1933b4544cbd0e73788f2d.pdf. Accessed January 13, 2023.

Work Report 2015. Social Welfare Bureau website. http://www.ias.gov.mo/wp-content/uploads/file/ias_report_2015.pdf. Accessed January 13, 2023.

Mr. Tam Chon Weng, the Chief Secretary for Social Affairs and Culture, inspected the psychiatric building and infectious disease facilities of the Health Bureau. GOV.MO. October 9, 2015. https://www.gcs.gov.mo/detail/zh-hant/N15JIjXouF?. Accessed January 16, 2023.

Mr. Tam Chon Weng, the Chief Secretary for Social Affairs and Culture, visited the Elderly and Youth Service Center. GOV.MO. November 10, 2015. https://www.gcs.gov.mo/detail/zh-hant/N15KJRhG1I?. Accessed January 16, 2023.

Benevolence Lights up my Later Life Youth Ambassador honored at International Senior Day. CyberCTM. December 5, 2015. https://www.cyberctm.com/zh_TW/news/detail/1087939#.ZAaZp-tBy3J. Accessed January 16, 2023.

CHCSJ August 2015 Specialist Health Education Seminar. NEWS GOV-MO. July 17, 2015. https://www.gcs.gov.mo/detail/zh-hant/N15GQ71dSg?. Accessed January 14, 2023.

First meeting of the Elderly Affairs Commission in 2015. NEWS GOV-MO. March 4, 2015. https://www.gcs.gov.mo/detail/zh-hant/N15CDMnHAd?. Accessed January 16, 2023.

Chan MI. Interrogatory: prevention and treatment of dementia in the elderly. Macao Legislative Council Written Question Website. https://www.al.gov.mo/uploads/attachment/written-consultation/2015/34281586124727be02.pdf. Accessed January 14, 2023

Lei CI. Replies to interrogatory raised by CHAN Mei I. Macao Legislative Council Written Question Website. https://www.al.gov.mo/uploads/attachment/written-consultation/2015/5169858612472e4af0.pdf. Accessed January 14, 2023.

Memory Clinic is available in our hospital. Kiang Wu Hospital website. http://www.kwh.org.mo/news%20info.php?nid=1430#:~:text=%E5%9B%A0%E6%87%89%E7%A4%BE%E6%9C%83%E7%99%BC%E5%B1%95%E9%9C%80%E8%A6%81%EF%BC%8C%E6%9C%AC,%E6%97%A9%E6%9C%9F%E7%99%BC%E7%8F%BE%E3%80%81%E6%97%A9%E6%9C%9F%E6%B2%BB%E7%99%82%E7%9B%AE%E7%9A%84%E3%80%82. Accessed January 14, 2023.

Directors of elderly services were briefed on the development of dementia medical and social services. NEWS GOV-MO. September 20, 2016. https://www.gcs.gov.mo/detail/zh-hant/N16ITfQTL2?. Accessed January 14, 2023.

Ten-year action plan for the elderly in Macau (2016-2025): 2016 Annual Review Report. Macau Senior Citizen Service Information Website. https://www.ageing.ias.gov.mo/uploads/file/84b354b3c71ccddde2b664b302ac8c64.pdf. Accessed January 14, 2023.

The Chief Secretary for Social Affairs and Culture officiated at the opening ceremony of the Dementia Medical Center. NEWS GOV-MO. September 21, 2016. https://www.gcs.gov.mo/detail/zh-hant/N16IUA9Eah?. Accessed January 14, 2023.

Dementia patients increasing: Health Bureau calls for early prevention and treatment. Exmoo News. October 5, 2016. https://www.exmoo.com/article/19210.html. Accessed January 14, 2023.

Work Report 2016. Social Welfare Bureau website. http://www.ias.gov.mo/wp-content/uploads/file/ias_report_2016.pdf. Accessed January 13, 2023.

Ten-year action plan for the elderly in Macau (2016-2025). Macau Senior Citizen Service Information Website. https://www.ageing.ias.gov.mo/uploads/file/20160408e.pdf. Accessed January 14, 2023.

Ten-year action plan for the elderly in Macau (2016-2025): promotion leaflet. Macau Senior Citizen Service Information Website. https://www.ageing.ias.gov.mo/uploads/file/20160408b.pdf. Accessed January 14, 2023.

Ten-year action plan for the elderly in Macau (2016-2025): checklist. Macau Senior Citizen Service Information Website. http://www.ageing.ias.gov.mo/uploads/file/2b610632d0ce7f17630cdad5abacaca5.pdf. Accessed January 14, 2023.

Ten-year action plan for the elderly in Macau (2016-2025): Executive List. Macau Senior Citizen Service Information Website. http://www.ageing.ias.gov.mo/uploads/file/9cd4e55932a3825eafbf49005fe6b048.pdf. Accessed January 14, 2023.

Secretary for Social Affairs and Culture Visits Hong Fai Day Care Center for the Elderly. GOV.MO. September 21, 2016. https://www.gov.mo/zh-hant/news/170373/. Accessed January 14, 2023.

Understanding Dementia. Kiang Wu Nursing College of Macau website. http://www2.kwnc.edu.mo/?page_id=17193. Accessed January 16, 2023.

Increase resources to strengthen dementia services. Exmoo News. September 19, 2016. https://www.exmoo.com/article/18281.html. Accessed January 14, 2023.

Chronic Disease Prevention and Control Committee Holds Second Work Session of 2016. NEWS GOV-MO. October 25, 2016. https://www.gcs.gov.mo/archive/showNews.php?PageLang=C&DataUcn=105331. Accessed January 14, 2023.

Certificate in Cognitive Function Assessment Course Completion. Macao Daily News. January 16, 2017. https://www.ssm.gov.mo/docs/12937/12937_7ee5a02adb7a496f87acbbf46aa92cb1_000.pdf. Accessed January 16, 2023.

About Ten-year action plan for the elderly in Macau (2016-2025 and Macao SAR Senior Citizens Day. Education and Youth Affairs Bureau website. https://www.dsedj.gov.mo/cep/lifelong_learning/36/10-13.pdf. Accessed January 17, 2023.

Secretary for Social Affairs and Culture chaired the first meeting of the Elderly Commission in 2016. NEWS GOV-MO. April 8, 2016. https://www.gcs.gov.mo/detail/zh-hant/N16DH3aLld?. Accessed January 14, 2023.

Secretary for Social Affairs and Culture chaired the 2nd meeting of the Elderly Commission in 2016. GOV.MO. October 18, 2016. https://www.gov.mo/zh-hant/news/168177/. Accessed January 14, 2023.

Elderly Affairs Committee visited the Dementia Medical Center. NEWS GOV-MO. November 10, 2016. https://www.gcs.gov.mo/detail/zh-hant/N16KJWmvZk?. Accessed January 14, 2023.

Chronic Disease Prevention and Control Committee Holds Second Work Session of 2016. GOV.MO. October 25, 2016. https://www.gov.mo/zh-hant/news/167440/. Accessed January 14, 2023.

Ho IS. Interrogatory: publicity and education on dementia, care services and assessment standards. Macao Legislative Council Written Question Website. https://www.al.gov.mo/uploads/attachment/written-consultation/2016/556585861112a99999.pdf. Accessed January 10, 2023.

Cheang SI. Replies to interrogatory raised by HO Ion Sang. Macao Legislative Council Written Question Website. https://www.al.gov.mo/uploads/attachment/2017-03/2982558c7d24b5084e.pdf. Accessed January 14, 2023.

Teachers and students reflect on the results of "Life Education" activities: Kiang Wu Nursing College monthly meeting to spread dementia knowledge. Exmoo News. March 18, 2016. https://www.exmoo.com/article/5884.html. Accessed January 18, 2023.

Benevolence Lights up my Later Life Project Addresses Dementia in Aging: Kiang Wu Nursing College Promotes Intergenerational Inclusion. Exmoo News. November 3, 2016. https://www.exmoo.com/article/21041.html. Accessed January 18, 2023.

Memory Center. Kiang Wu Nursing College of Macau website. http://www2.kwnc.edu.mo/?page_id=854. Accessed January 18, 2023.

Zheng W, & Van IK. Analysis of Early-Stage Dementia Screening among Elderly Population in Macao's Community. Journal of Nursing Management. 2016;(5): 339-340

Hu SXX, Lei WI, Chao KK, Hall BJ, Chung SF. Common chronic health problems and life satisfaction among Macau elderly people. International Journal of Nursing Sciences. 2016;3(4):367-370. doi:10.1016/j.ijnss.2016.10.004

Dementia prevention and control: Macao Alzheimer’s Disease Association Appeal. Exmoo News. January 13, 2016. https://www.exmoo.com/article/2132.html. Accessed January 13, 2023.

Report on Macau's Status, Take Action: Kiang Wu Nursing College Participates in International Dementia Conference. Exmoo News. May 12, 2016. https://www.exmoo.com/article/9573.html. Accessed January 13, 2023.

1. Caritas Macau Work Report 2016. Caritas Macau website. https://www.caritas.org.mo/uploads/ueditor/file/20190729/1564368091326684.pdf. Accessed January 13, 2023.

Visit to Kiang Wu Nursing College and CHCSJ for better understanding: Macao Carers Association is concerned about dementia assessment and treatment. Exmoo News. November 9, 2016. https://www.exmoo.com/article/21446.html. Accessed January 13, 2023.

Triggering public attention to the aging trend of society: Premiere of locally made dementia-themed film. Exmoo News. November 20, 2016. https://www.exmoo.com/article/22159.html. Accessed January 15, 2023.

Exercising the mental health of the elderly to prevent dementia: UGAMM to launch "Toy Library" next month. Exmoo News. February 9, 2016. https://www.exmoo.com/article/3343.html. Accessed January 15, 2023.

Work Report 2017. Social Welfare Bureau website. http://www.ias.gov.mo/wp-content/uploads/file/ias_report_2017.pdf. Accessed January 13, 2023.

Ten-year action plan for the elderly in Macau (2016-2025): 2017 Annual Review Report. Macau Senior Citizen Service Information Website. http://www.ageing.ias.gov.mo/uploads/file/840e2cd1bb19b11c516c25120642d320.pdf. Accessed January 14, 2023.

Secretary for Social Affairs and Culture's visit to Lisbon: Macau-Portugal Medical Cooperation Promoted. Exmoo News. September 21, 2017. https://www.exmoo.com/article/40933.html. Accessed January 17, 2023.

Interdepartmental Steering Group on Pension Protection Mechanism Holds First Plenary Meeting in 2017. GOV.MO. March 24, 2017. https://www.gov.mo/zh-hant/news/181949/. Accessed January 14, 2023.

Secretary for Social and Cultural Affairs meets representatives of the social services sector to hear policy proposals. GOV.MO. October 10, 2017. https://www.gov.mo/zh-hant/news/208282/. Accessed January 18, 2023.

Forum on "Healthy Macau, Happiness Home - Building a Dementia Friendly Community in Macau" was successfully held. NEWS GOV-MO. October 22, 2017. https://www.gcs.gov.mo/detail/zh-hant/N17JVhOBFu?. Accessed January 15, 2023.

Legislators are concerned about the problems of aging and dementia: Dementia Call Service for the Elderly to be launched next year. Exmoo News. December 1, 2017. https://www.exmoo.com/article/46876.html. Accessed January 15, 2023.

Caring for the physical and mental health of the elderly: Establishing a comprehensive retirement protection system. Exmoo News. December 19, 2017. https://www.exmoo.com/article/48710.html. Accessed January 16, 2023.

Successful completion of two "Certificate in Cognitive Functioning Assessment" courses. GOV.MO. January 15, 2017. https://www.gov.mo/zh-hant/news/184940/. Accessed January 16, 2023.

Health Bureau organizes "Dementia Seminar" for private practitioners. NEWS GOV-MO. June 2, 2017. https://www.gcs.gov.mo/detail/zh-hant/N17FBJpkCy?. Accessed January 15, 2023.

Health Bureau holds dementia prevention seminar for civil servants and their family members. NEWS GOV-MO. July 25, 2017. https://www.gcs.gov.mo/detail/zh-hant/N17GYzseyS?. Accessed January 15, 2023.

Policy Address for Fiscal Year 2017. Macau Special Administrative Region Government Portal website. https://www.policyaddress.gov.mo/data/archive/zhant/2017_policy.pdf. Accessed January 20, 2023.

Establishment of Double Ninth Festival as Macao Special Administrative Region Senior Citizens' Day. Exmoo News. October 13, 2017. https://www.exmoo.com/article/42115.html. Accessed January 15, 2023.

Chronic Disease Prevention and Control Committee: Annual Report 2017 and Work Plan for 2018. Chronic Disease Prevention and Control Committee website. https://www.ssm.gov.mo/docs/26698/26698_0aa02f2e497041d4bdda52aff9d4d3e9_000.pdf. Accessed January 23, 2023.

Representatives of Health Bureau attended the 2nd Beijing International Neurodegenerative Disease Conference of the Chinese Society for Microcirculation Neurodegenerative Disease. NEWS GOV-MO. October 21, 2017. https://www.gcs.gov.mo/archive/showNews.php?PageLang=C&DataUcn=118112. Accessed January 15, 2023.

Government departments to enhance joint efforts to promote the well-being of the elderly. Social Welfare Bureau website. https://www.ias.gov.mo/ch/archives/gonggaotongzhi/%E2%80%8B%E9%95%B7%E8%80%85%E4%BA%8B%E5%8B%99%E5%A7%94%E5%93%A1%E6%9C%832017%E5%B9%B4%E7%AC%AC%E4%BA%8C%E6%AC%A1%E5%85%A8%E9%AB%94%E6%9C%83%E8%AD%B0?list=no&type=gonggaotongzhi. Accessed January 11, 2023.

Elderly Affairs Commission Second Plenary Meeting of 2017. Social Welfare Bureau website. https://www.ias.gov.mo/ch/archives/gonggaotongzhi/%E2%80%8B%E9%95%B7%E8%80%85%E4%BA%8B%E5%8B%99%E5%A7%94%E5%93%A1%E6%9C%832017%E5%B9%B4%E7%AC%AC%E4%BA%8C%E6%AC%A1%E5%85%A8%E9%AB%94%E6%9C%83%E8%AD%B0?list=no&type=gonggaotongzhi. Accessed January 11, 2023.

Chronic Disease Prevention and Control Committee Holds First Working Meeting of 2017. GOV.MO. March 28, 2017. https://www.gov.mo/zh-hant/news/181784/. Accessed January 11, 2023.

The Chronic Disease Prevention Committee held its second working meeting in 2017 to strengthen health education and screening for chronic diseases and promote the construction of "Healthy Macau, Happiness Home". GOV.MO. September 26, 2017. https://www.gov.mo/zh-hant/news/200650/. Accessed January 11, 2023.

Chronic Non-Communicable Diseases Newsletter No. 6. Chronic Disease Prevention and Control Committee website. https://www.ssm.gov.mo/docs/13540/13540_956a56f51368494fa8ed21dcbce826cd_000.pdf. Accessed January 11, 2023.

Interdepartmental Steering Group on Pension Protection Mechanism Holds First Plenary Meeting in 2017. GOV.MO. March 24, 2017. https://www.gov.mo/zh-hant/news/279548/. Accessed January 11, 2023.

Interdepartmental Steering Group on Pension Protection Mechanism Holds Second Plenary Meeting in 2017. GOV.MO. November 27, 2017. https://www.gov.mo/zh-hant/news/219757/. Accessed January 11, 2023.

Chief execution, Alzheimer’s Disease Association UK: Visit to Macao to exchange experiences. Exmoo News. May 15, 2017. https://www.exmoo.com/article/32934.html. Accessed January 15, 2023.

Localization of the Dementia Caregiver Support Program in Homes. Kiang Wu Nursing College of Macau website. http://www2.kwnc.edu.mo/?page_id=23485. Accessed January 13, 2023.

Benevolence Lights up my Later Life Ambassador's Event Focuses on Dementia. Kiang Wu Nursing College of Macau website. http://www2.kwnc.edu.mo/?p=15543. Accessed January 13, 2023.

Overseas Experience to Promote "Macau Friendly City": Kiang Wu Nursing College Visits Dementia Friendly Community in UK. Exmoo News. April 17, 2017. https://www.exmoo.com/article/31153.html. Accessed January 15, 2023.

Strengthening support for the elderly and their families: Kiang Wu Nursing College Trains Dementia Care Planners. Exmoo News. April 20, 2017. https://www.exmoo.com/article/31406.html. Accessed January 15, 2023.

Kiang Wu Nursing College and The Women's General Association of Macau Sign Cooperation Agreement: Aiming to Further Strengthen Their Network and Share Resources. Exmoo News. May 10, 2017. https://www.exmoo.com/article/32596.html. Accessed January 15, 2023.

Kiang Wu Nursing College and The Women's General Association of Macau Sign Cooperation Agreement: Aiming to Enhance Exchange and Training Resources Sharing. Exmoo News. May 11, 2017. https://www.exmoo.com/article/32665.html. Accessed January 15, 2023.

The Women's General Association of Macau teaches dementia prevention. Cyber CTM. November 1, 2017. https://www.cyberctm.com/zh_TW/news/detail/2033084#.ZAgFvT1BwuU. Accessed January 18, 2023.

The Macao Alzheimer’s Disease Association takes advantage of the policy to advance its work: The group calls on all sectors to join hands in preventing dementia. Exmoo News. January 23, 2017. https://www.exmoo.com/article/26125.html. Accessed January 14, 2023.

Caritas Macau Work Report 2017. Caritas Macau website. https://caritas.org.mo/en/uploads/ueditor/file/20190729/1564367786618672.pdf. Accessed January 13, 2023.

Seminar on Dementia Care and Safe and Rational Drug Use. The Pharmaceutical Society of Macao website. https://www.psmacao.org/products/%E5%A4%B1%E6%99%BA%E7%97%87%E7%85%A7%E8%AD%B7%E5%8F%8A%E5%AE%89%E5%85%A8%E5%90%88%E7%90%86%E7%94%A8%E8%97%A5%E7%A0%94%E8%A8%8E%E6%9C%83/. Accessed January 18, 2023.

S.K.H. Macau Social Services Coordination Office "Innovative Thinking" Seminar. Exmoo News. September 22, 2017. https://www.exmoo.com/article/41012.html. Accessed January 14, 2023.

Caregivers participated in a course on dementia and coronary heart disease care skills. Macao Carers Association Facebook website. https://www.facebook.com/permalink.php?story_fbid=pfbid02qmwcGtPxhd3dNoAXJ8gAHvyeztKPunXQnqWFZW5oSXXf72wYAyuzDJ6Zvcy8W5Pul&id=261787720842888. Accessed January 18, 2023.

Macao Magazine, Issue No. 116, Focus on Dementia, Prevention First, Proper Medical Treatment. NEWS GOV-MO. March 18, 2017. https://www.gcs.gov.mo/detail/zh-hant/N17CQ5oAyc?. Accessed January 11, 2023.

Policy Address for Fiscal Year 2018. Macau Special Administrative Region Government Portal website. https://www.gov.mo/zh-hant/wp-content/uploads/sites/4/2017/11/2018_policy_cn.pdf. Accessed January 20, 2023.

The Health Bureau and the Social Welfare Bureau launched the "Specialist Outreach Service Scheme" to further enhance the medical services for the elderly at risk in residential care homes. NEWS GOV-MO. March 9, 2018. https://www.gcs.gov.mo/detail/zh-hant/N18CIpW8tf?. Accessed January 11, 2023.

Forum on Dementia: Advocating Macau to introduce international successful experience. Exmoo News. October 27, 2018. https://www.exmoo.com/article/84171.html. Accessed January 14, 2023.

Outdoor emergency call-out service to be tried this month: a watch in hand for instant positioning. Exmoo News. July 24, 2018. https://www.exmoo.com/article/73585.html. Accessed January 15, 2023.

Ten-year action plan for the elderly in Macau (2016-2025): 2018 Annual Review Report. Macau Senior Citizen Service Information Website. http://www.ageing.ias.gov.mo/uploads/file/ffc5fd1c5160d71b3df3c6e19df15899.pdf. Accessed January 14, 2023.

"Walk for Health" 4th session on dementia prevention, about 750 people attended. NEWS GOV-MO. April 8, 2018. https://www.gcs.gov.mo/detail/zh-hant/N18DHYoYDE?. Accessed January 13, 2023.

Seminar on Elderly Services co-organized by the Health Bureau and the Hong Kong Institute of Medical Administration concluded successfully. Macao Dementia Friendly Community website. https://www.ssm.gov.mo/docs/13709/13709_24cf64dc52ac443bbfcb48ee76e63f6d_000.pdf. Accessed January 17, 2023.

Health Bureau held "Geriatric Medicine Lecture Series" and class commencement ceremony. NEWS GOV-MO. July 17, 2018. https://www.gcs.gov.mo/detail/zh-hant/N18GQWa7u1?. Accessed January 12, 2023.

Ilha Verde Health Centre starts operation today. Exmoo News. July 31, 2018. https://www.exmoo.com/article/73585.html. Accessed January 15, 2023.

Seminar on "Dementia Friendly Community" and "Macao SAR Dementia Friendly Community Charter" Project was successfully held. NEWS GOV-MO. August 2, 2018. https://www.gcs.gov.mo/detail/zh-hant/N18HBR8ydp?. Accessed January 12, 2023.

"Healthy Macau, Happiness Home - Building a Dementia Friendly Community" World Dementia Month Series organized by Health Bureau. NEWS GOV-MO. September 29, 2018. https://www.gcs.gov.mo/detail/zh-hant/N18Icsb5mE?. Accessed January 12, 2023.

Macau Health Bureau Delegation Visits UK, Promotes Multi-party Exchange and Cooperation to Create Barrier-Free Environment and Dementia-Friendly Community. GOV.MO. May 8, 2018. https://www.gov.mo/zh-hant/news/239879/. Accessed January 12, 2023.

2018 International Forum on "Healthy Macau, Happiness Home - Building a Dementia Friendly Community" is held today, calling for community awareness and promotion of dementia prevention and treatment. GOV.MO. October 27, 2018. https://www.gov.mo/zh-hant/news/261178/. Accessed January 12, 2023.

Alzheimer's Disease International Asia Pacific Regional Director visits the Dementia Medical Center. NEWS GOV-MO. October 26, 2018 https://www.gcs.gov.mo/detail/zh-hant/N18JZdLjHi?. Accessed January 13, 2023.

Joint meeting of health administration of the three places: sharing their experience and renewing cooperation agreements. Exmoo News. December 7, 2018. https://www.exmoo.com/article/89075.html. Accessed January 11, 2023.

Children's Integrated Assessment Center and Dementia Support Center will be operational in the middle of next month, the Health Bureau invited reporters to visit the Ilha Verde Health Centre today. NEWS GOV-MO. September 29, 2018. https://www.gcs.gov.mo/detail/zh-hant/N18KdC2wyY?. Accessed January 12, 2023.

Chronic Disease Prevention and Control Committee: Annual Report 2018 and Work Plan for 2019. Chronic Disease Prevention and Control Committee website. https://www.ssm.gov.mo/docs/26699/26699_1b48b13017114cf19925a094b6cac1b0_000.pdf. Accessed January 23, 2023.

Second Plenary Meeting of the Elderly Affairs Commission 2018. NEWS GOV-MO. September 19, 2018. https://www.gcs.gov.mo/detail/zh-hant/N18KdC2wyY?. Accessed January 11, 2023.

Chronic Disease Prevention and Control Committee holds first work session of 2018. GOV.MO. March 21, 2018. https://www.gov.mo/zh-hant/news/235328/. Accessed January 13, 2023.

Chronic Disease Prevention and Control Committee Holds Second Work Session of 2018. Macao Dementia Friendly Community website. https://www.ssm.gov.mo/docs/14782/14782_33d3b611028b42d8aba3c3989f8398ab_000.pdf. Accessed January 17, 2023.

Lifelong Learning Magazine Issue 38. Education and Youth Development Bureau website. https://www.dsedj.gov.mo/cep/lifelong_learning/index.htm. Accessed January 16, 2023.

Wong KC. Interrogatory: regarding the concern about the prevention and treatment of dementia and family support measures in Macao. Macao Legislative Council Written Question Website. https://www.al.gov.mo/uploads/attachment/2018-07/913985b5591642b780.pdf. Accessed January 14, 2023.

Wong IM. Replies to interrogatory raised by WONG Kit Cheng. https://www.al.gov.mo/uploads/attachment/2018-08/101465b83c12fcac2e.pdf. Accessed January 14, 2023.

Academy holds Memory Walk on World Alzheimer's Day to promote dementia awareness. Kiang Wu Nursing College of Macau website. http://www2.kwnc.edu.mo/?p=19122&doing_wp_cron=1678413795.6021420955657958984375. Accessed January 11, 2023.

Dementia Friendly Community. Kiang Wu Nursing College of Macau website. http://www2.kwnc.edu.mo/ueditor/php/upload/file/20190128/1548664076792910.pdf. Accessed January 11, 2023.

Survey on Public Awareness and Attitude towards Dementia in Macau. Kiang Wu Nursing College of Macau website. http://www2.kwnc.edu.mo/?page_id=23478. Accessed January 11, 2023.

Lam Nogueira BOC, Li L, Meng LR, et al. Clinical characteristics and quality of life of older adults with cognitive impairment in Macao. Psychogeriatrics. 2018;18(3):182-189. doi:10.1111/psyg.12306

Caritas Macau hosts seminar to share experience in caring for people with dementia. TDM. December 12, 2018. https://www.tdm.com.mo/zh-hant/news-detail/388839?date=2018.12.12. Accessed January 16, 2023.

Caritas Macau Work Report 2018. Caritas Macau website. https://www.caritas.org.mo/uploads/ueditor/file/20200107/1578372532472968.pdf. Accessed January 13, 2023.

The Chief Executive met with the Executive Director of Caritas Macau. GOV.MO. October 24, 2018. https://www.gov.mo/zh-hant/news/260790/. Accessed January 13, 2023.

Organizing a carnival for the elderly: Promoting the spirit of public care for the elderly. Exmoo News. November 25, 2018. https://www.exmoo.com/article/87563.html. Accessed January 11, 2023.

Caritas Macau Visits Taiwan Dementia Services. Jornal Cheng Pou. September 12, 2018. http://www.chengpou.com.mo/dailynews/141275.html. Accessed January 12, 2023.

Caritas Newsletter 135. Caritas Macau website. https://www.caritas.org.mo/uploads/ueditor/file/20190729/1564374082398059.pdf. Accessed January 13, 2023.

Macao Carers Association will hold an exhibition tomorrow: Caring for the Health of the Elderly. Exmoo News. March 1, 2018. https://www.exmoo.com/article/56488.html. Accessed January 12, 2023.

Lectures on intellectual disability and dementia were well received. Associação dos Familiares Encarregados dos Deficientes Mentais de Macau website. http://www.afedmm.org.mo/hl/news/detail.html?ids=23. Accessed January 14, 2023.

Chronic Non-Communicable Diseases Newsletter No. 7. Chronic Disease Prevention and Control Committee website. https://www.ssm.gov.mo/docs/14463/14463_266a79e297f14f79a7a6be4ad5d1cf1d_000.pdf. Accessed January 11, 2023.

The Women' s General Association of Macau Carnival to promote dementia prevention. Jornal Cheng Pou. October 16, 2018. http://www.chengpou.com.mo/dailynews/254.html. Accessed January 12, 2023.

Dementia Seminar. Pui Ching Middle School Student Association Facebook website. https://www.facebook.com/431121506925640/posts/1575548082482971/?paipv=0&eav=AfbB21CvfJZzP72XCwr2qD4cSqQlLZd5xLwalt7yfsyv-rITkzGjsQWJ3nNMfjufLU8&_rdr. Accessed January 17, 2023.

The First Cross-Straits, Hong Kong and Macao Nursing Development Forum introduces the Dementia Diagnosis and Treatment Centre. Macao Dementia Friendly Community website. https://www.ssm.gov.mo/apps1/mdfc/ch.aspx#clg12684. Accessed January 17, 2023.

Presentation on the "Macao SAR Dementia Friendly Community Charter" project was successfully held. NEWS GOV-MO. July 18, 2019. https://www.gcs.gov.mo/detail/zh-hant/N19GRMi3lX?. Accessed January 11, 2023.

Health Bureau and Social Welfare Bureau discussed with more than 100 organizations to build a dementia-friendly community. NEWS GOV-MO. January 30, 2019. https://www.gcs.gov.mo/detail/zh-hant/N19AdSOYzw?. Accessed January 10, 2023.

Dementia Support Center Education and Training Program to enhance community support for dementia patients and caregivers. NEWS GOV-MO. January 30, 2019. https://www.gcs.gov.mo/detail/zh-hant/N19DKggScr?. Accessed January 10, 2023.

Health Bureau and Social Welfare Bureau co-organized a training workshop to build a dementia-friendly community. Exmoo News. November 25, 2018. https://www.exmoo.com/article/107209.html. Accessed January 10, 2023.

Macau Social Service Facilities Guide. Social Welfare Bureau website. http://iasweb.ias.gov.mo/inst/detailChinese.jsp?id=C1022&call=1. Accessed January 17, 2023.

Ten-year action plan for the elderly in Macau (2016-2025): 2019 Annual Review Report. Macau Senior Citizen Service Information Website. http://www.ageing.ias.gov.mo/uploads/file/4351473638db089119dc6040a2455d90.pdf. Accessed January 14, 2023.

Ten-year action plan for the elderly in Macau (2016-2025): Mid-term evaluation sharing session. Macau Senior Citizen Service Information Website. http://www.ageing.ias.gov.mo/uploads/file/762f89ff052a98c7436cdb10d4b11adc.pdf. Accessed January 14, 2023.

Secretary for Social Affairs and Culture Visits Two Elderly Service Facilities. GOV.MO. January 31, 2019. https://www.gov.mo/zh-hant/news/271769/. Accessed January 13, 2023.

Guangzhou Geriatrics Summit. Macao Dementia Friendly Community website. https://www.ssm.gov.mo/apps1/mdfc/ch.aspx#clg12684. Accessed January 17, 2023.

"Healthy Macau, Happiness Home - Building a Dementia Friendly Community" World Dementia Month Series organized by Health Bureau. NEWS GOV-MO. September 24, 2019. https://www.gcs.gov.mo/detail/zh-hant/N19IXkIGtY?. Accessed January 10, 2023.

2019 International Forum on "Healthy Macau, Happiness Home - Building a Dementia Friendly Community" was held today to continue to promote and enhance the understanding of dementia in the community. NEWS GOV-MO. October 20, 2019. https://www.gcs.gov.mo/detail/zh-hant/N19JTjVemp?. Accessed January 10, 2023.

Chronic Disease Prevention and Control Committee: Annual Report 2019 and Work Plan for 2020. Chronic Disease Prevention and Control Committee website. https://www.ssm.gov.mo/docs/26700/26700_0c157196496b4e009a045f54101863da_000.pdf. Accessed January 23, 2023.

First Plenary Meeting of the Elderly Affairs Commission 2019. GOV.MO. March 6, 2019. https://www.gov.mo/zh-hant/news/274744/. Accessed January 13, 2023.

Second Plenary Meeting of the Elderly Affairs Commission 2019. GOV.MO. October 24, 2019. https://www.gov.mo/zh-hant/news/305503/. Accessed January 13, 2023.

Chronic Disease Prevention and Control Committee holds first work session of 2019. GOV.MO. March 8, 2019. https://www.gov.mo/zh-hant/news/275040/. Accessed January 13, 2023.

Chronic Non-Communicable Diseases Newsletter No. 9. Chronic Disease Prevention and Control Committee website. https://www.ssm.gov.mo/docs/16162/16162_92208f92b0694171b8329edd3414fc88_000.pdf. Accessed January 11, 2023.

Event Highlights. Macau Alzheimer’s Disease Association website. http://www.kwnc.edu.mo/mada/aph.html. Accessed January 10, 2023.

256 people participate in Rummikub contest to raise awareness about dementia. TDM. August 7, 2019. https://www.tdm.com.mo/zh-hant/news-detail/415204?date=2019.08.07. Accessed January 16, 2023.

Public Security Police Force and Kiang Wu Nursing College offer joint course on dementia. NEWS GOV-MO. March 18, 2019. https://www.gcs.gov.mo/detail/zh-hant/N19CRTOT6E?. Accessed January 10, 2023.

Dementia Friendly Community Photo Contest Successfully Completed. Kiang Wu Nursing College of Macau website. http://www2.kwnc.edu.mo/?p=21438. Accessed January 14, 2023.

Liang YH, Mao AM. The development of health care services for older adults in Macao after Macao's return to China.[in Chinese] Macau Journal of Nursing. 2019;31:53.

Macau University of Science and Technology Health Care Awareness Dementia Friendly Charter. Macao Daily News. October 2, 2019. https://www.ssm.gov.mo/docs/16829/16829_b7ca731bb2cb482d879012927b2c4b04_000.pdf. Accessed January 16, 2023.

"Small molecule photoacoustic Probe" developed by the University of Macau: More Effective assessment of dementia. Exmoo News. September 4, 2019. https://www.exmoo.com/article/122573.html. Accessed January 17, 2023.

Leung AYM, Leong CS, Cheong PL, et al. Investigating dementia literacy among community-dwelling adults and older adults in Macau: A mixed methods study. 2019. doi:10.21203/rs.2.18630/v1

Caritas Macau Work Report 2019. Caritas Macau website. https://www.caritas.org.mo/uploads/ueditor/file/20201019/1603073134489241.pdf Accessed January 13, 2023.

Caritas Newsletter 137. Caritas Macau website. https://www.caritas.org.mo/uploads/ueditor/file/20200107/1578370283695855.pdf. Accessed January 13, 2023.

Liaison Office of the Central People's Government in the Macao SAR visited Caritas Meng Fai Nursing Home to exchange views on the welfare of the elderly. Jornal “Va Kio”. August 22, 2019. http://www.vakiodaily.com/news/view/id/338790. Accessed January 17, 2023.

Caring for the Elderly, Caring for the Brain - Walking with You. Centro de Cuidados Especiais Longevidade Facebook website. https://www.facebook.com/324496448031902/posts/643901692758041/?paipv=0&eav=Afb-vHIYf_23AMjR3hN8775AAFhsbJda1gBXNqyhZa0ZbRtiqREgN-eGytJpuBMuvmk&_rdr. Accessed January 17, 2023.

Caritas Newsletter 142. Caritas Macau website. https://www.caritas.org.mo/uploads/ueditor/file/20200608/1591589002842485.pdf. Accessed January 13, 2023.

Caritas Macau Launches Dementia Teaching Kit. Jornal Cheng Pou. December 7, 2019. http://www.chengpou.com.mo/dailynews/183478.html. Accessed January 17, 2023.

Friends of Love call for brain health and prevention of dementia. Macao Daily News. September 19, 2019. https://www.ssm.gov.mo/docs/16827/16827_63c9a2df68784afebc61835458b1eedc_000.pdf. Accessed January 17, 2023.

Group concern for the growth of cross-border students. TDM. May 18, 2019. https://www.tdm.com.mo/zh-hant/news-detail/405801?date=2019.05.18. Accessed January 16, 2023.

The group held an annual conference to promote physical therapy to prevent dementia. TDM. May 19, 2019. https://www.tdm.com.mo/zh-hant/news-detail/405903?date=2019.05.19. Accessed January 16, 2023.

No downward trend in dementia age: people who were physically inactive were more likely to develop dementia. Exmoo News. May 19, 2019. https://www.exmoo.com/article/107725.html. Accessed January 17, 2023.

Macau therapists hope government will increase support for adult patients. Exmoo News. July 6, 2019. https://www.exmoo.com/article/114143.html. Accessed January 17, 2023.

Group volunteers regularly visit 80 households of elderly living alone. TDM. November 10, 2019. https://www.tdm.com.mo/zh-hant/news-detail/425377?date=2019.11.10. Accessed January 16, 2023.

Complexo de Serviços de Apoio ao Cidadão Senior “Pou Tai" Focuses on Dementia. Macao Daily News. December 6, 2019. https://www.ssm.gov.mo/docs/18122/18122_10e798d4684a42a2a9bb64a04ae06437_000.pdf. Accessed January 17, 2023.

TANO Smart Rehabilitation Game Training Competition. UGAMM Facebook website. https://www.facebook.com/macau.ugamm/posts/3032941143400586/. Accessed January 17, 2023.

UGAMM teaching elders to deal with pain and reduce suicidal thoughts: Strengthening support for the treatment of elderly with mild dementia. Exmoo News. February 6, 2019. https://www.exmoo.com/article/95261.html. Accessed January 17, 2023.

We invited the president of the Japan Recreational Therapy Association to come to the center to give direct instruction to our frontline colleagues on the use of "doll therapy". UGAMM Facebook website. https://www.facebook.com/macau.ugamm/posts/2553401601354545. Accessed January 17, 2023.

Macau Dementia Friendly Community Online Quiz Game with Prizes. NEWS GOV-MO. September 21, 2020. https://www.gcs.gov.mo/detail/zh-hant/N20IUSjbxV?. Accessed January 16, 2023.

Chief Executive: Provide on-the-job training in response to the epidemic and improve the exit mechanism for foreign employees. TDM. April 20, 2020. https://www.tdm.com.mo/zh-hant/news-detail/521525?date=2020.04.20. Accessed January 16, 2023.

Ten-year action plan for the elderly in Macau (2016-2025): 2020 Annual Review Report. Macau Senior Citizen Service Information Website. http://www.ageing.ias.gov.mo/uploads/file/a639cf464f5be6ae12a4a6466332dcea.pdf. Accessed January 14, 2023.

Elderly housing provides about 2,000 units: Praia do Manduco establishes elderly center with 100 additional places. Exmoo News. June 12, 2020. https://www.exmoo.com/article/150949.html. Accessed January 17, 2023.

Guidelines on prevention and management of lost elderly people with dementia: Family and Community. Social Welfare Bureau website. https://www.ssm.gov.mo/docs/26562/26562_fcccd5bc6c264c03b96348de9e7d8c5f_000.pdf. Accessed January 13, 2023.

Guidelines on prevention and management of lost elderly people with dementia: Social Service Units. Social Welfare Bureau website. https://www.ssm.gov.mo/docs/26677/26677_bac4a763e5f14d43b2571a25fff41853_000.pdf. Accessed January 13, 2023.

Setting up the first Dementia Integrated Services Centre and two more day centers for the elderly. Exmoo News. May 4, 2020. https://www.exmoo.com/article/147990.html. Accessed January 17, 2023.

The number of dementia patients in Macau is expected to reach 6,000 next year: Government and organizations to build dementia-friendly communities. Exmoo News. September 22, 2020. https://www.exmoo.com/article/158382.html. Accessed January 17, 2023.

Chronic Disease Prevention and Control Committee: Annual Report 2020 and Work Plan for 2021. Chronic Disease Prevention and Control Committee website. https://www.ssm.gov.mo/docs/26701/26701_c18e417f6501426299e122e9b6da4224_000.pdf. Accessed January 23, 2023.

Lo IL, Zeng W, Lei CI, Lam C, Lou HL. Knowledge, Attitude and Preventive Practice on Dementia Care among Primary Health Professionals in Macao. J Prev Alzheimers Dis. 2020;7(2):83-86. doi:10.14283/jpad.2020.10

Lo IL, Zeng W, Lei CI, Lam C, Lou HL. High School Students' Knowledge, Attitude and Preventive Practice of Dementia Care in Macao. Am J Alzheimers Dis Other Demen. 2020;35:1533317520948341. doi:10.1177/1533317520948341

Caritas Macau Work Report 2020. Caritas Macau website. https://www.caritas.org.mo/uploads/ueditor/file/20210927/1632738163928359.pdf. Accessed January 13, 2023.

Kiang Wu Nursing Home Trains 700 Police Officers in Dementia Coping Skills. Kiang Wu Nursing College of Macau website. http://www2.kwnc.edu.mo/?p=22997. Accessed January 18, 2023.

The School of Continuing Education of the Macau University of Science and Technology (MUST) actively promotes Macau-Mainland youth exchange. Macau University of Science and Technology website. https://www.must.edu.mo/news/31710-article01151011. Accessed January 18, 2023.

Wu JW, Che SL, Chuang YC, Van IK, Leong SM. Reliability and Validity of the Chinese Version of the Dementia Attitude Scale. [in Chinese] Nursing Journal Of Chinese People's Liberation Army. 2020;37(11)

Results published in international academic journal: Hong Kong and Macau medical team makes new breakthrough in dementia research. Exmoo News. June 23, 2020. https://www.exmoo.com/article/151714.html. Accessed January 17, 2023.

Leung AYM, Molassiotis A, Zhang J, et al. Dementia Literacy in the Greater Bay Area, China: Identifying the At-Risk Population and the Preferred Types of Mass Media for Receiving Dementia Information. Int J Environ Res Public Health. 2020;17(7):2511. Published 2020 Apr 7. doi:10.3390/ijerph17072511

Caritas Newsletter 145. Caritas Macau website. https://caritas.org.mo/uploads/ueditor/file/20210115/1610680387279963.pdf. Accessed January 13, 2023.

MGM and Caritas Macau co-host local tour for seniors with dementia. MGM China Holdings Limited website. https://cn.mgmchinaholdings.com/media-releases?item=377. Accessed January 18, 2023.

Taipa Senior Center Opens: Support Services for Residents Aged 55 and Older. Exmoo News. August 18, 2020. https://www.exmoo.com/article/155837.html. Accessed January 17, 2023.

Public Medical Center is deeply involved in the community: actively utilizing community medical functions. Exmoo News. November 9, 2020. https://www.exmoo.com/article/161514.html. Accessed January 17, 2023.

Dementia friendly community workshop. Jornal Cheng Pou. August 7, 2020. http://www.chengpou.com.mo/dailynews/191522.html. Accessed January 19, 2023.

Health Bureau and Social Welfare Bureau organized 4 online training sessions for Dementia Friendly Alliance. NEWS GOV-MO. June 29, 2021. https://www.gcs.gov.mo/detail/zh-hant/N21FcgXJdz?. Accessed January 16, 2023.

World Alzheimer’s Month encourages greater awareness, early prevention and detection of dementia. NEWS GOV-MO. September 22, 2021. https://www.gcs.gov.mo/detail/zh-hant/N21IV9XMfl?. Accessed January 16, 2023.

Chronic Disease Prevention and Control Committee: Annual Report 2021. Chronic Disease Prevention and Control Committee website. https://www.ssm.gov.mo/docs/26702/26702_3284fec4f17641c18a4902ce7d3ae6f6_000.pdf. Accessed January 23, 2023.

Lo IL, Zeng W, Lei CI, Lam C, Lou HL. Macao Dementia Policy: Challenges and prospects (innovative practice). Dementia (London). 2021;20(2):791-795. doi:10.1177/1471301219887612

1st Plenary Meeting of the Elderly Affairs Commission 2021. GOV.MO. April 28, 2021. https://www.gov.mo/zh-hant/news/375001/. Accessed January 17, 2023.

Chronic Disease Prevention and Control Committee Holds Work Session 2021. GOV.MO. July 27, 2021. https://www.gov.mo/zh-hant/news/643238/. Accessed January 17, 2023.

Si KL. To raise written questions on the support, publicity and education of dementia services in Macau. Macao Legislative Council Written Question Website. https://www.al.gov.mo/uploads/attachment/2021-05/6632860a775fb936b9.pdf. Published May 14, 2021. Accessed January 13, 2023.

Hon W. Replies to interrogatory raised by SI Ka-lon. Macao Legislative Council Written Question Website. https://www.al.gov.mo/uploads/attachment/2021-06/8693960c9b31532188.pdf. Accessed January 13, 2023.

MADA has joined schools to carry out dementia education. Macau Alzheimer’s Disease Association website. http://www.mada.org.mo/index.php?/detail/activity/55. Published April 8, 2021. Accessed January 17, 2023.

Dementia - "Early Diagnosis and Early Treatment". Caritas Macau website. https://www.caritas.org.mo/activities/608. Published May 31, 2021. Accessed January 17, 2023.

Caritas Newsletter 150. Caritas Macau website. https://www.caritas.org.mo/uploads/ueditor/file/20210730/1627619616130190.pdf. Accessed January 13, 2023.

KWNC announces three studies: Encouraging further education among the elderly can help prevent cognitive decline. Exmoo News. January 14, 2021. https://www.exmoo.com/article/166326.html. Accessed January 14, 2023.

Introduction to KiangWu Hospital April Medical seminars. Macao Daily News. March 24, 2021. http://www.macaodaily.com/html/2021-03/27/content_1504910.htm. Accessed January 17, 2023.

The college participates in the cross-regional collaborative research project, iSupport, by organizing a dementia caregiver sharing session and recruiting research participants. Kiang Wu Nursing College of Macau website. http://www2.kwnc.edu.mo/?p=25581. Accessed January 17, 2023.

Dementia Care Research and Practice Symposium to be Held. Kiang Wu Nursing College of Macau website. http://www2.kwnc.edu.mo/?p=24488. Accessed January 17, 2023.

Wu JW, Van IK, Vai MC. Analysis of the dementia population and their development trend in Macao. Macau Journal of Nursing. 2021;20(1):46-49. doi: 10.6729/MJN.202112/PP_20(1_2).004

Wu JW, Vai MC, Leong SM, et al. Reflections on a dementia screening program for the elderly in Macao community. Macau Journal of Nursing. 2021;20(1):37-41. doi: 10.6729/MJN.202108_20(1).004

Dementia Specialized Training for Macao Police Officers. Kiang Wu Nursing College of Macau website. http://www2.kwnc.edu.mo/?p=25332. Accessed January 17, 2023.

The University of Macau discovers a correlation between cognitive impairment in elderly individuals in Macao and Traditional Chinese Medicine constitution. GOV.MO. April 28, 2021. https://www.gov.mo/zh-hant/news/375042/. Accessed January 17, 2023.

2021 Caregiver Training Course Graduation Ceremony. Caritas Macau website. https://www.caritas.org.mo/activities/690. Published December 11, 2021. Accessed January 17, 2023.

Caritas Newsletter 153. Caritas Macau website. https://www.caritas.org.mo/uploads/ueditor/file/20220210/1644458591897411.pdf. Accessed January 13, 2023.

Caritas Macau Work Report 2021. Caritas Macau website. https://www.caritas.org.mo/caritas-annual-report/775. Accessed January 13, 2023.

Local Travel Tour. Macao Daily News. May 30, 2021. http://www.macaodaily.com/html/2021-05/30/content_1519547.htm. Accessed January 17, 2023.

Caritas celebrates its 70th anniversary with a seminar/conference. Macao Daily News. November 24, 2021. http://www.macaodaily.com/html/2021-11/24/content_1559088.htm. Accessed January 17, 2023.

MGM launches a new dementia care project. Jonal Cheng Pou. July 28, 2021. http://chengpou.com.mo/dailynews/203022.html. Accessed January 17, 2023.

UGAMM holds a seminar on the mental health of the elderly during the pandemic. UGAMM website. https://www.ugamm.org.mo/portal/ctr/cms/info/goToInfoView?info_id=4ccff633762847edb751366021010cf5. Accessed January 18, 2023.

Organization hosts events for dementia prevention. TDM. November 5, 2021. https://www.tdm.com.mo/zh-hant/news-detail/626078?date=2021.11.05. Accessed January 17, 2023.

Sands volunteers celebrate Mid-Autumn Festival with elderly individuals with dementia. Jonal Cheng Pou. September 22, 2021. http://www.chengpou.com.mo/dailynews/204796.html. Accessed January 17, 2023.

Macau's First Evidence-Based Study on Horticultural Therapy: Positive Therapeutic Effects for Elderly Individuals with Dementia. Exmoo.news. November 29, 2021. https://www.exmoo.com/article/188509.html. Accessed January 17, 2023.

Protecting Individuals with Dementia. Macao Community Volunteer Service Association Facebook website. https://www.facebook.com/Macau.MCVSA/videos/%E5%AE%88%E6%99%BA%E8%80%85/1240889319731684/?locale=ms_MY. Accessed January 17, 2023.

Women's Federation Wellness Center Promotes Dementia Prevention. The Women' s General Association of Macau website. https://www.macauwomen.org.mo/%E5%A9%A6%E8%81%AF%E9%A0%A4%E5%BA%B7%E4%B8%AD%E5%BF%83%E6%8E%A8%E5%BB%A3%E9%A0%90%E9%98%B2%E5%A4%B1%E6%99%BA%E7%97%87/. Accessed January 19, 2023.

Director of the Department of Social and Cultural Affairs pays visits to multiple social service facilities during the Lunar New Year. GOV.MO. February 4, 2022. https://www.gov.mo/zh-hant/news/867937/. Accessed January 17, 2023.

The first comprehensive dementia service center has commenced operations. TDM. February 12, 2022. https://www.tdm.com.mo/zh-hant/news-detail/657352?date=2022.02.12. Accessed January 17, 2023.

The Health Bureau actively responds to World Alzheimer's Month, committed to providing appropriate medical services and support to individuals with dementia. NEWS GOV-MO. September 20, 2022. https://www.gcs.gov.mo/detail/zh-hant/N22ITrlVxF?. Accessed January 11, 2023.

Services. Macao Social Welfare Bureau website. https://www.ias.gov.mo/ch/swb-services/individual-and-family-service/shrhjh. Accessed January 11, 2023.

No conditional increase in elderly pensions next year. Jornal of Cheng Pou. November 29, 2022. http://www.chengpou.com.mo/dailynews/218240.html. Accessed January 11, 2023.

Public Hospital will be hosting specialized health education seminars in June 2022. Macao Health Bureau website. https://www.ssm.gov.mo/docs/23257/23257_b5df1cfdf45d4ba5996b0807c0cdd8e1_000_.pdf. Accessed January 11, 2023.

Wong SM, Zeng W, Lo IL. Cohort Profile: The Dementia Registry in Macao. Am J Alzheimers Dis Other Demen. 2022;37:15333175211067124. doi:10.1177/15333175211067124

Second plenary meeting of the Elderly Affairs Committee in 2022. GOV.MO. September 20, 2022. https://www.gov.mo/zh-hant/news/931445/. Accessed January 11, 2023.

2022 Annual Work Meeting of the Chronic Disease Prevention Committee. NEWS GOV-MO. November 9, 2022. https://www.gcs.gov.mo/detail/zh-hant/N22KIwUVGE?. Accessed January 11, 2023.

Dementia Work Group Holds Dementia Prevention Seminar. Macao Dementia Friendly Community website. https://www.ssm.gov.mo/apps1/mdfc/ch.aspx#clg12684. Accessed January 17, 2023.

Programme in Dementia Care Organiser. Kiang Wu Nursing College of Macau website. http://www2.kwnc.edu.mo/?page_id=26477. Accessed January 17, 2023.

Che SL, Wu J, Chuang YC, Van IK, Leong SM. The Willingness to Help People With Dementia Symptoms Among Four Occupation Practitioners in Macao. Am J Alzheimers Dis Other Demen. 2022;37:15333175221139172. doi:10.1177/15333175221139172

Wu J, Leong SM, Che SL, Van IK, Chuang YC. Comparisons of Dementia Knowledge and Attitudes among the Youth and Older Adults: Insights from the Construal Level Theory Perspective. Int J Environ Res Public Health. 2022;19(4):1928. Published 2022 Feb 9. doi:10.3390/ijerph19041928

Chuang CY. To formulate Traditional Chinese Medicine constitution differentiation nursing for older people with cognitive impairment in Macau based on constitutional identification theory. Macau Journal of Nursing. 2022;21(1):22-26. doi:10.6729/MJN.202210_21(1).004

Macao Polytechnic Institute's Elderly College organizes a "Understanding Dementia" health lecture for seniors. NEWS GOV-MO. May 25, 2022. https://www.gcs.gov.mo/detail/zh-hant/N22EYFz5HI?. Accessed January 11, 2023.

The Alzheimer's Disease International (ADI) conducts a large-scale survey and research on dementia in 2022. Macau Alzheimer’s Disease Association website. http://www.mada.org.mo/index.php?/detail/activity/36. Accessed January 11, 2023.

"Move Together, Prevent Dementia" Brain-Hand Exercise. “Peng-On Tung" Emergency Assistance Service Center Facebook. https://www.facebook.com/watch/?v=588604362248991. Accessed January 11, 2023.

Social Welfare Bureau proposes the establishment of additional elderly centers. TDM. January 28, 2022. https://www.tdm.com.mo/zh-hant/news-detail/652946?date=2022.01.28. Accessed January 17, 2023.

The Macau Elderly Care Association advocates for supporting caregivers to alleviate their stress. Macao Daily News. March 14, 2022. http://www.macaodaily.com/html/2022-03/14/content_1583518.htm. Accessed January 17, 2023.

CEM organizes a competition with the aim of raising awareness for the elderly and dementia. TDM. October 15, 2022. https://www.tdm.com.mo/zh-hant/news-detail/755332?date=2022.10.15. Accessed January 17, 2023.

Zeng W, Tang J Y,Lei M, et al. Chinese expert consensus on the development of dementia-friendly communities in Guangdong-Hong Kong-Macao Greater Bay Area. Chin J Nervous Mental Dis. 2022;48(4):193-205.

St. Joseph's University collaborates with the Federation of Trade Unions to promote the development of social services. TDM. November 22, 2022. https://www.tdm.com.mo/zh-hant/news-detail/772204?date=2022.11.22. Accessed January 17, 2023.

The Federation of Trade Unions organizes a series of wellness activities to prevent dementia. Macao Daily News. May 6, 2022. http://www.macaodaily.com/html/2022-05/06/content_1595031.htm. Accessed January 17, 2023.

Wong SM, Zeng W, Lo IL, Lam C, Lou HL. Development and Psychometric Evaluation of a Chinese Instrument of Knowledge, Attitude and Preventive Practice on Dementia Care in Macao. Am J Alzheimers Dis Other Demen. 2023;38:15333175221149358. doi:10.1177/15333175221149358

MGM organizes a series of Lunar New Year activities to bring warmth to the elderly. Jornal of Cheng Pou. January 20, 2023. http://www.chengpou.com.mo/dailynews/219797.html. Accessed January 25, 2023.

Enhancing dementia screening to build a dementia-friendly community. Jornal of Cheng Pou. January 4, 2023. http://www.chengpou.com.mo/dailynews/219272.html. Accessed January 17, 2023.

Research suggests that inadequate water consumption is associated with an increased risk of chronic diseases. Not only does it accelerate the aging process, but it may also contribute to a shorter lifespan. Exmoo.news. January 4, 2023. https://www.exmoo.com/article/212701.html. Accessed January 17, 2023.

1. Download section. Macao Dementia Friendly Community website. https://www.ssm.gov.mo/apps1/mdfc/ch.aspx#clg12684. Accessed January 17, 2023.

**3. Scientific publications conducted about dementia by research agencies in Macao**

|  | **Medical/social service/needs** | **Treatment/diagnosis/screening/detection** |
| --- | --- | --- |
| **UM research** | | |
| Chen C, Wang L, Cao F, et al. Formulation of 20 (S)-protopanaxadiol nanocrystals to improve oral bioavailability and brain delivery. International Journal of Pharmaceutics. 2016;497(1-2):239-247. |  | treatment - potential new drug for neurodegenerative disease |
| Zhang QE, Sha S, Ungvari GS, et al. Demographic and clinical profile of patients with dementia receiving electroconvulsive therapy: a case-control study. The Journal of ECT. 2016;32(3):183-186. |  | treatment - electroconvulsive therapy |
| Wang SF, Wu MY, Cai CZ, et al. Autophagy modulators from traditional Chinese medicine: Mechanisms and therapeutic potentials for cancer and neurodegenerative diseases. Journal of ethnopharmacology. 2016;194:861-876. |  | treatment - TCM drug discovery |
| Ye RD. Deficiency of macrophage migration inhibitory factor attenuates tau hyperphosphorylation in mouse models of Alzheimer's disease. The FASEB Journal. 2016;30:707. |  | treatment&prevention - new targets |
| Zhou X, Cui G, Tseng HH, et al. Vascular contributions to cognitive impairment and treatments with traditional Chinese medicine. Evidence-Based Complementary and Alternative Medicine. 2016;23. |  | treatment - Traditional Chinese Medicine |
| Tan Y, Ren H, Shi Z, et al. Endogenous docosahexaenoic acid (dha) prevents aβ1–42 oligomer-induced neuronal injury. Molecular Neurobiology. 2016;53(5):3146-3153. |  | treatment - neuroprotective in pathogenesis processes |
| Chen D, Liu F, Wan JB, et al. Effect of major royal jelly proteins on spatial memory in aged rats: Metabolomics analysis in urine. Journal of Agricultural and food Chemistry. 2017;65(15):3151-3159. |  | treatment - major royal-jelly proteins may be used for prevention of cognitive impairement |
| Yang C, Cai CZ, Song JX, et al. NRBF2 is involved in the autophagic degradation process of APP-CTFs in Alzheimer disease models. Autophagy. 2017;13(12):2028-2040. |  | treatment - NRBF2 plays role in regulating degradation of APP-CTFs though modulating autophagy |
| Chong CM, Ai N, Ming-Yuen Lee S. ROCK in CNS: different roles of isoforms and therapeutic target for neurodegenerative disorders. Current drug targets. 2017;18(4):455-462. |  | treatment - development of a promising therapeutic target (ROCK) inhibitors |
| Wu X, Kosaraju J, Zhou W, et al. Neuroprotective effect of SLM, a novel carbazole-based fluorophore, on SH-SY5Y cell model and 3xTg-AD mouse model of Alzheimer’s disease. Acs Chemical Neuroscience. 2017;8(3):676-685. |  | treatment - a carbazole-based fluorophore (SLM) showed neuroprotective effect |
| Wu X, Kosaraju J, Tam KY. SLM, a novel carbazole-based fluorophore attenuates okadaic acid-induced tau hyperphosphorylation via down-regulating GSK-3β activity in SH-SY5Y cells. European Journal of Pharmaceutical Sciences. 2017;110:101-108. |  | treatment - SLM as potential compound for AD |
| Kosaraju J, Holsinger RM, Guo L, et al. Linagliptin, a dipeptidyl peptidase-4 inhibitor, mitigates cognitive deficits and pathology in the 3xTg-AD mouse model of Alzheimer’s disease. Molecular neurobiology. 2017;54(8):6074-6084. |  | treatment - linagliptin possessed neuroprotective properties |
| Zeng Z, Xu J, Zheng W. Artemisinin protects PC12 cells against β-amyloid-induced apoptosis through activation of the ERK1/2 signaling pathway. Redox biology. 2017;12:625-633. |  | prevention & treatment - potential application of artemisinin |
| Wu AG, Zeng W, Wong VK, et al. Hederagenin and α-hederin promote degradation of proteins in neurodegenerative diseases and improve motor deficits in MPTP-mice. Pharmacological research. 2017;115:25-44. |  | treatment - potential therapeutic effect of Hedera helix |
| Wang YY, Zheng W, Ng CH, et al. Meta‐analysis of randomized, double‐blind, placebo‐controlled trials of melatonin in Alzheimer's disease. International Journal of Geriatric Psychiatry. 2017;32(1):50-57. |  | treatment - sleep disturbance |
| Zhou W, Hu X, Tam KY. Systemic clearance and brain distribution of carbazole-based cyanine compounds as Alzheimer’s disease drug candidates. Scientific reports. 2017;7(1):1-10. |  | treatment - drug development |
| Zeng Z, Wang D, Gaur U, et al. H2O2 attenuates IGF-1R tyrosine phosphorylation and its survival signaling properties in neuronal cells via NR2B containing NMDA receptor. Oncotarget. 2017;8(39):65313. |  | treatment - alternative therapeutic agent for oxidative stress related neurodegenerative disease |
| Luo C, Ren H, Yao X, et al. Enriched brain omega-3 polyunsaturated fatty acids confer neuroprotection against microinfarction. EBioMedicine. 2018;32:50-61. |  | treatment or prophylaxis - ω-3 PUFAs |
| Meng FC, Wu ZF, Yin ZQ, et al. Coptidis rhizoma and its main bioactive components: recent advances in chemical investigation, quality evaluation and pharmacological activity. Chinese Medicine. 2018;13(1):1-8. |  | treatment - anti-Alzheimer (Traditional Chinese Medicine) |
| Chong CM, Ke M, Tan Y, et al. Presenilin 1 deficiency suppresses autophagy in human neural stem cells through reducing γ-secretase-independent ERK/CREB signaling. Cell death & disease. 2018;9(9):1-3. |  | treatment - autophagy suppression |
| Hung CH, Cheng SS, Cheung YT, et al. A reciprocal relationship between reactive oxygen species and mitochondrial dynamics in neurodegeneration. Redox biology. 2018;14:7-19. |  | treatment & pathogenesis - potential therapeutic target |
| Liu K, Lin HH, Pi R, et al. Research and development of anti-Alzheimer’s disease drugs: an update from the perspective of technology flows. Expert Opinion on Therapeutic Patents. 2018;28(4):341-350. |  | treatment - improve theory; use of traditional herbal medicines |
| Meng FC, Wu ZF, Yin ZQ, et al. Coptidis rhizoma and its main bioactive components: recent advances in chemical investigation, quality evaluation and pharmacological activity. Chinese Medicine. 2018;13(1):1-8. |  | treatment - anti-alzheimer (coptidis rhizoma) |
| Zhou X, Xiao W, Su Z, et al. Hippocampal proteomic alteration in triple transgenic mouse model of Alzheimer’s disease and implication of PINK 1 regulation in donepezil treatment. Journal of proteome research. 2018;18(4):1542-1552. |  | treatment - potential therapeutic target (PINK1) |
| Wang C, Saar V, Leung KL, et al. Human amyloid β peptide and tau co-expression impairs behavior and causes specific gene expression changes in Caenorhabditis elegans. Neurobiology of Disease. 2018;109:88-101. |  | fundamental neurobiologic processes |
| Wu X, Kosaraju J, Zhou W, et al. SLOH, a carbazole-based fluorophore, mitigates neuropathology and behavioral impairment in the triple-transgenic mouse model of Alzheimer's disease. Neuropharmacology. 2018;131:351-363. |  | treatment - potential multi-target molecule |
| Zhang QE, Ling S, Li P, et al. The association between urinary Alzheimer-associated neuronal thread protein and cognitive impairment in late-life depression: a controlled pilot study. International Journal of Biological Sciences. 2018;14(11):1497. |  | diagnosis - biomarker |
| Zhao ZW, Yuan Z, Zhao DQ, et al. The effect of V‐ATPase function defects in pathogenesis of Alzheimer's disease. CNS Neuroscience & Therapeutics. 2018;24(9):837. |  | pathogenesis; treatment |
| Wu X, Kosaraju J, Tam KY. Anti-neuroinﬂammatory eﬀects of SLOH in Aβ-induced BV-2 microglial cells and 3xTg-AD mice involve the inhibition of GSK-3β. Neuroscience Letters. 2018;687:207-215. |  | potential treatment - SLOH |
| Zhao ZW, Yuan Z, Zhao DQ, et al. The effect of V‐ATPase function defects in pathogenesis of Alzheimer's disease. CNS Neuroscience & Therapeutics. 2018;24(9):837. |  | pathogenesis & treatment |
| Wu MY, Song JX, Wang SF, et al. Selective autophagy: The new player in the fight against neurodegenerative diseases?. Brain Research Bulletin. 2018;137:79-90. |  | treatment - therapeutic effects against neurodegenerative diseases |
| Leung CH, Zhang JT, Yang GJ, et al. Emerging Screening Approaches in the development of Nrf2–Keap1 protein–protein interaction inhibitors. International Journal of Molecular Sciences. 2019;20(18):4445. |  | treatment - potential drug target |
| Zhao X, Zeng Z, Gaur U, et al. Metformin protects PC12 cells and hippocampal neurons from H2O 2‐induced oxidative damage through activation of AMPK pathway. Journal of cellular physiology. 2019:234(9):16619-16629. |  | potential prevention & treatment - metformin |
| Liao Q, Li S, Siu SW, et al. Novel neurotoxic peptides from Protopalythoa variabilis virtually interact with voltage-gated sodium channel and display anti-epilepsy and neuroprotective activities in zebrafish. Archives of Toxicology. 2019;93(1):189-206. |  | treatment - pharmacology, potential manage neurodegenerative disorder |
| Wu AG, Teng JF, Wong VK, et al. Novel steroidal saponin isolated from Trillium tschonoskii maxim. exhibits anti-oxidative effect via autophagy induction in cellular and Caenorhabditis elegans models. Phytomedicine. 2019;65:153088. |  | tratment - anti-oxidative effects of the components from TTM |
| Wang S, Sheng Z, Yang Z, et al. Activatable Small‐Molecule Photoacoustic Probes that Cross the Blood–Brain Barrier for Visualization of Copper (II) in Mice with Alzheimer's Disease. Angewandte Chemie International Edition. 2019;58(36):12415-12419. |  | pathogenesis; imaging |
| Cai CZ, Zhou HF, Yuan NN, et al. Natural alkaloid harmine promotes degradation of alpha-synuclein via PKA-mediated ubiquitin-proteasome system activation. Phytomedicine. 2019;61:152842. |  | treatment - potential therapeutic effects on against neurodegenerative diseases |
| Ozsahin I, Sekeroglu B, Mok GS. The use of back propagation neural networks and 18F-Florbetapir PET for early detection of Alzheimer’s disease using Alzheimer’s Disease Neuroimaging Initiative database. Plos one. 2019;14(12):e0226577. |  | detection - neuroimaging |
| Zhao X, Fang J, Li S, et al. Artemisinin attenuated hydrogen peroxide (H2O2)-induced oxidative injury in SH-SY5Y and hippocampal neurons via the activation of AMPK pathway. International journal of molecular sciences. 2019;20(11):2680. |  | treatment - potential therapeutic agent for neurodegenerative diseases |
| Li S, Zhao X, Lazarovici P, Zheng W. Artemether Activation of AMPK/GSK3β(ser9)/Nrf2 Signaling Confers Neuroprotection towards β-Amyloid-Induced Neurotoxicity in 3xTg Alzheimer’s Mouse Model. Oxidative Medicine and Cellular Longevity. 2019;2019:1–24. |  | treatment - drug development |
| Ozsahin I, Sekeroglu B, Mok GS. The use of back propagation neural networks and 18F-Florbetapir PET for early detection of Alzheimer’s disease using Alzheimer’s Disease Neuroimaging Initiative database. Plos one. 2019;14(12):e0226577. |  | early detection |
| Chong CM, Su H, Lu JJ, et al. The effects of bioactive components from the rhizome of Salvia miltiorrhiza (Danshen) on the characteristics of Alzheimer’s disease. Chinese Medicine. 2019;14(1):1-10. |  | treatment - Danshen component |
| Yuan NN, Cai CZ, Wu MY, et al. Neuroprotective effects of berberine in animal models of Alzheimer’s disease: a systematic review of pre-clinical studies. BMC complementary and alternative medicine. 2019;19(1):1-10. |  | treatment - berberine can be a promising multipotent agent to combat Alzheimer's disease |
| Chen JY, Zhu Q, Zhang S, et al. Resveratrol in experimental Alzheimer’s disease models: A systematic review of preclinical studies. Pharmacological Research. 2019;150:104476. |  | treatment - Resveratrol demonstrates neuroprotective effects |
| Yang GJ, Liu H, Ma DL, et al. Rebalancing metal dyshomeostasis for Alzheimer’s disease therapy. JBIC Journal of Biological Inorganic Chemistry. 2019;24(8):1159-1170. |  | treatment - the use of metal homeostasis rebalancing agents |
| Zhou X, Wang L, Xiao W, et al. Memantine improves cognitive function and alters hippocampal and cortical proteome in triple transgenic mouse model of Alzheimer's disease. Experimental neurobiology. 2019;28(3):390. |  | treatment - novel view on the complexity of protein responses induced by memantine |
| Bian Y, Zhao C, Lee SM. Neuroprotective potency of saffron against neuropsychiatric diseases, neurodegenerative diseases, and other brain disorders: from bench to bedside. Frontiers in Pharmacology. 2020;1602. |  | treatment - developing therapeutic drug from saffron |
| Zhang XW, Chen JY, Ouyang D, et al. Quercetin in animal models of Alzheimer’s disease: A systematic review of preclinical studies. International Journal of Molecular Sciences. 2020;21(2):493. |  | treatment - pharmacological mechnisms of quercetin; developing therapeutic drug from quercetin |
| Liu Q, Yan L, Huang M, et al. Experimental alcoholism primes structural and functional impairment of the glymphatic pathway. Brain, Behavior, and Immunity. 2020;85:106-119. |  | pathology - impaired glymphatic functions and reduced parenchymal Aβ clearance may contribute to the development of cognitive decline and dementia in alcoholism |
| Li CW, Deng MZ, Gao ZJ, et al. Effects of compound K, a metabolite of ginsenosides, on memory and cognitive dysfunction in db/db mice involve the inhibition of ER stress and the NLRP3 inflammasome pathway. Food & function. 2020;11(5):4416-4427. |  | treatment - ginsenoside compound K improve memory and cognitive dysfunction |
| Zhang S, Zhu Q, Chen JY, et al. The pharmacological activity of epigallocatechin-3-gallate (EGCG) on Alzheimer's disease animal model: A systematic review. Phytomedicine. 2020;79:153316. |  | treatment - effectiveness of Epigallocatechin-3-O-gallate (EGCG) |
| Xie Y, Yan L, Zeng H, et al. Fish oil protects the blood–brain barrier integrity in a mouse model of Alzheimer’s disease. Chinese Medicine. 2020;15(1):1-11. |  | pathophysiological mechanism; early protection - fish oil supplement |
| Yuen SC, Zhu H, Leung SW. A systematic bioinformatics workflow with meta-analytics identified potential pathogenic factors of Alzheimer’s disease. Frontiers in neuroscience. 2020;14:209. |  | pathogenesis - reduced Aβ clearance was associated with genes encodning Fyn and EGFR |
| Yan L, Xie Y, Satyanarayanan SK, et al. Omega-3 polyunsaturated fatty acids promote brain-to-blood clearance of β-Amyloid in a mouse model with Alzheimer’s disease. Brain, Behavior, and Immunity. 2020;85:35-45. |  | pathology - impaired BBB transporty functioncan contribute to Aβ accumulation; progression prevention - omega-3 PUFAs |
| Ju Y, Tam KY. 9R, the cholinesterase and amyloid beta aggregation dual inhibitor, as a multifunctional agent to improve cognitive deficit and neuropathology in the triple-transgenic Alzheimer's disease mouse model. Neuropharmacology. 2020;181:108354. |  | treatment - potential multi-target compound |
| Zhao X, Li S, Gaur U, et al. Artemisinin improved neuronal functions in Alzheimer's disease animal model 3xtg mice and neuronal cells via stimulating the ERK/CREB signaling pathway. Aging and disease. 2020;11(4):801. |  | treatment & prevention - artemisinin as a new multi-target drug |
| Chen Y, Li G, Law HC, et al. Determination of Oxyphylla A Enantiomers in the Fruits of Alpinia oxyphylla by a Chiral High-Performance Liquid Chromatography–Multiple Reaction Monitoring–Mass Spectrometry Method and Comparison of Their In Vivo Biological Activities. Journal of Agricultural and Food Chemistry. 2020;68(40):11170-11181. |  | treatment - potential therapeutic for neurodegenerative diseases () |
| Ju Y, Chakravarty H, Tam KY. An Isoquinolinium Dual Inhibitor of Cholinesterases and Amyloid β Aggregation Mitigates Neuropathological Changes in a Triple-Transgenic Mouse Model of Alzheimer’s Disease. ACS Chemical Neuroscience. 2020 ;11(20):3346-3357. |  | treatment - dual inhibition of cholinesterases and Aβ aggregation |
| Chakravarty H, Ju Y, Chen WH, et al. Dual targeting of cholinesterase and amyloid beta with pyridinium/isoquinolium derivatives. Drug Development Research. 2020;81(2):242-255. |  | treatment - dual-target molecules development |
| Peng Y, Tao H, Wang S, et al. Dietary intervention with edible medicinal plants and derived products for prevention of Alzheimer's disease: A compendium of time-tested strategy. Journal of Functional Foods. 2021;81:104463. |  | pathogenic mechanisms; prevention & treatment - plant-based dietary materials |
| Luo JF, Dong Y, Chen JY, et al. The effect and underlying mechanisms of garlic extract against cognitive impairment and Alzheimer's disease: A systematic review and meta-analysis of experimental animal studies. Journal of Ethnopharmacology. 2021;280:114423. |  | treatment - garlic extract may alleviate cognitive impairment and neuropathology in AD animal models |
| Deng Z, Dong Y, Zhou X, et al. Pharmacological modulation of autophagy for Alzheimer’s disease therapy: Opportunities and obstacles. Acta Pharmaceutica Sinica B. 2021. |  | treatment - novel autophagy activators |
| Bian Y, Chen Y, Wang X, et al. Oxyphylla A ameliorates cognitive deficits and alleviates neuropathology via the Akt-GSK3β and Nrf2-Keap1-HO-1 pathways in vitro and in vivo murine models of Alzheimer's disease. Journal of advanced research. 2021 Dec 1;34:1-2. |  | treatment - natural products & their constituents |
| Wang EJ, Wu MY, Lu JH. Ferulic acid in animal models of Alzheimer’s disease: A systematic review of preclinical studies. Cells. 2021;10(10):2653. |  | treatment - ferulic acid reduce Aβ deposition and improving memory in preclinical experiments |
| Zeng LN, Zong QQ, Xu SW, et al. Oral health in patients with dementia: A meta‐analysis of comparative and observational studies. International Journal of Geriatric Psychiatry. 2021;36(4):467-478. |  | screening & treatment - oral health |
| Huang X, Wang C, Chen L, et al. Human amyloid beta and α-synuclein co-expression in neurons impair behavior and recapitulate features for Lewy body dementia in Caenorhabditis elegans. Biochimica et Biophysica Acta (BBA)-Molecular Basis of Disease. 2021;1867(10):166203. |  | pathogenesis mechanism |
| Ju Y, Tam KY. Pathological mechanisms and therapeutic strategies for Alzheimer’s disease. Neural Regeneration Research. 2022;17(3):543. |  | treatment - multi-target therapies |
| Xie L, Zhu Q, Lu J. Can We Use Ginkgo biloba Extract to Treat Alzheimer’s Disease? Lessons from Preclinical and Clinical Studies. Cells. 2022;11(3):479. |  | treatment - ginkgo biloba extract can use as anti-alzheimer effects in animal experiments |
| Shen S, Liao Q, Wong YK, et al. The role of melatonin in the treatment of type 2 diabetes mellitus and Alzheimer's disease. International Journal of Biological Sciences. 2022;18(3):983. |  | therapeutic potentials of melatonin in preventing the acceleration of AD in T2DM |
| Chong CM, Tan Y, Tong J, et al. Presenilin-1 F105C mutation leads to tau accumulation in human neurons via the Akt/mTORC1 signaling pathway. Cell & bioscience. 2022;12(1):1-5. |  | treatment - pharmaceutical blocking of mammalian target of rapamycin (mTOR) |
| Liu W, He K, Wu D, et al. Natural Dietary Compound Xanthohumol Regulates the Gut Microbiota and Its Metabolic Profile in a Mouse Model of Alzheimer’s Disease. Molecules. 2022;27(4):1281. |  | treatment - xanthohumol may have a neuroprotective role; novel prophylactic and therapeutic approach |
| Ai R, Zhuang XX, Anisimov A, et al. A synergized machine learning plus cross-species wet-lab validation approach identifies neuronal mitophagy inducers inhibiting Alzheimer disease. Autophagy. 2022;18(4):939-941. |  | treatment - compound screening; speed up the development of novel treatments for AD via AI and preclinical drug research |
| Zhao Y, Tan DC, Peng B, et al. Neuroendocrine–Immune Regulatory Network of Eucommia ulmoides Oliver. Molecules. 2022;27(12):3697. |  | treatment - potential herbal products use for neurodegenerative disease |
| Yuen SC, Lee SM, Leung SW. putative factors interfering cell cycle re-entry in Alzheimer’s disease: an omics study with differential expression meta-analytics and co-expression profiling. Journal of Alzheimer's Disease. 2022;(Preprint):1-26. |  | pathologenesis mechanism |
| Wang YT, Lu JH. Chaperone-Mediated Autophagy in Neurodegenerative Diseases: Molecular Mechanisms and Pharmacological Opportunities. Cells. 2022;11(14):2250. |  | treatment - therapeutic potential of Chaperone-mediated autophagy (CMA) modulation for neurodegenerative disease |
| **Database research** | | |
| Wong SM, Zeng W, Lo IL. Cohort Profile: The Dementia Registry in Macao. Am J Alzheimers Dis Other Demen. 2022;37:15333175211067124. | dementia registry aims to better understanding of health service use, medicines, hospitalizations, mortality |  |
| Lam Nogueira BO, Li L, Meng LR, et al. Clinical characteristics and quality of life of older adults with cognitive impairment in Macao. Psychogeriatrics. 2018 ;18(3):182-189. | social needs - further appropriate strategies and need to improve access to treatment |  |
| Hu SXX, Lei WI, Chao KK, et al. Common chronic health problems and life satisfaction among Macau elderly people. International Journal of Nursing Sciences. 2016;3(4):367-370. | exploring the most affect life satisfaction of elderly people living in community |  |
| Wu J, Leong S-M, Che S-L, et al. Comparisons of Dementia Knowledge and Attitudes among the Youth and Older Adults: Insights from the Construal Level Theory Perspective. International journal of environmental research and public health. 2022;19(4). | investigating age-related differences in dementia knowledge and attitudes |  |
| Leung AYM, Molassiotis A, Zhang J, et al. Dementia Literacy in the Greater Bay Area, China: Identifying the At-Risk Population and the Preferred Types of Mass Media for Receiving Dementia Information. Int J Environ Res Public Health. 2020;17(7). | evaluating area-realated differences in dementia literacy |  |
| Lo IL, Zeng W, Lei CI, et al. High School Students' Knowledge, Attitude and Preventive Practice of Dementia Care in Macao. Am J Alzheimers Dis Other Demen. 2020;35:1533317520948341. | investigating high school students in knowledge and attitudes and preventive practive of dementia care |  |
| Lo IL, Zeng W, Lei CI, et al. Knowledge, Attitude and Preventive Practice on Dementia Care among Primary Health Professionals in Macao. J Prev Alzheimers Dis. 2020;7(2):83-86. | investigating primary health professionals in knowledge and attitudes and preventive practive of dementia care |  |
| Iek Long L, Wen Z, Chin Ion L, et al. Macao Dementia Policy: Challenges and prospects (innovative practice). Dementia (London). 2021;20(2):791-795. | introducing the need and implementation of dementia policy |  |
| Xiao LD, Wang J, Ratcliffe J, et al. A nurse-led multicentre randomized controlled trial on effectiveness and cost-effectiveness of Chinese iSupport for dementia program: A study protocol. Journal of advanced nursing. 2022;78(5):1524-1533. | Assess the efficacy and cost-effectiveness of the iSupport for Dementia initiative in Australia and Greater China |  |
| Liao W, Tang JY, Lei M, et al. Chinese expert consensus on the development of dementia-friendly communities in Guangdong-Hong Kong-Macao Greater Bay Area [in Chinese]. Chinese Journal of Neuropsychiatric Diseases. 2022;4:193-205. | providing the practice standard for developing a dementia-friendly society in China via expert consensus; public promoton | early diagnosis & treatment; pharmacotherapy |
| Wu JW, Xie SL, Zhuang YZ, et al. A study on the chineseization and reliability of the attitudes towards dementia and their care scale [in Chinese]. Journal of Nursing of the People's Liberation Army. 2020;11:30-33. | evaluating the effectiveness of the scale of attitudes toward people with dementia and their care |  |
| Zeng W, Yi IQ. Analysis of initial dementia screening among elderly people in the Macao community [in Chinese]. Journal of Nursing Management, 2016;5:339-340. | examining the early stage dementia of the elderly in the community, as well as the care needs of those with dementia and their families |  |
| Leung AY, Leong CS, Cheong PL, Tsang AS, Nogueira BO, Liu M, Van IK, Cheung D, Xie YJ, Loke AY, Molassiotis A. Investigating dementia literacy among community-dwelling adults and older adults in Macau: A mixed methods study [Preprint]. Research Square. 2019. available at https://doi.org/10.21203/rs.2.18630/v1 | investigating Macao citizens in dementia knowledge, attitudes towards people with dementia, and help-seeking behaviours and intention toward dementia |  |
| Wu JW, Yi YQ, Wei MZ. Trend analysis of dementia prevalence and incidence in Macau [in Chinese]. Macau Journal of Nursing. 2021;20:1. | estimating the prevalence and incidence of dementia in Macao and analyzing its pattern of development for policy development |  |
| Wu JW, Wei MZ, Liang SM, et al. Analysis and implications of a dementia screening program for the elderly in the Macau community [in Chinese]. Macau Journal of Nursing. 2021;20:1. | the need of strengthening early detection and diagnosis of dementia and selecting the most suitable screening tools |  |
| **KWNC research/projects** |  |  |
| Zeng W. Dementia community services for the elderly action research project on upgrading community support services and care training venues for demented elders [in Chinese]. 2016. Available at: http://www2.kwnc.edu.mo/?page_id=1078 Accessed January 17, 2023. | evaluate the efficacy of community support services for dementia patients, as well as the content and forms of training for informal and family caregivers |  |
| Li WY, Zhou JQ. Exploring the localization of a home-based dementia caregiver support program [in Chinese]. 2018. Available at: http://www2.kwnc.edu.mo/?page_id=1078 Accessed January 17, 2023. | exploring the feasibility of the Dementia Caregiver Support Plan |  |
| Wu JW, Yi YQ, Liang SM, et al. Survey on Macau public's awareness and attitude towards dementia [in Chinese]. 2019. Available at: http://www2.kwnc.edu.mo/?page_id=1078 Accessed January 17, 2023. | comprehending the knowledge and attitudes of Macao residents towards dementia |  |
| Caritas Macau, Beijing Normal University & Hong Kong Baptist University United International College. Study on the community and home living conditions of demented elderly in Macau [in Chinese]. 2021. Available at: caritas.org.mo/uploads/ueditor/file/20210803/1627977377580681.pdf Accessed January 17, 2023. | Comprehending the circumstances of dementia patients and their caregivers in the community and at home |  |
| Yu HL, Aimei M. The development of health care services for older adults in Macao after Macao's return to China [in Chinese]. Macao Nursing Journal. 2019;31:46-52. Available at: http://www.kwnc.edu.mo/Journal/FullText/MJN_2019_Vol31/MJN_2019_Vol31.pdf | assessing the evolution of senior health care services in Macau since 1999; discussed the challenges facing health care services for the elderly |  |
| Iek LL. Building Dementia friendly Macao by trans-institutes and multidisciplinary collaborations [in Chinese]. Macao Nursing Journal. 2019;31:53-58. Available at: http://www.kwnc.edu.mo/Journal/FullText/MJN_2019_Vol31/MJN_2019_Vol31.pdf | introducing the measures and experiences in the prevention and treatment of dementia in Macao and inter-departmental multi-disciplinary collaboration |  |
| Jian WW, Man CV, Iat KV. The impact of education and interdisciplinary collaboration on the Dementia policy development in Macao [in Chinese]. Macao Nursing Journal. 2019;31:59-65. Available at: http://www.kwnc.edu.mo/Journal/FullText/MJN_2019_Vol31/MJN_2019_Vol31.pdf | summarising the role of education and multidisciplinary collaboration in the development of Macau's dementia policy in recent years |  |
| Sok ML, Iat KV. The establishment of nursing and health care curriculum in an ageing society [in Chinese]. Macao Nursing Journal. 2019;31:25-34. Available at: http://www.kwnc.edu.mo/Journal/FullText/MJN_2019_Vol31/MJN_2019_Vol31.pdf | establishing varied care programs to meet the demands of society |  |
| Leung AY, Molassiotis A, Zhang J, et al. Dementia literacy in Greater Bay Area, China: Identifying the at-risk population and the preferred mass media for receiving dementia information. Int J Environ Res Public Health. 2020;17(7):2511. | investigating Macao citizens in dementia knowledge, attitudes towards people with dementia, and help-seeking behaviours and intention toward dementia |  |
| Chuang Y, Van I, Zhao Y, et al. Icariin ameliorate Alzheimer’s disease by influencing SIRT1 and inhibiting Aβ cascade pathogenesis. Journal of Chemical Neuroanatomy. 2021;117:102014. |  | treatment - the effects and mechanism of icarii |
| Wu J, Leong SM, Che SL, et al. Comparisons of dementia knowledge and attitudes among the youth and older adults: Insights from the construal level theory perspective. International journal of environmental research and public health. 2022;19(4). | investigating age-related differences in dementia knowledge and attitudes |  |
| Chuang YC. To formulate Traditional Chinese Medicine constitution differentiation nursing for older people with cognitive impairment in Macau based on constitutional identification theory. Macao Nursing Journal. 2022;21:22-26. | based on constitutional identification theory to formulate the best care for cognitive impairment pateints |  |
| **MUST research** | | |
| Li XA, Ho YS, Chen L, et al. The protective effects of icariin against the homocysteine-induced neurotoxicity in the primary embryonic cultures of rat cortical neurons. Molecules. 2016;21(11):1557. |  | treatment - icariin promotes neuroprotective effects |
| Law BY, Wu AG, Wang MJ, et al. Chinese medicine: a hope for neurodegenerative diseases?. Journal of Alzheimer's Disease. 2017;60(s1):S151-60. |  | treatment - potential neuroprotective herbal compounds |
| Zhong XM, Ren XC, Lou YL, et al. Effects of in-vitro cultured calculus bovis on learning and memory impairments of hyperlipemia vascular dementia rats. Journal of ethnopharmacology. 2016; 192:390-7. |  | treatment - potential natural products for improving the abilities of learning and memory |
| Ni Y, Li L, Zhang W, et al. Discovery and LC-MS characterization of new crocins in Gardeniae Fructus and their neuroprotective potential. Journal of agricultural and food chemistry. 2017;65(14):2936-46. |  | treatment - evaluation of crocins in neuroprotective effects |
| Zhang WY, Feng XL, Lu D, et al. New lignans attenuating cognitive deterioration of Aβ transgenic flies discovered in Acorus tatarinowii. Bioorganic & Medicinal Chemistry Letters. 2018;28(4):814-9. |  | treatment - lignan can lleviate the cognitive deterioration |
| Yee A, Tsui NB, Chang YN, et al. Alzheimer’s disease: insights for risk evaluation and prevention in the Chinese population and the need for a comprehensive programme in Hong Kong/China. Hong Kong Med J. 2018;24(5):492-500. | risk evaluation, prevention and the need for social and psychological care |  |
| Yu L, Wu AG, Wong VK, et al. The new application of UHPLC-DAD-TOF/MS in identification of inhibitors on β-amyloid fibrillation from Scutellaria baicalensis. Frontiers in pharmacology. 2019;10:194. |  | screening - new application of identification of active compounds |
| Liu H, Liu X, Zhou S, et al. Disclosing the template-induced misfolding mechanism of tau protein by studying the dissociation of the boundary chain from the formed tau fibril based on a steered molecular dynamics simulation. ACS Chemical Neuroscience. 2019;10(3):1854-65. |  | pathogenesis |
| Ribaudo G, Coghi P, Zanforlin E, et al. Semi-synthetic isoflavones as BACE-1 inhibitors against Alzheimer’s disease. Bioorganic Chemistry. 2019;87:474-83. |  | treatment - noval drug class to prevent and treat AD |
| Sheng F, Zhang L, Wang S, et al. Deacetyl ganoderic acid F inhibits LPS-induced neural inflammation via NF-κB pathway both in vitro and in vivo. Nutrients. 2019;12(1):85. |  | treatment - potential therapeutic effects on neurogenerative disease (DeGA F) |
| Osama A, Zhang J, Yao J, et al. Nrf2: a dark horse in Alzheimer's disease treatment. Ageing Research Reviews. 2020;64:101206. |  | treatment - the role of Nrf2 (nuclear factor-erythroid 2-p45 derived factor 2) in AD |
| Mok SW, Wong VK, Lo HH, et al. Natural products-based polypharmacological modulation of the peripheral immune system for the treatment of neuropsychiatric disorders. Pharmacology & Therapeutics. 2020;208:107480. |  | treatment - potential natural product for neuropsychiatric disorders |
| Zhang AH, Ma ZM, Kong L, et al. High‐throughput lipidomics analysis to discover lipid biomarkers and profiles as potential targets for evaluating efficacy of Kai‐Xin‐San against APP/PS1 transgenic mice based on UPLC–Q/TOF–MS. Biomedical Chromatography. 2020;34(2):e4724. |  | treatment - potential targets |
| Wu AG, Zhou XG, Qiao G, et al. Targeting microglial autophagic degradation in NLRP3 inflammasome-mediated neurodegenerative diseases. Ageing research reviews. 2021;65:101202. |  | treatment - potential targets |
| Chuang Y, Van I, Zhao Y, et al. Icariin ameliorate Alzheimer’s disease by influencing SIRT1 and inhibiting Aβ cascade pathogenesis. Journal of Chemical Neuroanatomy. 2021;117:102014. |  | treatment - the effects and mechanism of icarii |
| Yee A, Tsui NB, Kwan RY, et al. Apolipoprotein E Gene Revisited: Contribution of Rare Variants to Alzheimer’s Disease Susceptibility in Southern Chinese. Current Alzheimer Research. 2021;18(1):67-79. |  | screening - investigate all gene variations for APOE (potention risk stratification biomarker in addition to APOE-ε4) |
| Ng RC, Jian M, Ma OK, et al. Chronic oral administration of adipoRon reverses cognitive impairments and ameliorates neuropathology in an Alzheimer’s disease mouse model. Molecular Psychiatry. 2021;26(10):5669-89. |  | treatment - adiponectin agonist may be a treatment for AD |
| Zhang Z, Chuang Y, Ke X, et al. The influence of TCM constitutions and neurocognitive function in elderly Macau individuals. Chinese Medicine. 2021;16(1):1-8. | different constitutions may associate the decline of neurocognitive function in senior adults |  |
| Sun WX, Zhang CT, Yu XN, et al. Preparation and pharmacokinetic study of diosmetin long-circulating liposomes modified with lactoferrin. Journal of Functional Foods. 2022;91:105027. |  | treatment - novel drug transportation to deliver diosmetin, potential AD treatment |
| Li HL, Zhang SY, Ren YS, et al. Identification of Ellagic Acid and Urolithins as Natural Inhibitors of Aβ25-35-induced Neurotoxicity and the Mechanism Predication Using Network Pharmacology Analysis and Molecular Docking. Frontiers in nutrition. 2022:1761. |  | treatment - natural products promote neuroprotective effects |
| Zhang L, Yong YY, Deng L, et al. Therapeutic potential of Polygala saponins in neurological diseases. Phytomedicine. 2022:154483. |  | treatment - potential TCM for neurological disease |
| Tang B, Zeng W, Song LL, et al. Extracellular Vesicle Delivery of Neferine for the Attenuation of Neurodegenerative Disease Proteins and Motor Deficit in an Alzheimer’s Disease Mouse Model. Pharmaceuticals. 2022;15(1):83. |  | treatment - the encapsulation of natural compounds using exosomes |
| Yan L, Guo MS, Zhang Y, et al. Dietary Plant Polyphenols as the Potential Drugs in Neurodegenerative Diseases: Current Evidence, Advances, and Opportunities. Oxidative Medicine and Cellular Longevity. 2022;2022. |  | treatment - development of the neuroprotective effect of the most widely reported dietary plant polyphenols |
| Zhou M, Wu X, Li Y, et al. Atomic zinc sites with hierarchical porous carbon for high-throughput chemical screening with high loading capacity and stability. Pharmacological Research. 2022;178:106154. |  | treatment - turmeric (A beta aggregation inhibitors) |
| Jiang A, Anyanwu M, Leong K, et al. 3-[(1H-Benzo [d][1, 2, 3] triazol-1-yl) oxy] propyl 9-hydroxy-5a, 5b, 8, 8, 11a-pentamethyl-1-(prop-1-en-2-yl) icosahydro-3aH-cyclopenta [a] chrysene-3a-carboxylate. Molbank. 2022;2022(3):M1419. |  | treatment - drug discovery |
| Fai LK, Anyanwu M, Ai J, et al. 4-(4-(((1 H-Benzo [d][1, 2, 3] triazol-1-yl) oxy) methyl)-1 H-1, 2, 3-triazol-1-yl)-7-chloroquinoline. Molbank. 2022;2022(3):M1404. |  | treatment - new therapeutic options to counteract neurodegeneration |
| **MPU research** | | |
| Lam Nogueira BO, Li L, Meng LR, et al. Clinical characteristics and quality of life of older adults with cognitive impairment in Macao. Psychogeriatrics. 2018 ;18(3):182-189. | social needs - further appropriate strategies and need to improve access to treatment |  |
| Leung AYM, Molassiotis A, Zhang J, et al. Dementia Literacy in the Greater Bay Area, China: Identifying the At-Risk Population and the Preferred Types of Mass Media for Receiving Dementia Information. Int J Environ Res Public Health. 2020;17(7). | evaluating area-realated differences in dementia literacy |  |
| Jiang M, Yan B, Li Y, et al. Image Classification of Alzheimer’s Disease Based on External-Attention Mechanism and Fully Convolutional Network. Brain Sciences. 2022;12(3):319. |  | screening - automatic and accurate classification of Alzheimer's disease |
